# E-Cigarette Sales to School-Uniformed Adolescents in China: A Randomized Clinical Trial

**DOI:** 10.1001/jamanetworkopen.2025.35623

**Published:** 2025-10-10

**Authors:** Yang Wang, Xiaoyang Lv, Linnea I. Laestadius, Jeanine P. D. Guidry, Elham Mahmoudi, Grace Kong, Jie Chang, Adam Martin, Miaoqing Yang, Duo Yan, Lei Si, Arturo V. Bustamante, Hai Fang

**Affiliations:** 1 China Center for Health Development Studies, Peking University, Beijing, China; 2 Faculty of Health and Wellness, City University of Macau, Macau, China; 3 School of Public Health, Peking University, Beijing, China; 4 Zilber College of Public Health, University of Wisconsin, Milwaukee; 5 Department of Communication and Cognition, Tilburg University, Tilburg, the Netherlands; 6 Department of Family Medicine, Michigan Medicine, University of Michigan, Ann Arbor; 7 Department of Psychiatry, Yale School of Medicine, New Haven, Connecticut; 8 Department of Pharmacy Administration and Clinical Pharmacy, School of Pharmacy, Xi’an Jiaotong University, Xi’an, China; 9 Leeds Institute of Health Sciences, University of Leeds, Leeds, United Kingdom; 10 School of Health Sciences, Western Sydney University, Campbelltown, New South Wales, Australia; 11 Translational Health Research Institute, Western Sydney University, Penrith, New South Wales, Australia; 12 Department of Health Policy and Management, Fielding School of Public Health, University of California, Los Angeles; 13 Center for Health Policy Research, Fielding School of Public Health, University of California, Los Angeles; 14 National Health Commission Key Laboratory of Reproductive Health, Peking University, Beijing, China

## Abstract

**Importance:**

Electronic cigarettes (e-cigarettes) are appealing to adolescents, but sale of e-cigarettes to individuals younger than 18 years is prohibited in China. The effect of school uniforms on e-cigarette sales to adolescents has not been explored.

**Objective:**

To study e-cigarette sales to adolescents wearing school uniforms compared with those wearing casual attire.

**Design, Setting, and Participants:**

This 2-arm randomized clinical trial involved adolescent mystery shoppers wearing school uniforms or casual attire who attempted to purchase e-cigarettes. The trial was conducted across 36 major metropolitan areas in China from July 29 to September 3, 2023. Adolescent buyers aged 18 to 19 years were randomly assigned to wear 1 of the 2 attire types. A 1:1 matching of e-cigarette and cigarette stores was also performed.

**Intervention:**

Adolescent buyers attempting to purchase e-cigarettes while wearing school uniforms were the intervention group, and those in casual attire served as the control group.

**Main Outcomes and Measures:**

The primary outcome was successful purchase of e-cigarettes. Secondary outcomes assessed whether sellers verbally inquired about the buyer’s age, requested an identification (ID) card for age verification, or dissuaded the buyers from using e-cigarettes. Multivariable logistic regression models identified factors associated with outcomes by attire type, adjusted for buyer, seller, and store characteristics.

**Results:**

The final analytical sample included 1089 visits to e-cigarette stores (543 by adolescents in school uniforms and 546 by those in casual attire) and 1059 visits to cigarette stores. Of the e-cigarette stores visited, 85.4% (95% CI, 80.3%-89.4%) posted external advertising, 39.4% (95% CI, 35.9%-43.0%) were located in shopping malls, 90.8% (95% CI, 87.7%-93.2%) displayed age-of-sale signs, and only 15.0% (95% CI, 9.7%-22.5%) exhibited health warnings specifically for e-cigarettes. The overall success rate of adolescents purchasing e-cigarettes without age verification was 78.3% (95% CI, 75.8%-80.6%), significantly lower than the 94.5% (95% CI, 91.3%-96.6%) success rate for purchasing cigarettes without age verification. Adolescents wearing school uniforms had significantly lower odds of successfully purchasing e-cigarettes (adjusted odds ratio [AOR], 0.39; 95% CI, 0.23-0.66) than those in casual attire and were more likely to be asked about their age (AOR, 9.18; 95% CI, 6.46-13.06), requested to show an ID card (AOR, 6.68; 95% CI, 4.53-9.87), and dissuaded from using e-cigarettes (AOR, 1.79; 95% CI, 1.30-2.47). Additionally, when sellers requested ID cards, the probability of successful e-cigarette purchases was significantly lower than when they did not (AOR, 0.02; 95% CI, 0.01-0.03).

**Conclusions and Relevance:**

In this randomized clinical trial of e-cigarette sales, e-cigarettes remained widely sold to adolescents without age verification in China, but wearing a school uniform appeared to reduce adolescents’ ability to purchase e-cigarettes.

**Trial Registration:**

ClinicalTrials.gov Identifier: NCT05962411

## Key Points

**Question:**

Are there differences in e-cigarette sales to adolescents wearing school uniforms compared with those in casual attire?

**Findings:**

In this randomized clinical trial involving 1089 e-cigarette stores in China, adolescents wearing school uniforms had significantly lower odds of successfully purchasing e-cigarettes than those wearing casual attire. In addition, they were significantly more likely to be asked about their age, requested to show an identification card, and dissuaded from using e-cigarettes.

**Meaning:**

The findings suggest wearing school uniforms may reduce adolescents’ ability to purchase e-cigarettes.

## Teaser

This randomized clinical trial investigates sales of e-cigarettes to adolescents wearing school uniforms vs casual attire in e-cigarette stores across major metropolitan areas in China.

## Introduction

Electronic cigarettes (e-cigarettes) have become increasingly prevalent worldwide,^1^ particularly appealing to adolescents due to curiosity, attractive flavors, peer pressure, and effective marketing.^2,3,4^ The use of e-cigarettes is harmful to adolescents’ health.^5,6,7^ Youths who try 1 form of tobacco or nicotine product are more likely to try others, contributing to broader patterns of polytobacco use.^8^ In China, 16.1% of junior and senior high school students had experimented with e-cigarettes in 2021.^9^ The Chinese Minors Protection Law prohibits the sales of cigarettes and e-cigarettes to adolescents younger than 18 years, requiring sellers to check identification (ID) cards to verify buyers’ ages.^10^

The commercial availability of tobacco and nicotine products to adolescents can be reduced primarily by improving sellers’ compliance with minimum age sales restrictions through active enforcement. For example, in the US, the Tobacco 21 legislation requires states to conduct random compliance inspections of local retailers to enforce the minimum legal sales age.^11^ Penalties, including fines and license suspension, have been associated with dramatically reduced retailer violation rates.^12,13^ In China, the e-cigarette market has grown rampantly due to previous lack of regulations.^10,14,15^

The practice of requiring students to wear a school uniform varies across countries, reflecting cultural and educational values.^16^ In China, many primary and secondary schools mandate that students wear school uniforms while attending school. School uniforms could lead to better discipline, academic performance, and class involvement.^17,18^ Given that uniforms enable e-cigarette sellers to more easily identify school-aged adolescents, this study aimed to measure the impact of school uniforms on e-cigarette sales to adolescents in China.

## Methods

### Study Design

This 2-arm randomized clinical trial (RCT) measured the impact of adolescent buyers’ type of attire (school uniforms vs casual attire) on the likelihood of successful e-cigarette purchases from e-cigarette stores in all major metropolitan areas in mainland China. We also conducted a 1:1 match of e-cigarette to cigarette stores to allow for comparisons. The participants in this study were anonymous e-cigarette and tobacco retail stores rather than individual human participants. Sellers were not aware of their participation due to the mystery shopping approach adopted. After reviewing the research protocol, the Peking University institutional review board deemed this study exempt from review and informed consent.^19,20^ This RCT is registered at clinicaltrials.gov (NCT05962411). The trial protocol has been included as Supplement 1. We followed the Consolidated Standards of Reporting Trials (CONSORT) reporting guideline.

### Intervention and Control

The trial intervention and control involved simulated adolescent buyers wearing school uniforms or casual attire while attempting to purchase e-cigarettes. In 2015, China’s Ministry of Education issued a directive that encourages students to wear school uniforms on weekdays, although it is not uniformly mandated nationwide.^21^ In China, the decision to implement school uniforms is typically made jointly by schools and parent committees. Once adopted, uniform policies become mandatory for all students in the school. This participatory process allows parents to express their views—including concerns about affordability and individual expression—before a final decision is made. As such, the process is intended to reflect both community values and financial feasibility. Based on signaling theory, school uniforms serve as clear cues of adolescent status.^22,23^ School uniforms visually signal the student identity of potential buyers to sellers immediately and may also heighten concern about being caught for underage sales, potentially making sellers more likely to verify age and more hesitant to sell e-cigarettes. Adolescents’ refusal to present an ID may further raise seller suspicion, leading to reluctance to risk possible illegal underage sales. There were 2 main hypotheses in this study: first, that adolescents in school uniforms vs casual attire would be less likely to successfully purchase e-cigarettes, and second, that adolescents in school uniforms would be more likely to be requested to show their ID for age verification than adolescents in casual attire.

### Identification of E-Cigarette and Cigarette Stores

Lacking officially released information of licensed e-cigarette stores, we compiled a list of brick-and-mortar stores using the most popular Chinese navigation tool, AutoNavi map, with the location and contact information scraped using the tool’s application programming interface. This process was conducted for all 36 metropolitan areas across mainland China, including all 27 provincial capitals, 4 municipalities directly under the central government, and 5 cities specially designated in the national plan. Identified stores were contacted by telephone during business hours to confirm they were still operating as e-cigarette vendors and to verify their addresses, with each store being called up to 2 times. The list of e-cigarette stores for each metropolitan area was first randomized to enhance representativeness. Stores were then contacted in randomized order to confirm eligibility and operational status until the target number of stores (usually 40 per city) was reached. For each e-cigarette store, 1 nearby cigarette store was matched using a convenience sampling approach .

### Identification and Randomization of Adolescent Mystery Buyers

Considering that the World Health Organization (WHO) defines adolescents as individuals aged 10 to 19 years,^24^ we recruited students aged 18 to 19 years as adolescent mystery buyers from universities in Beijing through social media. It is challenging for e-cigarette sellers to determine whether buyers are under or above the minimum smoking age of 18 years based on facial features alone.^25^ We could not recruit adolescents younger than 18 years, as it is illegal for them to purchase e-cigarettes in China. Other requirements for mystery buyers included having never used either e-cigarettes or cigarettes and not having dyed hair or visible tattoos, as these criteria helped minimize bias in store responses and ensure consistency across purchase attempts.^26^

To improve data collection efficiency, all e-cigarette stores in each metropolitan area were first mapped and grouped into geographic clusters (typically 4 stores per cluster) based on location and transportation accessibility. Adolescent buyers were then randomly assigned through Microsoft Excel, version 16.75 (Microsoft Corporation), to these store clusters for their field visits. Only 1 purchase was attempted for each store. Standardized attire and appropriate bags were provided to each adolescent at no cost (eFigure 2 in Supplement 2). The school uniform, designed in a style commonly worn by high school students in China, served as a visible signal of student status. The type of attire worn on each visit—school uniform or casual clothing—was randomly assigned by the research team in advance within each sex group, ensuring that each adolescent had an equal probability of wearing either attire type across the study period.

### Study Outcomes and Data Collection

The primary outcome was the successful purchase of e-cigarette products by adolescent buyers without age verification. Secondary outcomes included sellers verbally inquiring about the adolescent buyer’s age, requesting to show an ID card for age verification, and dissuading the adolescent buyer from using e-cigarettes. During the fieldwork from July 29 to September 3, 2023, data were recorded using a self-developed instrument embedded in an online survey platform. The instrument was designed primarily based on the Vape Shop Standardized Tobacco Assessment for Retail Settings (V-STARS) and the Tobacco Advertising, Promotion and Sponsorship (TAPS) bans compliance guide.^27,28^

Adolescents without makeup or facial hair attempted to buy e-cigarettes.^26,29,30^ Upon arriving at each store, they first recorded all observable features from outside without being noticed. After entering, adolescents documented whether the seller verbally inquired about their age, requested to show an ID card for age verification, and dissuaded them from using e-cigarettes during the shopping process. The adolescent buyers, if asked about their age, would claim to be adults but without carrying an ID, to mimic a typical everyday situation.^31,32^ Therefore, the age of all the buyers was not verified by the sellers. When dissuaded from using e-cigarettes, the buyers would still insist on purchasing. The adolescents then noted whether they could successfully purchase the lowest-priced e-cigarette product available in the store. To reduce potential biases, we developed a soft script to standardize in-store interactions as much as possible. After the observation and interaction (lasting 10-15 minutes), the adolescent immediately left the store and completed the rest of the data entry to optimize retention.

### Statistical Analysis

The unit of analysis was the encounter attempting to purchase e-cigarettes (or cigarettes, in matched cigarette stores). To estimate the sample size required to detect differences in successful purchase rates between adolescents wearing school uniforms and those in casual attire, we conducted a power calculation based on a cluster randomized trial design. Assuming an α value of .05, power of 0.85, an intracluster correlation coefficient of 0.5, and purchase success rates of 70% for the school uniform group and 90% for the casual attire group (based on unpublished pilot data from Beijing and Shijiazhuang), the minimum required sample size was calculated to be 352 stores. This estimate assumed a cluster size of 4 e-cigarette stores visited per adolescent per day. To enhance national representativeness and allow for more precise estimates across diverse metropolitan areas, the final sample size was increased to 1056 stores—3 times the minimum required.

We described the distributions of e-cigarette seller behavior outcomes, store environment features, and the demographic characteristics of buyers and sellers across the 2 types of attire. Rates of successful e-cigarette purchases, stratified by age-checking behaviors and type of attire, were also reported. χ^2^ tests were used to detect if these differences were statistically significant. For matched e-cigarette and cigarette stores, we used the McNemar test to assess the distinctions in outcome variables. Multivariable logistic regression models were adopted to identify factors associated with the primary outcome and 3 secondary outcomes, with a particular focus on the type of buyer attire. All models controlled for the sex and age of the buyer; store-level variables, including location, external advertising, age-of-sale warning signage, retail license status, and presence of e-cigarette health warnings; seller characteristics, including perceived age and sex; region of mainland China (western, central, or eastern); and day of visit (weekday vs weekend). We also conducted sex-stratified analyses to assess potential effect modification. Clustering at the level of adolescent buyers was accounted for in all the statistical analyses. The analyses were performed using Stata/MP, version 17 (StataCorp LLC). A 2-sided *P* value of less than .05 was considered statistically significant.

## Results

### Descriptive Statistics of Study Samples

**Figure 1.**
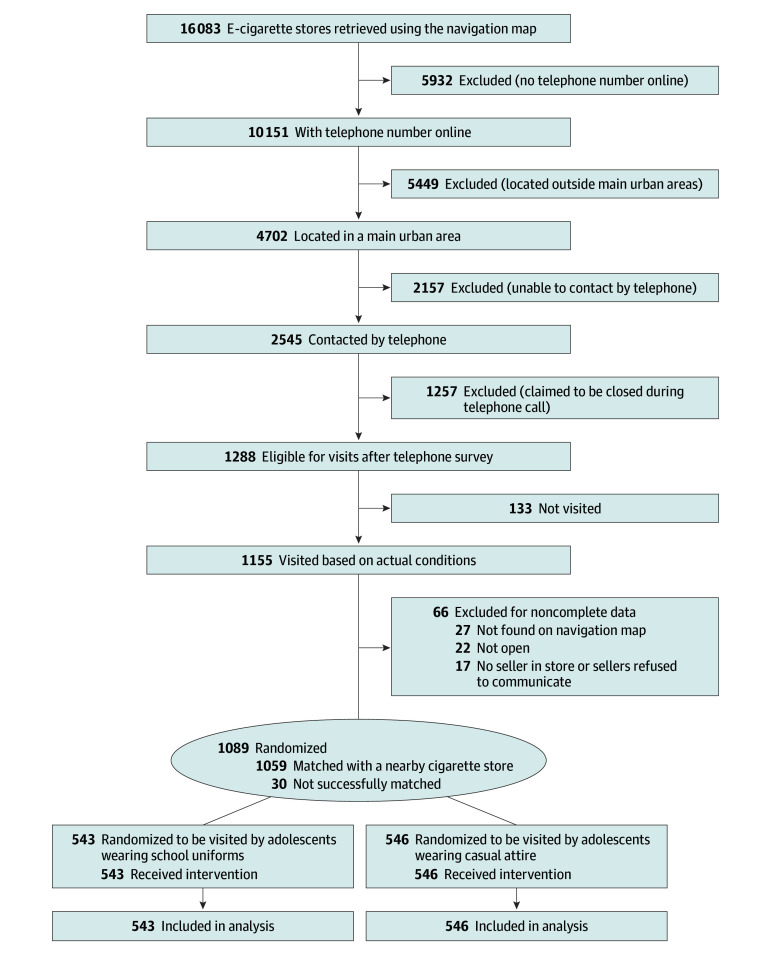
Study Flow Diagram Stores were located across 36 major metropolitan areas in China.

A total of 16 803 brick-and-mortar e-cigarette stores were identified, of which 1155 were visited. Data collection was completed in 1089 of those stores; noncompletion was due to stores being unlocated (n = 27) or closed (n = 22) or sellers not being present or refusing to communicate (n = 17). Of the 1089 included e-cigarette stores, 1059 (97.2%) were successfully matched with a cigarette store (Figure 1 and eFigure 1 in Supplement 2).

Out of 1089 e-cigarette store visits by 25 adolescents, the mean age of the adolescent across visits was 19.2 years (95% CI, 19.2-19.3 years); 51.7% (95% CI, 31.5%-71.4%) of the visits were by females and 48.3% (95% CI, 28.6%-68.5%) by males (e-cigarette store characteristics are shown in Table 1 and matched cigarette store characteristics in eTable 1 in Supplement 2). Of the 1089 visits, 543 were by adolescents in school uniforms and 546 by adolescents in casual attire. Among the e-cigarette stores, 85.4% (95% CI, 80.3%-89.4%) posted external advertising, usually featuring images of fruits, scenery, or vaping products. On-street stores accounted for 60.6% (95% CI, 57.0%-64.1%) of the stores, and the rest (39.4%; 95% CI, 35.9%-43.0%) were located in shopping malls. Age-of-sale signs and licenses were exhibited in 90.8% (95% CI, 87.7%-93.2%) and 86.7% (95% CI, 82.7%-89.9%) of the e-cigarette stores, respectively. Only 15.0% (95% CI, 9.7%-22.5%) of the stores displayed health warnings specifically for e-cigarettes, and 14.4% (95% CI, 10.1%-20.1%) had general warnings for tobacco products. For e-cigarette sellers, most were perceived by the adolescent buyers to be under the age of 40 years (40.5% [95% CI, 33.4%-48.0%], 18 to <30 years; 42.5% [95% CI, 37.3%-47.9%], 30 to <40 years); 51.3% (95% CI, 48.1%-54.6%) were identified by buyers as female and 48.7% (95% CI, 45.4%-52.0%) as male.

**Table 1.** Buyer, Store, and Seller Characteristics by Types of Attire for E-Cigarette Store Visits

Characteristic	E-cigarette store visits, % (95% CI)		
	Overall (n = 1089)	School uniforms (n = 543)	Casual attire (n = 546)
**Buyer**			
Age, mean (95% CI), y^a^	19.2 (19.2-19.3)	19.2 (19.2-19.3)	19.2 (19.2- 19.3)
Sex^a^			
Female	51.7 (31.5-71.4)	51.0 (30.8-70.9)	52.4 (32.0-72.0)
Male	48.3 (28.6-68.5)	49.0 (29.1-69.2)	47.6 (28.0-68.0)
**Store**			
Location			
On street	60.6 (57.0-64.1)	62.2 (57.0-67.2)	59.0 (54.6-63.3)
In mall	39.4 (35.9-43.0)	37.8 (32.8-43.0)	41.0 (36.8-45.4)
External advertising			
No	14.6 (10.6-19.7)	15.1 (10.7-20.8)	14.1 (9.8-19.9)
Yes	85.4 (80.3-89.4)	84.9 (79.2-89.3)	85.9 (80.2-90.2)
Age-of-sale warning sign			
No	9.2 (6.8-12.4)	8.7 (5.9-12.6)	9.7 (6.6-14.0)
Yes	90.8 (87.7-93.2)	91.3 (87.4-94.1)	90.3 (86.0-93.4)
License			
No	13.3 (10.1-17.3)	14.9 (11.3-19.5)	11.7 (8.1-16.6)
Yes	86.7 (82.7-89.9)	85.1 (80.5-88.8)	88.3 (83.4-91.9)
E-cigarette health warning			
No	85.0 (77.5-90.4)	85.5 (77.4-91.0)	84.6 (76.5-90.3)
Yes	15.0 (9.7-22.5)	14.6 (9.0-22.6)	15.4 (9.7-23.5)
**Seller**			
Perceived age, y			
18 to <30	40.5 (33.4-48.0)	37.8 (30.4-45.7)	43.2 (35.1-51.7)
30 to <40	42.5 (37.3-47.9)	44.9 (38.7-51.3)	40.1 (34.1-46.5)
≥40	17.0 (13.3-21.5)	17.3 (13.2-22.4)	16.7 (12.8-21.5)
Perceived sex			
Female	51.3 (48.1-54.6)	52.3 (48.5-56.1)	50.4 (45.1-55.6)
Male	48.7 (45.4-52.0)	47.7 (43.9-51.5)	49.6 (44.4-54.9)
**Other**			
Region			
Western	30.5 (20.4-42.9)	29.5 (19.1-42.5)	31.5 (21.0-44.3)
Central	23.2 (20.9-25.7)	23.6 (19.9-27.8)	22.9 (19.2-27.1)
Eastern	46.3 (34.3-58.8)	47.0 (34.4-60.0)	45.6 (33.5-58.2)
Day of visit			
Workday	65.4 (63.0-67.7)	65.6 (60.1-70.6)	65.2 (60.4-69.7)
Weekend	34.6 (32.3-37.1)	34.4 (29.4-39.9)	34.8 (30.3-39.6)

^a^ The distributions of buyers’ ages (mean) and sex (proportion) were averaged across all the attempted purchases.

### Bivariable Analyses

**Figure 2.**
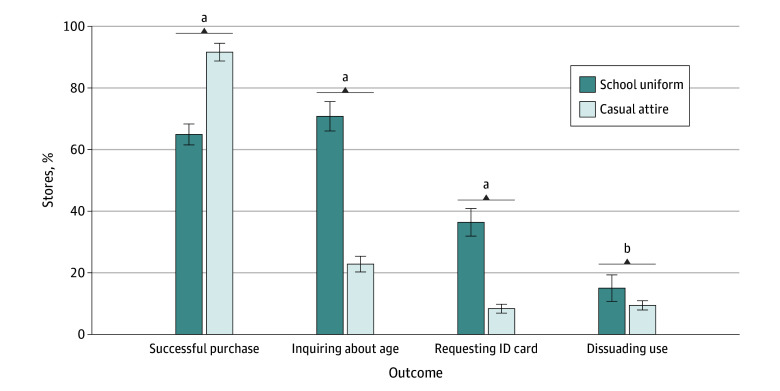
Differences in E-Cigarette Sales to Adolescents by Type of Attire Error bars represent 95% CIs. ^a^*P* < .001. ^b^*P* < .01.

While 91.8% (95% CI, 88.2%-94.3%) of adolescent buyers in casual attire successfully purchased e-cigarettes, the proportion of those in school uniforms who made successful purchases was only 65.0% (95% CI, 61.4%-68.5%) (*P* < .001) (Figure 2). Adolescents in school uniforms were significantly more likely than those in casual attire to be verbally asked about their age (70.9% [95% CI, 65.6%-75.7%] vs 22.9% [95% CI, 18.0%-28.6%]; *P* < .001), requested to show an ID card (36.5% [95% CI, 31.9%-41.3%] vs 8.4% [95% CI, 5.9%-11.9%]; *P* < .001), and dissuaded from using e-cigarettes (15.1% [95% CI, 11.1%-20.2%] vs 9.5% [95% CI, 6.8%-13.1%]; *P* = .002). The probabilities of successful purchases from sellers who did not inquire about the buyers’ ages or request to show an ID card consistently remained above 90.0% (eFigure 3 in Supplement 2). The likelihood of e-cigarette sales to adolescents was significantly lower among sellers who inquired about the buyers’ ages compared with those who did not. This difference was more pronounced in the school uniform group (94.3% [95% CI, 88.9%-97.2%] vs 53.0% [95% CI, 49.3%-56.6%]; *P* < .001) than in the casual attire group (96.7% [95% CI, 93.6%-98.3%] vs 75.2% [95% CI, 64.3%-83.6%]; *P* < .001). The same pattern was observed for ID card requests in both the school uniform group (92.8% [95% CI, 88.4%-95.6%] vs 16.7% [V95% CI, 12.8%-21.5%]; *P* < .001) and the casual attire group (97.2% [95% CI, 94.8%-98.5%] vs 32.6% [95% CI, 20.0%-48.3%]; *P* < .001).

### Multivariable Logistic Regression Analyses

Adolescents wearing school uniforms had lower odds of successfully purchasing e-cigarettes (adjusted odds ratio [AOR], 0.39; 95% CI, 0.23-0.66) and higher odds of being verbally asked about their age (AOR, 9.18; 95% CI, 6.46-13.06), being requested to show an ID card (AOR, 6.68; 95% CI, 4.53-9.87), and being dissuaded from using e-cigarettes (AOR, 1.79; 95% CI, 1.30-2.47) compared with those wearing casual attire (Table 2). Being requested vs not requested to show an ID card for age verification (AOR, 0.02; 95% CI, 0.01-0.03; *P* < .001), rather than just being verbally asked vs not asked about their age (AOR, 0.65; 95% CI, 0.35-1.23; *P* = .19), resulted in lower estimated odds of successful e-cigarette purchases. However, in the matched cigarette stores, no difference was found in successful cigarette purchases across the 2 types of attire, even though adolescents in school uniforms were more likely to be verbally asked about their age, requested to show an ID card, and dissuaded from use in the cigarette stores (eTable 2 and eFigure 4 in Supplement 2).

**Table 2.** Multivariate Logistic Regressions for Successful E-Cigarette Purchases and Those Inquiring About Age, Requesting an ID Card, and Dissuading Use

Characteristic	Purchase							
	Successful (n = 1089)		Inquiring about age (n = 1089)		Requesting ID card (n = 1089)		Dissuading e-cigarette use (n = 945)^a^	
	AOR (95% CI)	*P* value	AOR (95% CI)	*P* value	AOR (95% CI)	*P* value	AOR (95% CI)	*P* value
**Buyer**								
Attire type								
Casual	1 [Reference]	NA	1 [Reference]	NA	1 [Reference]	NA	1 [Reference]	NA
School uniform	0.39 (0.23-0.66)	<.001	9.18 (6.46-13.06)	<.001	6.68 (4.53-9.87)	<.001	1.79 (1.30-2.47)	<.001
Age, per 1-y increase	1.18 (0.64-2.17)	.60	1.64 (0.92-2.90)	.92	1.39 (0.74-2.60)	.31	0.85 (0.34-2.10)	.72
Sex								
Female	1 [Reference]	NA	1 [Reference]	NA	1 [Reference]	NA	1 [Reference]	NA
Male	1.58 (1.02-2.45)	.04	1.10 (0.75-1.60)	.63	1.06 (0.72-1.56)	.76	0.57 (0.30-1.10)	.10
**Store**								
Location								
On street	1 [Reference]	NA	1 [Reference]	NA	1 [Reference]	NA	1 [Reference]	NA
In mall	0.55 (0.38-0.81)	.002	1.23 (0.94-1.60)	.13	1.22 (0.85-1.75)	.28	1.22 (0.76-1.96)	.41
External advertising	1.22 (0.49-2.99)	.67	1.14 (0.78-1.67)	.49	1.04 (0.69-1.57)	.86	0.90 (0.42-1.90)	.78
Age-of-sale warning sign	1.24 (0.57-2.67)	.59	1.79 (1.15-2.77)	.009	2.65 (1.54-4.55)	<.001	1.58 (0.88-2.84)	.13
License	0.72 (0.26-2.02)	.54	1.80 (1.24-2.61)	.002	1.68 (1.11-2.54)	.02	1.24 (0.76-2.00)	.39
E-cigarette health warning	1.71 (0.75-3.88)	.20	0.77 (0.49-1.22)	.26	0.95 (0.56-1.61)	.86	1.79 (1.09-2.96)	.02
**Seller**								
Perceived age, y								
18 to <30	1 [Reference]	NA	1 [Reference]	NA	1 [Reference]	NA	1 [Reference]	NA
30 to <40	1.04 (0.66-1.63)	.87	1.25 (0.94-1.66)	.12	1.20 (0.90-1.59)	.21	0.98 (0.59-1.64)	.95
≥40	2.24 (1.23-4.07)	.008	0.68 (0.44-1.05)	.09	0.77 (0.49-1.23)	.27	0.77 (0.41-1.44)	.41
Perceived sex								
Female	1 [Reference]	NA	1 [Reference]	NA	1 [Reference]	NA	1 [Reference]	NA
Male	0.81 (0.47-1.38)	.43	1.19 (0.93-1.52)	.17	1.36 (1.01-1.85)	.046	1.47 (0.93-2.32)	.10
**Other**								
Region								
Western	1 [Reference]	NA	1 [Reference]	NA	1 [Reference]	NA	1 [Reference]	NA
Central	1.61 (0.91-2.86)	.10	1.68 (1.05-2.67)	.03	0.70 (0.41-1.19)	.18	1.67 (1.00-2.80)	.050
Eastern	1.91 (1.19-3.06)	.008	1.53 (1.04-2.24)	.03	1.26 (0.76-2.08)	.37	0.87 (0.48-1.58)	.64
Day of visit								
Workday	1 [Reference]	NA	1 [Reference]	NA	1 [Reference]	NA	1 [Reference]	NA
Weekend	1.08 (0.68-1.70)	.74	0.86 (0.65-1.16)	.33	1.14 (0.83-1.57)	.42	1.17 (0.79-1.72)	.44
Inquiring vs not inquiring about age	0.65 (0.35-1.23)	.19	NA	NA	NA	NA	NA	NA
Requesting vs not requesting ID card	0.02 (0.01-0.03)	<.001	NA	NA	NA	NA	NA	NA

Abbreviations: ID, identification; NA, not applicable.

^a^ Some adolescent buyers who were asked to leave the store might still have been asked about their age or asked to present identification. Those who were asked to leave without any further buyer-seller communication were excluded from the analysis.

We conducted a parallel mediation analysis with being asked about age and being requested to show an ID card as mediators. The results showed that wearing a school uniform had a significant negative effect on successful purchases of e-cigarettes, which was partially mediated by ID card requests (eTable 3 in Supplement 2) but not by being asked about age. Subgroup analyses showed that the protective effects of wearing school uniforms remained consistently significant across age groups and regions, although the magnitudes varied (eTable 4 in Supplement 2).

### Outcomes for E-Cigarette and Cigarette Sales Regardless of Attire Type

**Figure 3.**
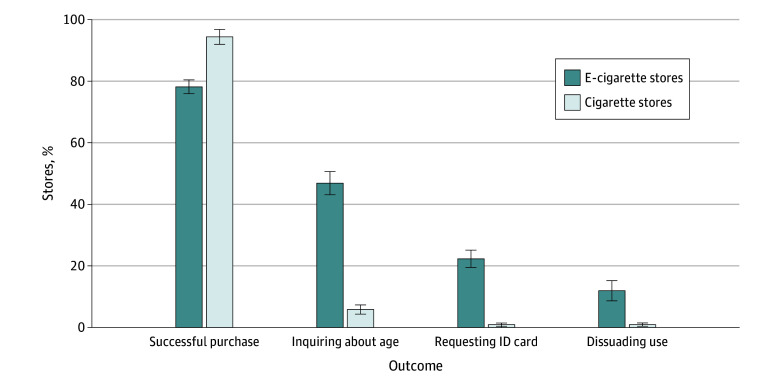
Differences in E-Cigarette and Cigarette Sales to Adolescents Regardless of Attire All differences were statistically significant (*P* < .001). Error bars represent 95% CIs.

The overall rate of successful purchases from e-cigarette stores without age verification was 78.3% (95% CI, 75.8%-80.6%), significantly lower than the 94.5% (95% CI, 91.3%-96.6%) success rate from cigarette stores, regardless of attire type (*P* < .001) (Figure 3). E-cigarette sellers were significantly more likely than cigarette sellers to verbally inquire about the buyers’ age (46.9% [95% CI, 43.0%-50.9%] vs 5.9% [95% CI, 4.5%-7.7%]; *P* < .001), request to show an ID card (22.4% [95% CI, 19.3%-25.8%] vs 0.9% [95% CI, 0.5%-1.7%]; *P* < .001), and dissuade buyers from using e-cigarettes (12.0% [95% CI, 8.9%-15.9%] vs 1.0% [95% CI, 0.5%-1.8%]; *P* < .001). When dividing mainland China into eastern, central, and western regions, adolescents from the central area had a higher probability of successful purchases (eFigure 5 in Supplement 2).

## Discussion

To our knowledge, this is the first study to assess e-cigarette retailer compliance with age-restricted sales and examine the effect of school uniforms worn by adolescents on these practices in China. Our findings suggest that most adolescents claiming to be adults could successfully purchase e-cigarette products from stores. School uniforms may increase the likelihood of adolescents being refused purchases, asked about their age, requested to show an ID card, and dissuaded from e-cigarette use by sellers. While sales of e-cigarettes to underage buyers represent regulatory noncompliance, actively dissuading an adolescent from using e-cigarettes may reflect the seller’s sense of responsibility and ethical awareness.

Of note, even in cases where vendors inquired about the buyer’s age, most still completed the sale. These findings highlight a serious compliance issue in the enforcement of age-restriction policies in China, where regulatory frameworks may exist but are inconsistently implemented at the point of sale. In the US, data from 238 stores in urban Colorado showed that the violation rate of selling e-cigarettes to minors was 17.6% in 2014-2015.^30^ A 2017 study in California reported that 13.1% of retailers illegally sold e-cigarettes to adolescents.^33^ Compared with these estimates, underage e-cigarette sales in the current study were substantially higher in China. Moreover, the probability of e-cigarette sellers requesting buyers to show ID cards for age verification in our study was 22.4%, much lower than the 75% and 59% observed in New Jersey and 6 metro areas across the US, respectively.^19,20^

School uniforms immediately signal to sellers the student identity of potential buyers and may also heighten concerns about being caught for underage sales, potentially making sellers more likely to verify age and more hesitant to sell e-cigarettes. We found e-cigarette sellers’ age-checking behaviors, particularly requesting to show ID cards, to be associated with lower probabilities of successful purchases, regardless of attire type. This might be because adolescents’ refusal to present ID further raised seller suspicion, leading to a reluctance to risk possible illegal underaged sales. Therefore, requesting ID needs to be mandated in China.^34^

Unlike e-cigarette sellers, the type of attire did not affect successful sales by cigarette sellers. The differences may be due to distinctions in store types and retailer training, as most e-cigarette stores exclusively sell e-cigarettes, while cigarette stores are usually multipurpose shops. Low compliance with age-based sales restrictions in cigarette stores may also reflect a lingering social norm in which adolescents are culturally permitted to purchase cigarettes on behalf of adults, leading retailers to discount the importance of age verification. In contrast, e-cigarettes are newer products with no such cultural precedent, and recent targeted regulations have likely increased retailer vigilance.

We support encouraging students to wear school uniforms as a practical approach to reduce their commercial access to e-cigarettes. While it is possible that some students may attempt to circumvent this by carrying alternative clothing, doing so introduces logistic challenges and added inconvenience—such as the need to carry additional clothes, find a private place to change, or incur extra costs—which may deter such behavior.

### Limitations

There are a few limitations of this study. First, all adolescents in this study were aged 18 or 19 years and were legally permitted to purchase e-cigarettes, which may have influenced how retailers responded to them despite instructions for them to simulate underage buyers; in addition, adolescents may have behaved differently when wearing school uniforms vs casual attire, potentially affecting purchase outcomes beyond retailer perception. Second, only a 1-time purchase at each store might underestimate the actual sales, as suggested by prior studies that recommended the familiarity protocol and a test-retest approach.^26,35^ Third, each store was visited by only 1 adolescent buyer, instead of 2 in pairs,^19^ to avoid sellers’ confusion and suspicion. To minimize recall bias, we designed a 2-part instrument that allowed adequate time for step-by-step data entry.

## Conclusions

In this randomized clinical trial, we found that e-cigarettes remained widely available for purchase by adolescents without age verification in China. Wearing a school uniform appeared to reduce adolescents’ ability to purchase e-cigarettes. The findings suggest that age verification by requesting to show an ID card should be mandated for e-cigarette sellers.

## Notes

Supplement 1.
Trial Protocol

Supplement 2. **eTable 1.** Buyer, Store, and Seller Characteristics by Types of Attire for Cigarette Store Visits, China **eTable 2.** Multivariate Logistic Regressions for Successful Purchases, Inquiring About Age, Requesting ID Card, and Dissuading Use, Cigarette Store Visits, China **eTable 3.** Parallel Mediation Analysis for Requesting ID Card and Inquiring About Age **eTable 4.** Subgroup Analyses of Successful Purchases, Inquiring About Age, Requesting ID Card, and Dissuading Use Across Sex, Age, and Region **eFigure 1.** A Sampling Distribution Map of e-Cigarette Stores **eFigure 2.** Male and Female Models in School Uniforms and Casual Attire **eFigure 3.** Differences in Successful e-Cigarette Purchases, Stratified by Types of Attire and Age-Verification Behaviors **eFigure 4.** Differences in Cigarette Sales to Adolescents by Types of Attire, China **eFigure 5.** Maps of Likelihood of Successful Purchases, Inquiring About Age, Requesting ID Card, and Dissuading Use in e-Cigarette Stores, China

Supplement 3.
Data Sharing Statement
