# Supplementary material for: E-Cigarette Sales to School-Uniformed Adolescents in China: A Randomized Clinical Trial
Source: JAMA Netw Open. 2025 Oct 10;8(10):e2535623. doi: 10.1001/jamanetworkopen.2025.35623 (PMC12514632; doi:10.1001/jamanetworkopen.2025.35623)
Supplement: Supplement 1. — Trial Protocol [file jamanetwopen-e2535623-s001.pdf]

Study Protocol

A Large-Scale Randomized Control Trial to Determine the Effects of Dress by Adolescents on Electronic Cigarette Retailer Behaviors in Regulation Compliance in China

Registered ID: NCT05962411

Version Date: July 27th, 2023

Principal Investigator:

Yang Wang, Assistant Professor, China Center for Health Development Studies, Peking University, E-mail: yang.wang@hsc.pku.edu.cn

Hai Fang, Professor, China Center for Health Development Studies, Peking University, E-mail: hfang@hsc.pku.edu.cn

Funding Sources:

The China Medical Board (in the United States)

Signature Page

Authorized signatures for the protocol:

**Joint Principal Investigator**	**Signature**	**Date**
Prof Yang Wang<br>Peking University	汪洋	July 27. 2023
Prof Hai Fang<br>Peking University	方海	July 27, 2023

Study Synopsis

Lay Title	Effects of Dress by Adolescents on Electronic Cigarette Retailer Behaviors in China
Official Title	A Large-Scale Randomized Controlled Trial to Study the Effects of Adolescents' Attire on Electronic Cigarette Retailer Behaviors in Regulation Compliance in China
Registered ID	NCT05962411
Study type and phase	Non-CTIMP
Study design	a two-armed cluster randomized controlled trial
Chief investigators	Professor Yang Wang, Peking University;<br>Professor Hai Fang, Peking University
Indication under investigation	Retailers' compliance-related behavior: illegal sales of e-cigarettes to minors
Study objectives	**Primary objective**: To measure the effect of adolescents dressing in school uniforms (compared to casual attire) on regulation compliance-related behaviors of e-cigarette sellers, specifically focusing on age verification and underage cigarette sales.<br>**Secondary objectives**: (1) To determine the impact of the type of attire worn by adolescents on in-store communication between sellers and customers, specifically in terms of dissuading use. (2) To discern the differences in regulation compliance-related behaviors between e-cigarette and cigarette retailers.
Study subjects	e-cigarette stores and cigarette stores

Study groups	Intervention/Control, cluster-randomized
Study locations	36 major metropolitan areas across mainland China (including 4 municipalities directly under the Central Government, 27 provincial capitals, and 5 cities specially designated in the national plan)<br><br>4 municipalities directly under the Central Government: Beijing, Shanghai, Tianjin, Chongqing.<br><br>27 provincial capitals: Shijiazhuang (Hebei Province), Taiyuan (Shanxi Province), Hohhot (Inner Mongolia Autonomous Region), Shenyang (Liaoning Province), Changchun (Jilin Province), Harbin (Heilongjiang Province), Nanjing (Jiangsu Province), Hangzhou (Zhejiang Province), Hefei (Anhui Province), Fuzhou (Fujian Province), Nanchang (Jiangxi Province), Jinan (Shandong Province), Zhengzhou (Henan Province), Wuhan (Hubei Province), Changsha (Hunan Province), Guangzhou (Guangdong Province), Nanning (Guangxi Zhuang Autonomous Region), Haikou (Hainan Province), Chengdu (Sichuan Province), Guiyang (Guizhou Province), Kunming (Yunnan Province), Lhasa (Tibet Autonomous Region), Xi'an (Shaanxi Province), Lanzhou (Gansu Province), Xining (Qinghai Province), Yinchuan (Ningxia Hui Autonomous Region), Urumqi (Xinjiang Uygur Autonomous Region).<br><br>5 cities specially designated in the national plan: Dalian (Liaoning Province), Qingdao (Shandong Province), Ningbo (Zhejiang Province), Xiamen (Fujian Province), Shenzhen (Guangdong Province).

Eligibility criteria (inclusion)	● Identified from the AutoNavi Map through keyword searches<br>● Have reachable contact information<br>● Only one will be randomly included if multiple stores have the same contact information listed<br>● Claim to be open for business on a regular basis when contacted
Eligibility criteria (exclusion)	● Not located in main districts of the metropolitan areas.<br>● Closed when arriving or located in venues that are inaccessible to the public, such as office buildings that require membership access<br>● Cannot be located based on the AutoNavi map information<br>● Simulated buyers perceived potential danger at the surroundings
Target number of participants	1080 e-cigarette stores nation-wide
Interventions (treatment and control groups)	Each e-cigarette store will be visited by a simulated buyer (also serving as a data collector) who is 18-19 years old, wears a specific type of attire (including carried accessories), and attempts to buy e-cigarette products. If necessary, the simulated buyer will claim that (1) he or she is an adult but will not show any age proof; (2) he or she has never tried any tobacco products; and (3) he or she wants to buy e-cigarettes out of curiosity because his or her friends are using the products.<br>The sellers' behaviors and claims will be documented immediately after the simulated buyer leaves the e-cigarette store. The primary difference between the treatment group and control group is the attire that the simulated buyer wears:

	school uniforms vs. casual attire.<br><br>**Treatment Group:** The e-cigarette stores will receive a simulated buyer of randomly assigned sex who will wear a school uniform, carry a school bag, and attempt to purchase e-cigarette products. The seller in the e-cigarette stores will not be aware of this study and will assume that the simulated buyer is a real buyer of e-cigarette products.<br><br>**Control Group:** The e-cigarette stores will receive a simulated buyer of randomly assigned sex who will wear casual attire, carry a business-style bag (backpacks for males and handbags for females), and attempt to purchase e-cigarette products. The seller in the e-cigarette stores will not be aware of this study and will assume that the simulated buyer is a real buyer of e-cigarette products.<br><br>**1:1 Matching:** After mystery shopping and point-of-sale auditing in an e-cigarette store, adolescent simulated buyers will search for the nearest store where traditional cigarettes can be purchased and conduct a similar visit intervention. The seller in the cigarette stores will not be aware of this study and will assume that the simulated buyer is a real buyer of cigarette products.
Outcome measures	**The primary outcome**: The sales of e-cigarettes to the simulated buyers (school uniforms vs. casual attire), measured by the incidence of adolescent buyers successfully purchasing e-cigarette products from the stores without showing any age proof.
	**Secondary outcomes** include:<br>● Sellers verbally inquiring about the buyer's age<br>● Sellers requesting ID cards from buyers for age verification<br>● Sellers dissuading buyers from using e-cigarettes

	**Other Outcome Measures** include:<br>● Illegal flavored products being sold<br>● Online contact<br>● Delivery service offered<br>● Marketing claims
Statistical analysis plan	Summary statistics will be presented for both the treatment and control groups. The significance level for comparisons between the groups is set at α=0.05 (two-sided). A p-value of less than 0.05 will be considered statistically significant. All analyses will be performed using Stata MP 17.<br>For primary and secondary outcomes, Chi-square tests will be used to determine if there are statistically significant differences between the treatment group and control group. Multivariate logistic regression models will be further employed to analyze the treatment effect, adjusting for covariates.<br>National maps depicting the geographic variations in the outcomes will be created using the R package DrawChinaMap.
Sources of funding	The China Medical Board in the United States
Start date	July 28, 2023
Anticipated finished date	September, 2023

Table of contents

Study Synopsis 3

Table of contents 8

Study Glossary 11

1. Introduction 12

    1.1. Background 12

    1.2. Rationale and justification for the study 13

2. Hypothesis and Objectives 14

    2.1. Hypothesis 14

    2.2. Primary objective 14

    2.3. Secondary objectives 14

    2.4. Exploratory objectives 14

3. Study Design 16

4. Study subjects 18

    4.1. Study setting and locations 18

    4.2. Number of subjects to be enrolled 18

    4.3. Eligibility criteria 19

        4.3.1. Inclusion criteria 20

        4.3.2. Exclusion criteria 20

    4.4. Subject replacement 21

    4.5. Subjects for comparison 21

5. Study treatments 22

    5.1. Treatment and control 22

    5.2. Treatment procedures 22

    5.3. Design and selection of the uniforms 23

6. Outcome Measures 25

    6.1. Primary outcome measure 25

    6.2. Secondary outcome measures 25

    6.3. Other outcome measures 25

6.4. Design of the assessing tool 25
7. Study procedures 27
7.1. Subject enrollment and randomization 27
7.1.1. Screening and enrollment 27
7.1.2. Informed consent 27
7.1.3. Randomization 28
7.1.4. Blinding 28
7.2. Pilot study 29
7.3. Data collector recruitment 29
7.4. Training data collectors 30
7.4.1. Course Training 30
7.4.2. Field training 31
7.5. Randomization 31
7.6. Study visits and treatment procedures 31
8. Statistical Analyses 34
8.1. General statistical methods 34
8.2. Analysis of primary outcome 34
8.3. Analysis of secondary outcomes 34
8.4. Analysis of other outcomes 35
8.5. Subgroup analysis 35
8.6. Sample size considerations 35
8.7. Missing data 36
9. Quality control 37
9.1. Design phase of the protocol 37
9.2. Implementation phase of the protocol 37
9.3. Analysis phase of the protocol 37
10. Ethics 38
10.1. Ethical approval and informed consent 38
10.2. Legality 38
10.3. Follow-up processing 38

11. Data processing and storage 39
12. Study Timeline 40
13. Role of funding source 41
14. Declaration of interests 42
15. Reference 43
16. Appendix 46
Appendix 1 Specific Administrative Districts Included in 36 Major Metropolitan Areas of Mainland China 46
Appendix 2 Communication script in the e-cigarette store 49
Appendix 3 Pictures for models wearing school uniforms and casual attire 52
Appendix 4 Assessing tool 56
Appendix 5 Telephone protocol 74
Appendix 6 Recruitment poster for simulated buyers 77
Appendix 7 Interview process for simulated buyers 79
Appendix 8 Survey sequence of the metropolitan areas for three teams 80
Appendix 9 Code for web crawling 81

Study Glossary

Abbreviation or Term	Definition/Explanation
E-cigarette	Electronic cigarette
RCT	Randomized controlled trial
ID	Identification card

1. Introduction

1.1. Background

Electronic cigarettes (e-cigarettes) are battery-powered devices that heat a reservoir of liquid that commonly contains nicotine, flavorings and other chemicals, which are vaporized and inhaled by the users. The use of e-cigarettes in young generations has climbed significantly over the past decade globally. In the United States, the prevalence of current e-cigarette use increased from 1.5% to 19.6% among high school students during 2011-2020.[1,2] A meta-analysis of data from 69 countries and territories, published in 2021, showed that the pooled prevalence of ever and current e-cigarette use in those younger than 20 years old reached 17.2% and 7.8%, respectively.[3]

Laboratory and epidemiological studies have both suggested that e-cigarettes pose harms to respiratory and cardiovascular systems,[4,5] though the long-term health impacts remain unclear. Moreover, previous longitudinal studies showed that e-cigarette use among adolescents may act as a gateway to combustible cigarette and other substance use.[6] These evidence have raised major concerns about adolescent e-cigarette use and encouraged policymakers to introduce strict regulations, including the minimum age restriction of e-cigarette sales, in many countries.

Until 2023, over 50 countries/jurisdictions have implemented the age restriction for e-cigarette sales. However, a few studies have uncovered that such policy interventions failed to significantly reduce adolescent e-cigarette use prevalences.[7-9] One important explanation is that e-cigarette sellers were not fully compliant with the regulation.[10] They sell e-cigarettes to customers who are clearly underage without any age checks. Furthermore, minors may avoid possible inspections by disguising themselves as adults.[11] Therefore, advancing the understanding of seller compliance behaviors with age restriction and identifying associated factors are critical to the market regulation.

In China, the prevalence of current e-cigarette use among adolescents increased from 2.7% in 2019 to 3.6% in 2021, and 16.1% of middle school students had experimented with the products.[12] The Chinese Minors Protection Law prohibits the sales of cigarettes to minors under 18 years old, verified through ID checks when necessary, which has also applied to e-cigarettes since 2018.[13] Followed by this, the Chinese State Tobacco

Monopoly Administration and the Chinese State Administration for Market Regulation have launched multiple national campaigns and conducted specific inspections of points of sales and advertisements to enforce the national laws.[13] Despite lack of investigations regarding e-cigarette sales to minors in China, a prior study has shown that sellers' compliance with posting 'no sales to minors' signs is still low,[14] suggesting the illegal practice of selling e-cigarettes to adolescents may be widespread.

The practice of requiring students enrolled in school to wear a uniform varies globally, with diverse practices across the US and European Union, and widespread adoption in the United Kingdom and in Asian countries such as China, Japan, and South Korea.[15] Since 1993, some Chinese middle schools have been requiring students to wear school uniforms while attending classes, which has later become the consensus.[16] This renders it convenient to determine that a person is a middle or high school student based on the wearing of a school uniform, and thus must be an adolescent. While some studies have reported negative impacts on physical and psychological health,[17] others have suggested that school uniforms can lead to better discipline, academic performance, and class involvement.[18,19] Few research studies the effect of school uniforms on reducing illegal sales to minors.

1.2. Rationale and justification for the study

In the absence of strict regulatory enforcement, e-cigarette sellers make decisions on whether they should sell the products to customers mostly based on their judgment on the youthfulness of the customer's appearance. As a result, sellers tend to sell products to adolescents who look mature or dress as adults without checking their IDs. Even worse, some unscrupulous sellers dare to sell e-cigarettes to minors, even if they know that it is illegal. However, by allowing sellers to identify school aged adolescents more easily, uniforms may reduce illicit sales of tobacco and nicotine products. Current knowledge on the regulation compliance of e-cigarette retailers in China remains sparse. It is also unclear regarding the effects of adolescents dressing school uniforms (compared to casual attire) on the sellers' compliance behaviors.

2. Hypothesis and Objectives

2.1. Hypothesis

We hypothesize that dressing in school uniforms (compared to casual attire)* by adolescents will significantly increase the likelihood of e-cigarette sellers verifying the age of buyers in stores and reduce the probability of their illegal sales to minors.

The corresponding null hypothesis that will be tested is that dressing by school uniforms by adolescents will have no effect upon e-cigarette selling behaviors in regulation compliance.

2.2. Primary objective

To measure the effect of adolescents dressing in school uniforms (compared to casual attire) on regulation compliance-related behaviors of e-cigarette sellers, specifically focusing on age verification and underage cigarette sales.

2.3. Secondary objectives

- To determine the impact of the type of attire worn by adolescents on in-store communication between sellers and customers, specifically in terms of dissuading use.
- To discern the differences in regulation compliance-related behaviors between e-cigarette and cigarette retailers.

2.4. Exploratory objectives

- To describe the regulatory compliance of e-cigarette and cigarette sellers across mainland China.
- To document the marketing and communication strategies adopted by e-cigarette and cigarette sellers, e.g. store location, advertisements, decorations, and online

* The setting of the intervention and control in the protocol and manuscript was reversed from the time of registration, which aimed to provide readers with a better understanding of the impact of adolescents' clothing (appearance) on retailers' compliance and highlighted the protective effect of school uniforms. This would actually not influence the implementation or results of trial.

delivery service, across mainland China.

3. Study Design

This study is a two-armed, national-level, open-label cluster RCT conducted among e-cigarette stores located in 36 major metropolitan areas (including 4 municipalities directly under the Central Government, 27 provincial capitals, and 5 cities specially designated in the national plan) across mainland China. This RCT will adopt the mystery shopping approach to measure the effect of adolescent buyers' type of attire (school uniforms vs. casual attire) on the likelihood of sellers' compliance of the minimum age restriction of e-cigarette sales, defined by both age verification and sales. The e-cigarette stores in clusters will be randomly assigned to the intervention group (school uniform) or control group (casual attire) at the ratio of 1:1. Each store in the intervention group will receive a mystery buyer dressed in a school uniform, while each store in the control group will receive a mystery buyer dressed in casual attire. The adolescent buyers will attempt to purchase products and record the behaviors and marketing claims of the sellers, as well as the internal and external characteristics of the store.

Eligible stores as study subjects will be identified from the AutoNavi Map through keyword searches. Stores are eligible for enrollment if they have reachable contact information and claim to be open for business on a regular basis when contacted. The expected number of stores sampled in each metropolitan area is approximately 32, if the number allows, for a minimum expected total enrolment of approximately 1080 sellers nationwide. In addition, we plan to conduct the same trial for cigarette stores and perform a 1:1 match with e-cigarette stores. This will allow us to compare sellers' behaviors between the two attire groups in both cigarette and e-cigarette stores.

The overall study design is described by a flow chart as follows (Figure 1):

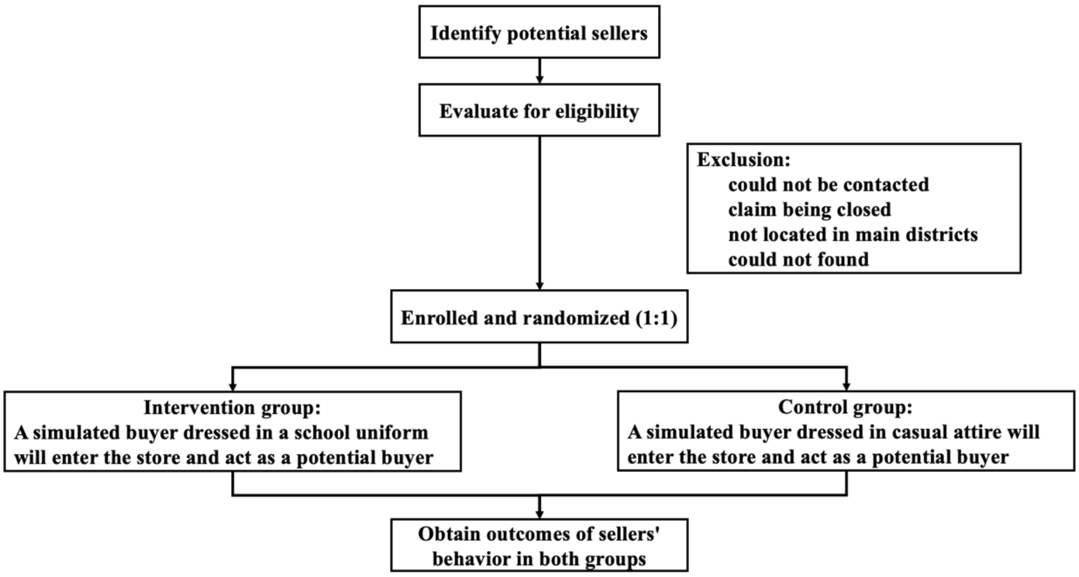

Figure 1 Flow Chart of Study Design

4. Study subjects

4.1. Study setting and locations

This study will be conducted across 36 metropolitan areas in mainland China (including 4 municipalities directly under the Central Government, 27 provincial capitals, and 5 cities specially designated in the national plan). All major metropolitan areas in mainland China are included as listed below:

4 municipalities directly under the Central Government: Beijing, Shanghai, Tianjin, Chongqing.

27 provincial capitals: Shijiazhuang (Hebei Province), Taiyuan (Shanxi Province), Hohhot (Inner Mongolia Autonomous Region), Shenyang (Liaoning Province), Changchun (Jilin Province), Harbin (Heilongjiang Province), Nanjing (Jiangsu Province), Hangzhou (Zhejiang Province), Hefei (Anhui Province), Fuzhou (Fujian Province), Nanchang (Jiangxi Province), Jinan (Shandong Province), Zhengzhou (Henan Province), Wuhan (Hubei Province), Changsha (Hunan Province), Guangzhou (Guangdong Province), Nanning (Guangxi Zhuang Autonomous Region), Haikou (Hainan Province), Chengdu (Sichuan Province), Guiyang (Guizhou Province), Kunming (Yunnan Province), Lhasa (Tibet Autonomous Region), Xi'an (Shaanxi Province), Lanzhou (Gansu Province), Xining (Qinghai Province), Yinchuan (Ningxia Hui Autonomous Region), Urumqi (Xinjiang Uygur Autonomous Region).

5 cities specially designated in the national plan: Dalian (Liaoning Province), Qingdao (Shandong Province), Ningbo (Zhejiang Province), Xiamen (Fujian Province), Shenzhen (Guangdong Province).

4.2. Number of subjects to be enrolled

The expected number of study subjects sampled from each metropolitan area is approximately 32 e-cigarette stores, if the number allows, for a minimum expected total enrollment of approximately 1,080 e-cigarette stores. Some metropolitan areas may have fewer than 32 e-cigarette stores, so more e-cigarette stores will be included from other metropolitan areas to reach the study's total.

4.3. Eligibility criteria

Due to lack of officially-released information of licensed e-cigarette stores, the list of brick-and-mortar stores and their detailed information (including address and phone number) will be compiled from the most popular Chinese navigation tool AutoNavi Map through keywords searching using the application programming interface service.

Search keywords include general terms, such as e-cigarette and vape, as well as popular brands. The keywords were determined based on the results of searches for “电子烟店” (electronic cigarette store in Mandarin Chinese) in Beijing through AutoNavi application on June 27, 2023, and we took the first 200 records as the reference for deciding the keywords. Top brands of e-cigarettes were identified according to both the initial search results and the annual production scale of e-cigarette companies in China in 2022.[20] The search terms used will be in Mandarin Chinese, and the English names of these brands will also be included (see Table 1).

The detailed information to be compiled should include store ID, store name, address, longitude and latitude, city, specific district or area, and telephone number.

Table 1 Search terms

Mandarin (used)	"电子烟店", "电子烟零售店", "电子烟体验店", "电子烟集合店", "电子烟专卖店", "电子烟直营店", "雾化", "蒸汽烟", "vape", "电子烟旗舰店", "悦刻", "魔笛", "雪加", "小野", "铂德", "柚子电子烟", "徕米", "MR 迷睿", "VTV", "福禄 FLOW", "RELX", "MOTI", "YOOZ", "snowplus", "vvild", "Boulder", "LAMI"
English translation	"e-cigarette store", "e-cigarette retail store", "e-cigarette experience store", "e-cigarette collection store", "e-cigarette exclusive store", "e-cigarette directly-operated store”, “vaporized", "vapor cigarettes", "vape", "e-cigarettes flagship store", "Yueke", "Modi", "Xvejia", "Xiaoye", "Bode", "Yozi e-cigarettes", "Lami", "MR Mirui", "VTV". "Fulu Flow", "RELX", "MOTI", "YOOZ", "snowplus", "vvild", "Boulder",

	"LAMI"

4.3.1. Inclusion criteria

The e-cigarette stores will be eligible to be sampled in this study if they meet all of the inclusion criteria:

- Identified from the AutoNavi Map through keyword searches
- Have reachable contact information
- Only one will be randomly included if multiple stores have the same contact information listed
- Claim to be open for business on a regular basis when contacted (pre-selected stores will be called ahead of investigation to inquire about their business status)

4.3.2. Exclusion criteria

Stores will not be eligible for the study if they meet one of the exclusion criteria:

- Not located in main districts of the metropolitan areas. Given the fact that most e-cigarette stores in China are located in economically developed areas with high population density,[21] this trial will only include stores in these metropolitan areas. The definition of main districts for each metropolitan area can be seen in Appendix 1.
- Closed when arriving or located in venues that are inaccessible to the public, such as office buildings that require membership access.
- Cannot be located based on the AutoNavi map information.
- Data collectors (simulated buyers) perceived potential danger at the surroundings. This usually means that the store is located in a long alley with no pedestrians in the neighborhood. Training will be given to the simulated buyers prior to the formal survey to facilitate their judgement, but whether or not to exclude trial subjects will require an instant online discussion between the simulated buyers and the study coordinators via an App in their cell phones.

4.4. Subject replacement

Given that whether an e-cigarette store will be ultimately included and audited in this study cannot be determined until simulated buyers arrive at it, we will randomly select 40 e-cigarette stores in total (8 for replacement if needed), rather than 32, for each metropolitan area in case that the final number of the subjects cannot reach the minimum sample size required for data analysis due to the exclusion criteria.

Simulated buyers with school uniforms and casual attire will be randomly assigned to buy e-cigarettes in 32 e-cigarette stores for each metropolitan area. When an e-cigarette store is excluded from the trial because it is not in business or cannot be found, the simulated buyer will go to visit the nearest store from the replacement list with the study coordinator's permission.

If too many stores are excluded for failing to meet the criteria in a metropolitan area, the simulated buyers will report the excluded ones to the study coordinator and search for the nearest eligible stores using the AutoNavi APP. The study coordinator will also provide assistance in this process, including making phone calls to confirm that the alternative e-cigarette stores are open and ensuring that they are not the ones that have already been audited.

4.5. Subjects for comparison

This study will include cigarette stores for comparisons with e-cigarette stores. For each e-cigarette store, one cigarette shop nearby will be conveniently selected for matching, because cigarettes are widely available in convenience stores, supermarkets, and tobacco and liquor shops, and thus these stores are impossible to systematically be pre-identified.

5. Study treatments

5.1. Treatment and control

Each e-cigarette store will be visited by a simulated buyer (also serving the role of a data collector) who aged 18-19 years old and wear a certain type of attire, including carried accessories. If necessary, the buyers will claim that (1) they are adults but will not show any age proof; (2) they have never tried any tobacco products; (3) they want to buy e-cigarettes out of curiosity because their friends are using the products.

The sellers' behaviors and claims will be documented immediately after the simulated buyers leave the stores. The primary difference between the treatment group and control group is the attire that buyers wear: school uniforms vs. casual attire.

Treatment group: The e-cigarette stores will receive a simulated buyer of a randomly assigned sex who will wear a school uniform, carry a school bag, and attempt to purchase e-cigarette products.

Control group: The e-cigarette stores will receive a simulated buyer of a randomly assigned sex who will wear casual attire, carry a business style bag (backpacks for males and handbags for females), and attempt to purchase e-cigarette products.

1:1 matching: After mystery shopping and point-of-sale auditing in an e-cigarette store, simulated buyers will search for the nearest store where traditional cigarettes can be purchased and conduct a similar visit intervention.

Note: The simulated adolescent buyer (also the data collector) will role-play a minor under 18 years old who attempt to buy e-cigarettes. Only adolescents aged 18 or 19 years old will be recruited in this study because it is theoretically difficult to distinguish whether they are under or above 18 years old from the facial features. This trial will not recruit minors who are younger than 18 years old because it is illegal for them to purchase e-cigarette products in China.

5.2. Treatment procedures

Before entering a store, the simulated buyers will observe and record the location and

exterior advertising from a hidden corner.

After that, the simulated buyers will enter the store and attempt to buy e-cigarette products. If the seller asks about their age, they will tell the seller that they are adults (18 years old or above) but will not show their ID cards or give any proofs to mimic the real-world situation. If the seller asks about buyer's smoking status, the buyer will claim that he/she has never tried any tobacco products including both e-cigarettes and cigarettes, and wants to buy e-cigarettes out of curiosity because his/her friends are using these products. The buyer will ultimately attempt to buy the cheapest e-cigarette product in the store for the budget reason, but he or she will not reveal this before asking about the price. To reduce potential biases caused by adolescent buyers' personalities and communication skills, a soft script will be developed to standardize the in-store interactions as much as possible (see Appendix 2).

After the assessment (approximately 10-15 minutes), simulated buyers will immediately leave the store and complete the rest of data entry to optimize retention. They will note the seller's responses during the visit, including whether or not inquiring about age, checking ID card, dissuading uses, and ultimately selling products. In addition, they will document the seller's demographic characteristics, marketing messages, interior decorations and signs. The detailed information about outcome measures is shown in Section 6. Data will be inputted via a self-developed instrument embedded in the online survey platform called Questionnaire Star accessed by smart phones.

After leaving the e-cigarette store, simulated buyers will search for the nearest cigarette shop and conduct a similar visit intervention. They will firstly observe the store's location and exterior advertising. Then, they will enter the shop and attempt to purchase a pack of cigarettes. If cigarette seller asks, they will answer that they are adults now and have never smoked. The adolescents will similarly record the behaviors and claims of the sellers in cigarette stores. The detailed information about outcome measures is shown in Section 6.

5.3. Design and selection of the uniforms

In 2015, China's Ministry of Education, together with multiple departments including the National Standardization Committee, issued a directive titled 'Opinions on Further Strengthening the Management of School Uniforms for Primary and Secondary School

Students'.[16] The policy, although not legally mandated, encourages students to wear school uniforms on weekdays. The decision on school uniforms is jointly made by the schools and committees of students' parents. However, once established in a school, it in reality becomes mandatory for all students to adhere to. Due to the fact that wearing school uniforms by secondary school students has become a social consensus, those wearing uniforms are naturally perceived as minors in China, especially when he/she looks young.

This study chooses school uniforms and casual attire as the indicator of being potential minors and adult buyers, respectively. The school uniform chosen is the most common style of middle/high schools in China, as the study will be conducted in thirty-six major metropolitan areas across mainland China. The casual attire style chosen is the most commonly seen in China. Appendix 3 shows pictures for adolescent models with pre-selected school uniforms and casual attire.

The same styles of school uniforms and casual attire with corresponding accessories, including school bags (adolescents), backpacks (male adults), and handbags (female adults), will be ordered and provided to simulated buyers free of charge.

6. Outcome Measures

6.1. Primary outcome measure

The primary outcome is the sales of e-cigarettes to simulated buyers, as adolescents successfully purchase e-cigarette products from the stores without showing any age proof.

6.2. Secondary outcome measures

Secondary outcomes include:

- Sellers' verbally inquiring about buyer age
- Sellers' requesting ID cards from buyers for age verification
- Sellers' dissuading buyers from using e-cigarettes (added after the pilot study)

6.3. Other outcome measures

Other outcome measures primarily include:

- Illegal flavored products being sold

  The simulated buyers will report if sellers mention that flavored (not allowed in China) e-cigarette products are available in the stores.

- Online contact

  The simulated buyers will report if WeChat account information is publicly displayed for adding contacts.

- Delivery service offered

  The simulated buyers will report if sellers mention that products ordered online can be delivered to them.

- Marketing claims

  The simulated buyers will record the marketing claims of sellers to promote the products

6.4. Design of the assessing tool

The assessing tool will be used by simulated buyers to check whether all information is

recorded after leaving the store, and all data will be inputted.

The assessing tool is designed primarily based on the Vape Shops Standardized Tobacco Assessment for Retail Setting (V-STARS)[22] and Tobacco Advertising, Promotion and Sponsorship (TAPS) bans compliance guide[23], and published articles[14]. It will be further adapted by investigators who are familiar with Chinese e-cigarette retailers and laws and have observed store characteristics, to the Chinese context to collect information mainly on store internal/external environmental features (e.g., location, advertising, warning signs, and licensing), product availability, and seller behaviors.

After the initial version of the assessing tool is finished, a pilot investigation will be conducted to further revise and finalize this instrument. The pilot trial will be conducted by two simulated buyers (18-19 years old, see the requirements in Section 7.3), accompanied with coordinators, in 2 metropolitan areas: Beijing and Shijiazhuang (the capital city of Hebei Province). Each simulated buyer will conduct mystery shopping and point-of-sale auditing in 10 e-cigarette stores. After data collection is complete for each store, coordinators will discuss with simulated buyers encountered problems and possible solutions, as well as further refine the research process and assessing tool. Detailed information can be seen in Section 7.2. The final assessing tool can be seen in Appendix 4.

7. Study procedures

The trial will be registered at ClinicalTrials.gov, and will be conducted after getting permission or exemption from the ethical review from Peking University Institutional Review Board (IRB).

7.1. Subject enrollment and randomization

7.1.1. Screening and enrollment

First, after obtaining a list of e-cigarette stores through keyword searching, we will remove e-cigarette stores without contact information based on the eligibility criteria. Stores not located in main districts of the metropolitan areas (see Appendix 2) will be then removed with Excel filter function, and investigators will further manually delete those that are judged to be non-e-cigarette shops. In this way, a pool for the sampling of trial subjects will be generated.

Second, using R software, each store will be assigned a random number and all the stores in the list will be ordered by the random number from smallest to largest, so that each store with a phone number will have an equal probability of being enrolled in this study. Subsequently, as each selected store needs to be contacted for confirming their opening, this trial will further exclude those stores which have the same contact information as the other store. If more than one store with the same telephone number exists, only the first one will be kept.

Finally, e-cigarette retailers will be called in the ascending order of random numbers for inquiring about their operating status and verifying that their address is correct, until 40 stores are confirmed to be still in business for each metropolitan area (if the number allows). A telephone protocol (Appendix 5) will be used to standardize communication process to collect relevant information. Callers will pretend to be potential buyers who may be visiting the store shortly, and each store will be contacted once again on the second day if not answered.

7.1.2. Informed consent

This study uses the mystery shopping approach and all the e-cigarette store owners and sellers will not be informed that they are enrolled in this trial.[24-26]

7.1.3. Randomization

Prior to the randomization of subjects, all the eligibility criteria must be met.

- **Generating clusters to which the stores belong**: Considering traveling time and the efficiency of data collection, this trial will use a cluster randomization approach, rather than simple randomization. Investigators will firstly map all the 40 selected stores within each metropolitan area, then divide them into clusters that contains the same number (usually 4 stores) considering their locations and public transportation. Then each cluster will be digitally labeled, from area 1 to area 8. The labeling of the clusters follows the principle of from top to bottom, from left to right on the map.

- **Assigning clusters to treatment**: Each cluster will be randomly assigned to one simulated buyer, and the attire (school uniforms or casual attire) he/she wears on a specific day will also be random. In this way, the e-cigarette stores will finally be allocated at a 1:1 ratio to either the intervention or control group.

  (1) Specifically, the simulated buyers (data collectors) to be recruited are 18 to 19 years old, with a 1:1 ratio of males to females. To reduce bias associated with personal characteristics of simulated buyers, they will be randomly assigned a new digital number for each metropolitan area, which is used for matching the clusters.

  (2) The type of attire wore by each adolescent on a specific day will be pre-determined using the Excel's random number generator within each sex, and each adolescent will have an equal probability to wear school uniforms and casual attire during the entire study.

7.1.4. Blinding

It will be impossible to blind study subjects regarding the assignment to the intervention or control group, as letting sellers in the e-cigarette stores see the buyers' attire is part of the treatment. However, sellers will not be aware of the participation in this trial because of the mystery shopping approach adopted.

The trial team, consisting of both data collectors and coordinators collecting outcomes, will also not be blind to e-cigarette stores in the intervention and control groups, because there is no way to not let them know the e-cigarette stores with simulated buyers with school uniforms or casual attire on each day.

The statistician conducting analyses will have no direct contact with e-cigarette stores but will not be blind to the treatment allocation.

7.2. Pilot study

Aim: To refine the treatment procedures and improve the assessing tools

Subjects: 20 e-cigarette stores, including 10 in Beijing and 10 in Shijiazhuang (the capital city in Hebei Province). These 20 e-cigarette stores will be selected from the list we complied, and will not be revisited in the formal trial.

Time: After subject enrollment but prior to data collector training

Data collectors (simulated buyers): two adolescents aged 18 or 19 years old, including one male college student and one female college student. The requirements for them are same as the that for the formal trial. For each adolescent, a coordinator will accompany him/her for any necessary help and further discussion for the treatment procedures.

Treatment: 10 stores will receive an adolescent buyer wearing casual attire and the other 10 stores will receive a simulated buyer wearing school uniforms. All the treatment procedures will be same as the formal trial, which can be seen in **Section 5**.

Evaluation after the pilot trial: After the data collection of 20 polit e-cigarette stores, the research team will discuss the problems encountered and possible solutions with each simulated buyer, as well as further refine the research process and the assessing tool.

7.3. Data collector recruitment

We plan to recruit 25 college students in Beijing through social media in June-July 2023, including 12 males and 13 females, where the one additional female data collector will be recruited considering that females may need to take some days off during the fieldwork due to health-related reasons. This way, a gender-balanced sample will be more likely to be achieved.

Requirements including:

- Aged 18 or 19 years old (according to the WHO standard, adolescents are defined to less than 20 years old)
- Having never used any tobacco products including e-cigarettes and cigarettes

- No apparent dyed hair
- No tattoos being seen

A poster will be designed and posted in WeChat Moments and online forums to recruit the simulated buyers in Beijing. It also will be shared among faculty, staff, and students at several universities in Beijing. The poster will contain the following information, including study title, location, time of the investigation, requirements, compensation, and contact information. Considering the need to keep the study confidential from Chinese e-cigarette retailers, the investigators will avoid mentioning too many details in the recruitment poster and only inform adolescents of more specific information in the subsequent interview sessions.

The coordinator will create a WeChat group of the adolescents who sign up. In July 2023, an online interview will be performed to communicate with those who are willing to take part in, providing them with more details about the trial, and check whether they are eligible for the investigation.

Each simulated buyer will receive CNY 350 (approximately USD 50) per day as payment and all accommodation and transportation costs will be covered by the study.

7.4. Training data collectors

7.4.1. Course Training

After recruiting the adolescents participating in the trial, the investigators will arrange a one-day on-site training. The training will be conducted in the form of PowerPoint presentations and lectures by investigators and coordinators. The training date is set within seven days prior to the formal investigation.

The training content will include the study background, purpose, process, time schedule, precautions in the fieldwork, etc. Investigators will make sure that simulated buyers understand the roles they play. Coordinators will show them the pictures of retail stores, product advertisements, and warning signs to help them quickly and accurately identify the details they need to record in the study. At the end of the lecture, the coordinator will role-play the seller in one (or more) simulated interactions with simulated buyers to help them better perform in real-life possible scenarios.

An investigation manual will be provided by the coordinator to the simulated buyers to guide them in the proper conduction of the survey and completion of the questionnaire. The manual will include training contents, detailed explanations of the questionnaire items, special situations handling strategies, and how to communicate with sellers in a standardized manner (without making them suspicious).

7.4.2. Field training

On the second training day, simulated buyers will practice fieldwork at nearby e-cigarette stores with coordinators accompanying them. The goal of the field training is to further enhance the simulated buyers' understanding of the process, proficiency in memorizing details, and ability to complete the questionnaire

The practice in the field will be conducted by a single simulated buyer, and the process will be exactly the same as the formal trial. Sellers in the e-cigarette stores will not be aware that they are being studied, and the physical environment will be recorded by the coordinators beforehand. During the field training, the coordinator will wait outside e-cigarette stores and discuss the difficulties and problems encountered after the survey is completed, to help them effectively develop skills for data collection. Each simulated buyer will try several stores until they are familiar with the entire process and get more than 90% of the objective items in the questionnaire correct. Otherwise, they will be taken to the next e-cigarette store to continue their field practice. The entire training will last 3-5 days.

7.5. Randomization

Each simulated buyer will be assigned with a random number and the clusters of e-cigarette stores will be randomly allocated to the intervention group or the control group before the visits. Details on randomization can be found in **Section 7.1.3.** Randomization and assignment of all simulated buyers and e-cigarette stores will be completed before the start of the formal survey.

7.6. Study visits and treatment procedures

The formal study will begin in July 2023. The simulated buyers and coordinators will be

divided into three teams, each including eight simulated buyers[†] (18-19 years old) and two coordinators (graduate students). Each team will travel from Beijing to all the metropolitan areas across mainland China (Appendix 8).

Simulated buyers will travel to new metropolitan areas by train or air and change into the prepared attire at the hotel (the type of attire worn on that day will be pre-determined by a random number). The team coordinator will then assign a list of pre-specified e-cigarette stores to each simulated buyer. Using the assistance of the AutoNavi Map, simulated buyers will travel by public transportation according to the geographic information on the list to the e-cigarette stores to conduct the survey. Each simulated buyer will visit 4-5 e-cigarette stores per day if possible.

The simulated buyers will take on the role of an adolescent who attempt to buy e-cigarette products, go into a store, talk to the seller, and try to make the purchase. They will record all the information using Questionnaire Star (an App) on their smart phone after the visit is completed. Upon the arrival, the simulated buyers will first observe the external environment of the store out of sight of the seller and fill in the related information. They will then enter the store, purchase e-cigarette products, and record seller responses and the internal environment of the store. After memorizing the information, they will leave the store, complete, and submit the data collection form online. The same simulated buyer will conduct a similar trial in one of the nearest cigarette stores, which serves as a match to the e-cigarette store that he or she just visited. After completing the visit, they will then travel to the next e-cigarette store. Survey details and measurements can be seen in **Section 5** and **Section 6**.

The coordinators will provide them with necessary help and answers to their queries during the fieldwork. If any of the included stores happen to be closed or unavailable, the coordinators will direct them to the nearest alternative e-cigarette store from the replacement list. If the number of inaccessible stores is too high, the coordinators will search for available e-cigarette stores nearby the simulated buyers and confirm their operating status at that time by phone, and then assign simulated buyers to go to survey them when possible.

[†] One team will include 4 male and 5 female adolescent buyers due to the inclusion of an extra female data collector.

Each team will need at least one day to complete the survey in one metropolitan area, and the survey is expected to last between 3 and 5 weeks, considering the long distances between study sites (metropolitan areas), uncertain weather conditions, delayed transportation, and illness of personnel. The survey is scheduled to be fully completed by September 2023 for the data collection in all 36 metropolitan areas across mainland China.

8. Statistical Analyses

8.1. General statistical methods

Summary statistics will be presented for both the treatment and control groups. Continuous variables will be summarized by the number of observations, mean, standard deviation, median, minimum, and maximum. Categorical variables will be summarized using frequency counts and percentages. Descriptive analyses of the distributions of the e-cigarette seller behavior outcomes between two types of attire (treatment and control groups), including successful purchases, inquiring about buyer age, requesting ID cards for age verification, and dissuading buyers from use, as well as store environment features, and sellers' demographic characteristics, will be conducted with t-tests and chi-square tests detecting statistically significant differences.

The significance level for comparisons between the two groups is set at α=0.05 (two-sided). Differences between the study groups will be considered statistically significant if the p-value is less than 0.05. All analyses will be performed using Stata MP 17.

8.2. Analysis of primary outcome

For the primary outcome, successful purchase rates will be analyzed using Chi-square tests to determine if the differences between the treatment group and the control group are statistically significant. Multivariate logistic regression models will be further employed to analyze the treatment effect, adjusting for covariates.

A national map depicting the geographic variations in the outcomes of successful purchase rates will be created using the R package DrawChinaMap.

8.3. Analysis of secondary outcomes

Secondary outcomes, including inquiring about the buyer's age, requesting ID cards for age verification, and dissuading buyers from use, will be analyzed in a similar manner to the primary outcome. Chi-square tests will be used to detect the statistical significance of differences between the two study groups, and logistic regression models, adjusted for covariates and appropriate for the distribution of the outcomes, will be employed. Additionally, similar national maps describing geographic variations will be generated.

8.4. Analysis of other outcomes

Other outcomes, including illegal flavored products, online contact, and delivery service offered, will be analyzed in a similar way as the primary outcome. Chi-square tests will be used to detect statistical significance in differences between the two groups, and logistic regression models appropriate for the distribution of the binary outcomes with covariate adjustments will be employed. Similar national maps describing geographic variations will be generated as well. For marketing claims and strategies, we will further employ thematic framework analysis to qualitatively analyze and describe the marketing methods and tactics of e-cigarette stores.

8.5. Subgroup analysis

For matched e-cigarette and cigarette stores, we will employ McNemar's tests to assess the distinctions in the outcomes between e-cigarette stores and cigarette stores. Rates of successful e-cigarette purchases stratified by age checking behaviors and buyer attire will also be reported. Planned subgroup analyses are intended to explore potential effect modifications of gender, age, metropolitan area and store types.

8.6. Sample size considerations

Successful purchase rates of e-cigarette products for the school uniform group and the casual attire group will be derived from the pilot study. Other parameters required for the cluster-RCT sample size calculation are as follows:

- Randomization in a 1:1 ratio
- $\alpha = 0.05$ (two-sided)
- power = 0.85
- cluster size = 4 (both for intervention group and control group)
- inter-cluster correlation coefficient = 0.5 (default)

However, considering the primary goal of this study is to characterize e-cigarette seller behaviors at a national level, we decide to set the final sample size to be approximately 1,080, which may be far more than the minimum sample required (about 3 times) for testing the effect of buyer attire on e-cigarette sales to adolescents.

8.7. Missing data

Since simulated buyers use the instrument embedded in an online survey platform to fill out the collected information, there is no chance that they will miss any questions as the system will remind them of any potential missing values. The pilot survey will help us confirm which questions can be filled in, considering the possibility that the adolescents may be asked to leave by the sellers for not being able to show proof of age as soon as he/she enters the store. While filling in questions regarding the store external environment, simulated buyers can select "Unable to observe" and skip questions that cannot be answered. Missing data on the store internal environment due to the reason of being asked to leave will not affect our main analysis. For the in-store environmental characteristics, only subjects with available data will be included in the analyses.

9. Quality control

9.1. Design phase of the protocol

- Provide trainings to simulated buyers with a study manual and effective communication skills to ensure the quality of research in the fieldwork;
- Prior to study implementation, a pilot survey will be carried out using the designed assessing tool. Subsequently, we will modify the tool based on seller responses and preliminary results to improve its operability and accuracy of information collected.
- Data input will be completed an online self-developed assessing tool, eliminating the possibility of missing critical items.

9.2. Implementation phase of the protocol

- The inclusion criteria for study subjects will be strictly followed;
- The randomization of the e-cigarette stores will be strictly carried out using random numbers.
- Two staff members will accompany the team to each surveyed metropolitan area and provide implementation assistance to simulated buyers and answers to their questions whenever needed.

9.3. Analysis phase of the protocol

Two analysts will conduct independent data cleaning and logical error correction of data (e.g., range checks for data values). Any discrepancies uncovered will be resolved by the principal investigators.

10. Ethics

10.1. Ethical approval and informed consent

According to previous literature[24-27], this study does not involve human subject research or collect any private information. Prior to the study, the investigators will submit the protocol to the Institutional Review Board at Peking University, which may exempt this trial from ethical review.

Following prior research, this trial uses the mystery shopping approach, and all the study subjects (e-cigarette stores and cigarette stores) will not be informed that they will be enrolled in this trial.[24-26]

10.2. Legality

Purchase of e-cigarette products by adolescent buyers (age >=18) is legal in China. This study does not involve the collection of private personal information from sellers.

10.3. Follow-up processing

For the purchased e-cigarette and cigarette products, some will be given to scientific research institutions for academic purposes, and others will be sent to tobacco control associations as samples for educational purposes.

11. Data processing and storage

The data will be collected through the self-developed instrument embedded in the Questionnaire Star platform (an App on smart phones), downloaded by the investigators at the end of the survey and saved on the principal investigator's computer located in the office. Statisticians will create the database in a timely manner, and the data will then be backed up and locked with passwords by investigators and research assistants after it has been thoroughly reviewed. To ensure data security, a non-permitted person cannot modify the data. All related analyses will be conducted on the principal investigator's computer.

Staff who have access to the information of the trial subjects will sign confidentiality agreements. All the data in the study will be available to principal investigators, who have ultimate authority for the decision on data. The information shall not be disclosed to any third party (other than persons or institutions directly involved or required to know by law) without the prior consent of principal investigators. The investigators must take all appropriate precautions to avoid disclosing the information to third parties or the public by any staff or institutions.

12. Study Timeline

We plan to complete the preliminary recruitment of adolescents who will work as simulated buyers and data collectors, store sampling, and pilot survey from June 2023 to July 2023. In July 2023, we will train the simulated buyers and conduct the randomization of simulated buyers and e-cigarette stores. From July 2023 to September 2023, we will divide the data collectors into three teams and carry out the trial investigation. Data cleaning and analysis will be conducted after September 2023.

13. Role of funding source

This study is funded by the China Medical Board in the United States. The funder will have no role in the study design, data collection, analysis, interpretation, or writing of the paper.

14. Declaration of interests

There are no financial or other competing interests for the principal investigators in the overall trial or at each study site.

15. Reference

1. Tobacco product use among middle and high school students--United States, 2011 and 2012. *MMWR Morb Mortal Wkly Rep*. Nov 15 2013;62(45):893-7.
2. Gentzke AS, Wang TW, Jamal A, et al. Tobacco Product Use Among Middle and High School Students - United States, 2020. *MMWR Morb Mortal Wkly Rep*. Dec 18 2020;69(50):1881-1888. doi:10.15585/mmwr.mm6950a1
3. Yoong SL, Hall A, Leonard A, et al. Prevalence of electronic nicotine delivery systems and electronic non-nicotine delivery systems in children and adolescents: a systematic review and meta-analysis. *Lancet Public Health*. Sep 2021;6(9):e661-e673. doi:10.1016/s2468-2667(21)00106-7
4. Espinoza-Derout J, Shao XM, Lao CJ, et al. Electronic Cigarette Use and the Risk of Cardiovascular Diseases. *Front Cardiovasc Med*. 2022;9:879726. doi:10.3389/fcvm.2022.879726
5. Gotts JE, Jordt SE, McConnell R, Tarran R. What are the respiratory effects of e-cigarettes? *Bmj*. Sep 30 2019;366:l5275. doi:10.1136/bmj.l5275
6. Chan GCK, Stjepanović D, Lim C, et al. Gateway or common liability? A systematic review and meta-analysis of studies of adolescent e-cigarette use and future smoking initiation. *Addiction*. Apr 2021;116(4):743-756. doi:10.1111/add.15246
7. Ferrell A, Hadddad L, Harrison Elder J, Garvan C, Cook CL, Salloum R. Perceptions and Use of Electronic Nicotine Delivery Systems Among Floridian Middle and High School Students: Secondary Analysis of Cross-sectional Survey Results. *Tob Use Insights*. 2020;13:1179173x20953402. doi:10.1177/1179173x20953402
8. Grube JW, Lipperman-Kreda S, García-Ramírez G, Paschall MJ, Abadi MH. California's tobacco 21 minimum sales age law and adolescents' tobacco and nicotine use: differential associations among racial and ethnic groups. *Tob Control*. Jun 30 2021;doi:10.1136/tobaccocontrol-2020-056219
9. Hawkins SS, Kruzik C, O'Brien M, Levine Coley R. Flavoured tobacco product restrictions in Massachusetts associated with reductions in adolescent cigarette and e-cigarette use. *Tob Control*. Jan 27 2021;doi:10.1136/tobaccocontrol-2020-056159
10. Yan D, Wang Z, Laestadius L, et al. A systematic review for the impacts of global

approaches to regulating electronic nicotine products. *J Glob Health*. Aug 25 2023;13:04076. doi:10.7189/jogh.13.04076

11. Arria AM, Caldeira KM, Vincent KB, Bugbee BA, O'Grady KE. False identification use among college students increases the risk for alcohol use disorder: results of a longitudinal study. *Alcohol Clin Exp Res*. Mar 2014;38(3):834-43. doi:10.1111/acer.12261

12. Chinese Center for Disease Control and Prevention. Tobacco Epidemic Monitoring Results among Chinese Middle School and College Students in 2021. Xinjiang Uygur Autonomous Region Center for Disease Control and Prevention. 2024.

13. Jiang J, Zheng Z. A Critical Review of E-Cigarette Regulation in China: Challenges and Prospects for Youth Prevention and Tobacco Control. *Nicotine Tob Res*. Jan 22 2024;26(2):126-134. doi:10.1093/ntr/ntad180

14. Nian Q, Cohen JE, Cui Y, Zhang S. Tobacco retailers around schools in 10 cities across China. *Tob Control*. Jun 16 2022;doi:10.1136/tobaccocontrol-2022-057367

15. School Uniforms by Country 2024. World Population Review. April 23, 2024, 2024. Accessed April 23, 2024, 2024. https://worldpopulationreview.com/country-rankings/school-uniforms-by-country

16. China MoEotPsRo. Opinions on Further Strengthening the Management of School Uniforms for Primary and Secondary School Students.

17. Reidy J. Reviewing School Uniform through a Public Health Lens: Evidence about the Impacts of School Uniform on Education and Health. *Public Health Reviews*. 2021;42:1604212. doi:10.3389/phrs.2021.1604212

18. Baumann C, Krskova H. School discipline, school uniforms and academic performance. *International Journal of Educational Management*. 2016;30(6):1003-1029. doi:10.1108/ijem-09-2015-0118

19. Gentile E, Imberman SA. Dressed for success? The effect of school uniforms on student achievement and behavior. *Journal of Urban Economics*. 2012;71(1):1-17. doi:10.1016/j.jue.2011.10.002

20. Institute CPIR. *Report of Prospects and Investment Strategy Planning Analysis on China New Tobacco Products Industry, 2022-2027*. 2022.

21. Commerce CECo. *China's Electronic Cigarette Industry Blue Book 2021*. 2021.

22. Kong AY, Eaddy JL, Morrison SL, Asbury D, Lindell KM, Ribisl KM. Using the Vape

Shop Standardized Tobacco Assessment for Retail Settings (V-STARS) to Assess Product Availability, Price Promotions, and Messaging in New Hampshire Vape Shop Retailers. *Tob Regul Sci*. Apr 2017;3(2):174-182. doi:10.18001/trs.3.2.5

23. Polanska K, Kaleta D. Tobacco and E-Cigarettes Point of Sale Advertising-Assessing Compliance with Tobacco Advertising, Promotion and Sponsorship Bans in Poland. *Int J Environ Res Public Health*. Feb 18 2021;18(4)doi:10.3390/ijerph18041976

24. Berg CJ, Barker DC, Meyers C, et al. Exploring the Point-of-Sale Among Vape Shops Across the United States: Audits Integrating a Mystery Shopper Approach. *Nicotine Tob Res*. Feb 16 2021;23(3):495-504. doi:10.1093/ntr/ntaa041

25. Chang J, Xu S, Zhu S, et al. Assessment of non-prescription antibiotic dispensing at community pharmacies in China with simulated clients: a mixed cross-sectional and longitudinal study. *Lancet Infect Dis*. Dec 2019;19(12):1345-1354. doi:10.1016/s1473-3099(19)30324-x

26. Hrywna M, Kong AY, Ackerman C, Hudson SV, Delnevo CD. Retailer Compliance With Tobacco 21 in New Jersey, 2019-2020. *JAMA Netw Open*. Oct 3 2022;5(10):e2235637. doi:10.1001/jamanetworkopen.2022.35637

27. Roeseler A, Vuong TD, Henriksen L, Zhang X. Assessment of Underage Sales Violations in Tobacco Stores and Vape Shops. *JAMA Pediatr*. Aug 1 2019;173(8):795-797. doi:10.1001/jamapediatrics.2019.1571

16. Appendix

Appendix 1 Specific Administrative Districts Included in 36 Major Metropolitan Areas of Mainland China

**Metropolitan area**	**Administrative districts included**
Beijing (Beijing Municipality)	Dongcheng, Xicheng, Haidian, Chaoyang, Shijingshan, Fengtai
Changchun (Jilin Province)	Nanguan, Kuancheng, Chaoyang, Luyuan, Erdao
Changsha (Hunan Province)	Furong, Tianxin, Kaifu, Yuelu, Yuhua
Chengdu (Sichuan Province)	Jinjiang, Qingyang, Wuhou, Chenghua, Jinniu
Chongqing (Chongqing Municipality)	Jiangbei, Nan'an, Yuzhong, Shapingba, Jiulongpo
Dalian (Liaoning Province)	Zhongshan, Xigang, Shahekou, Ganjingzi
Fuzhou (Fujian Province)	Cangshan, Gulou, Jin'an, Taijiang
Guangzhou (Guangdong Province)	Liwan, Yuexiu, Tianhe, Haizhu, Baiyun, Huangpu, Panyu
Guiyang (Guizhou Province)	Nanming, Yunyan, Baiyun, Guanshanhu, Huaxi, Wudang
Haikou (Hainan Province)	Xiuying, Qiongshan, Longhua, Meilan
Hangzhou (Zhejiang Province)	Shangcheng, Gongshu, Binjiang, Linping, Qiantang, Xihu
Harbin (Heilongjiang Province)	Nangang, Daoli, Daowai, Xiangfang, Pingfang, Songbei

Hefei (Anhui Province)	Shushan, Yaohai, Baohe, Luyang
Hohhot (Inner Mongolia Autonomous Region)	Xincheng, Huimin, Yuquan, Saihan
Jinan (Shandong Province)	Huaiyin, Lixia, Tianqiao, Shizhong, Licheng, Changqing
Kunming (Yunnan Province)	Wuhua, Panlong, Guandu, Xishan
Lanzhou (Gansu Province)	Chengguan, Qilihe, Anning, Xigu, Honggu
Lhasa (Xizang Autonomous Region)	Chengguan, Duilongdeqing
Nanchang (Jiangxi Province)	Donghu, Xihu, Qingyunpu, Honggutan, Xinjian, Qingshanhu
Nanjing (Jiangsu Province)	Xuanwu, Qinhuai, Jianye, Gulou, Qixia, Yuhua
Nanning (Guangxi Autonomous Region)	Xingning, Qingxiu, Xixiangtang, Jiangnan, Liangqing, Yongning
Ningbo (Zhejiang Province)	Haishu, Jiangbei, Beilun, Zhenhai, Yinzhou, Fenghua
Qingdao (Shandong Province)	Shinan, Shibei, Laoshan, Licang, Huangdao
Shanghai (Shanghai Municipality)	Huangpu, Xuhui, Changning, Jing'an, Putuo, Hongkou, Yangpu, Pudong New Area
Shenyang (Liaoning Province)	Heping, Shenhe, Dadong, Huanggu, Tiexi
Shenzhen (Guangdong Province)	Futian, Luohu, Nanshan, Yantian
Shijiazhuang (Hebei Province)	Changan, Qiaoxi, Xinhua, Yuhua

Taiyuan (Shanxi Province)	Xinghualing, Jiancaoping, Yingze, Wanbailin, Xiaodian, Jinyuan
Tianjin (Tianjin Municipality)	Heping, Hedong, Hexi, Nankai, Hebei, Hongqiao
Urumqi (Xinjiang Autonomous Region)	Tianshan, Shayibake, Xinshi
Wuhan (Hubei Province)	Jianghan, Wuchang, Jiang'an, Hongshan, Hanyang
Xiamen (Fujian Province)	Siming, Huli, Haicang, Jimei
Xi'an (Shaanxi Province)	Lianhu, Xincheng, Yanta, Beilin, Baqiao, Weiyang
Xining (Qinghai Province)	Chengdong, Chengzhong, Chengxi, Chengbei
Yinchuan (Ningxia Autonomous Region)	Xingqing, Xixia, Jinfeng
Zhengzhou (Henan Province)	Jinshui, Erqi, Guancheng Hui, Zhongyuan

Appendix 2 Communication script in the e-cigarette store

Soft script

When the simulated buyer, acting as a customer, communicates face-to-face with the seller in an e-cigarette store, the main information recorded includes: successful purchases of e-cigarette products, inquiries about age, requests to show ID cards, dissuasion from using e-cigarettes, price information, and marketing strategies (such as the store clerk adding you on WeChat). You can use the following conversational sentences to naturally gather the above information:

Other key points: advertising area, warning slogans, decorations, product types, flavor recommendations, information conveyed, and discounted prices.

Legal requirements: (record only)

- **Does the seller in the e-cigarette store ask about your age?**
- **Have you been asked by the seller to show your ID card?**

1. When you walk into the store or approach the counter, first glance at the wall to see if there is a tobacco license. Then look at the e-cigarette products, paying attention to any "No use by minors" warning signs and "Smoking or vaping is harmful to health" warning signs. Continue to pretend you are browsing the store.

2. Typically, the seller will notice you immediately and ask what you are looking for. You should respond with "I'm just browsing," "I'm here to learn about the products," or "My friend uses e-cigarettes, and I'm here to check them out."

3. The seller will likely ask if you have used e-cigarettes or smoked before. You should respond, "I have never used them before." At this point, some sellers may advise you not to buy or try them.

4. You should ask the seller, "Do you have any recommendations?" The seller will likely recommend a lighter flavor product and hand it to you. You then ask, "Is this detachable?" or "Can this be used continuously?" to determine the type of e-cigarettes sold in the store. While doing this, take the opportunity to look around to see if there are disposable e-cigarettes and open-system e-cigarettes, as well as whether protective cases and lanyards are sold at the counter.

5. During the ongoing product viewing phase, point to the various e-cigarette cartridges on the counter or shelf and ask the seller about their differences. Then further inquire, "Which ones sell well?" or "What do people often buy?" or "Which are popular?" Take note of the first and second flavors mentioned by the clerk. If the seller offers you a sample, be sure to refuse!

6. After learning about the popular flavors, express how popular e-cigarettes are around you by saying something like, "I have a few friends who use them," "I've seen many people using them," or "I've seen others using them several times while shopping." Use this to prompt the seller to explain why e-cigarettes are popular. They will generally actively market a positive image of e-cigarettes. Please note the content and order of what the seller says, taking down the first two to three points.

7. By asking "I've smelled someone using an e-cigarette with a sweet flavor, do you have that?", observe whether the seller tries to sell you flavored e-cigarettes, and note the flavor. Be careful not to mention words like "fruit" to avoid arousing the seller's suspicion. Pay attention to whether the seller mentions illegal products like "milk tea cups" and record them, but the decoy should not bring them up proactively.

8. Inquire about the prices of e-cigarettes, including the cigarette rod and cartridge. If the seller recommends Relx e-cigarettes, respond with, "That's a bit expensive, any discounts?" to record discount information. Then say, "I want to try first, do you have anything cheaper?" to express that it is still too expensive. Continue this until the seller explicitly replies that it is the cheapest product in the store. If the store only sells pod-based e-

cigarettes, say, "I can use my friend's cigarette rod" to purchase only the cartridge and not the rod. Disposable e-cigarettes are usually cheaper than pod-based ones, and if offered by the seller, you can purchase them.

9. When you pay, observe whether there are contact details (such as WeChat number, WeChat group code, phone number, etc.) on the counter. If the seller actively adds you on WeChat, they will likely also mention, "We offer online mailing services." If the seller does not actively mention online mailing, you should ask, "Why add WeChat?" and record the seller's reaction.

10. You should observe whether there are any "free trial" signs or disposable flavor filters placed in the store. Pay attention to whether the seller advises you against using e-cigarettes.

11. While you are in the store picking products or after making a purchase, take a moment to naturally browse the merchandise. Observe whether other items like tobacco, alcohol, or areca nuts are sold on the shelves, and whether toys and decorative ornaments are displayed.

Handling Special Situations:

- If a seller offers you to try an e-cigarette, they usually provide a disposable tool. Respond with, "This seems unsanitary, I won’t use it... I’d rather buy a new one/I’d rather use my friend’s."
- The seller might claim that the e-cigarette and cartridges are sold as a set, and that a friend’s e-cigarette might not be compatible. You should insist on only buying the cartridges, "It’s fine, I remember it’s just like this one/... just buy a box of these."

Appendix 3 Pictures for models wearing school uniforms and casual attire

School uniform, male

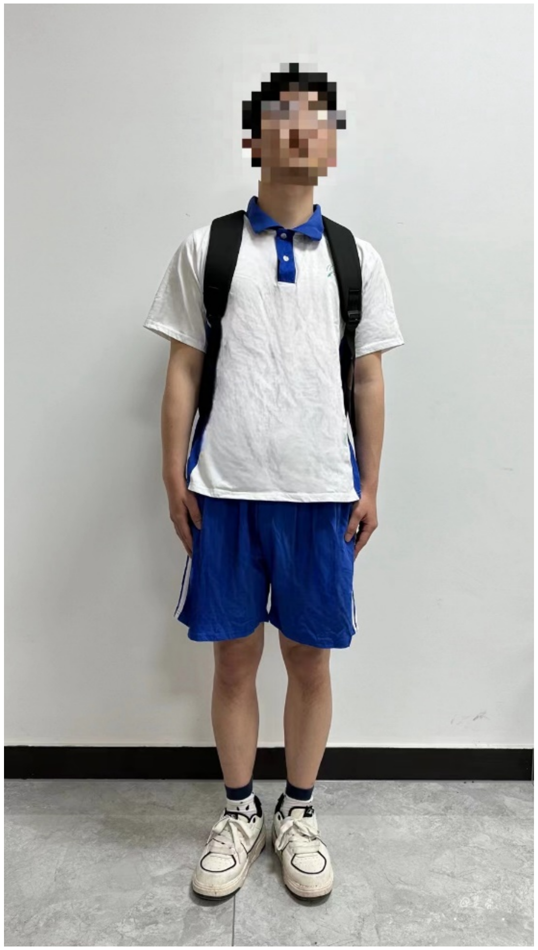

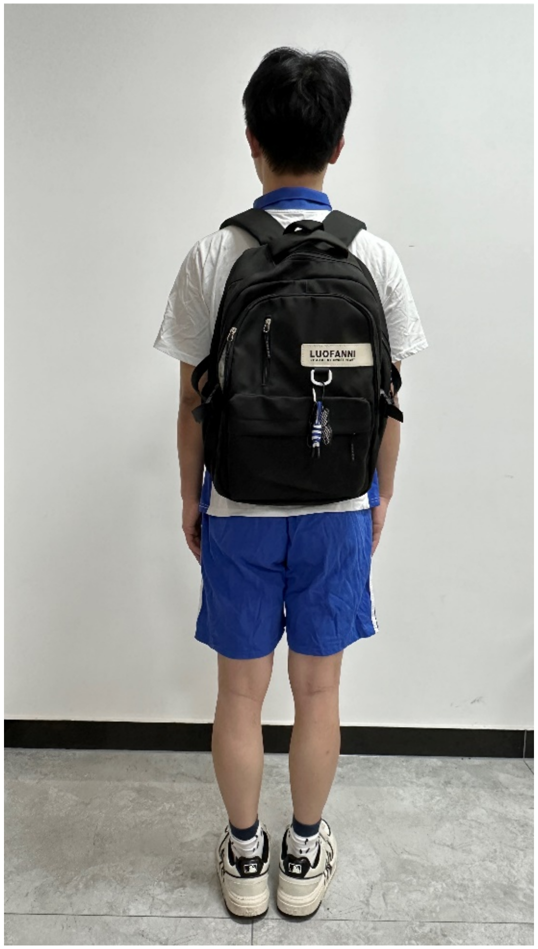

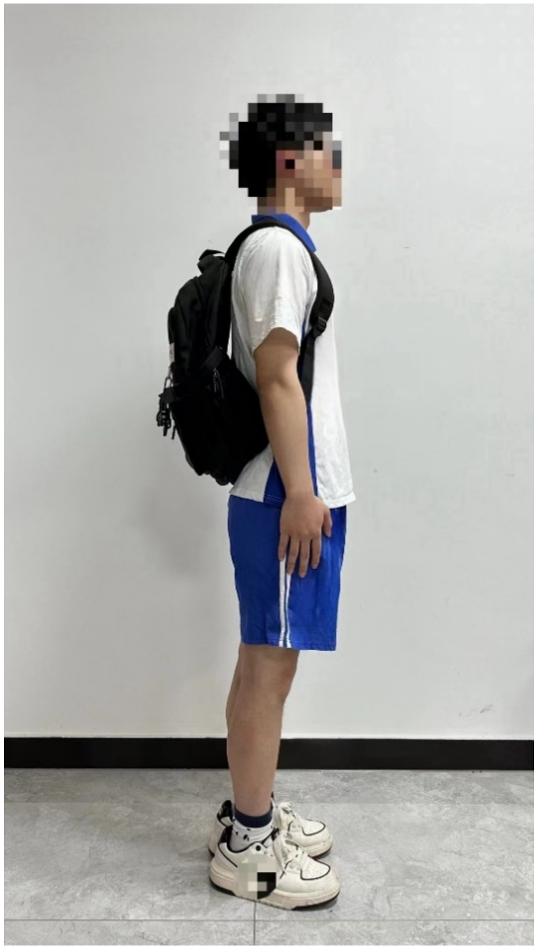

School uniform, female

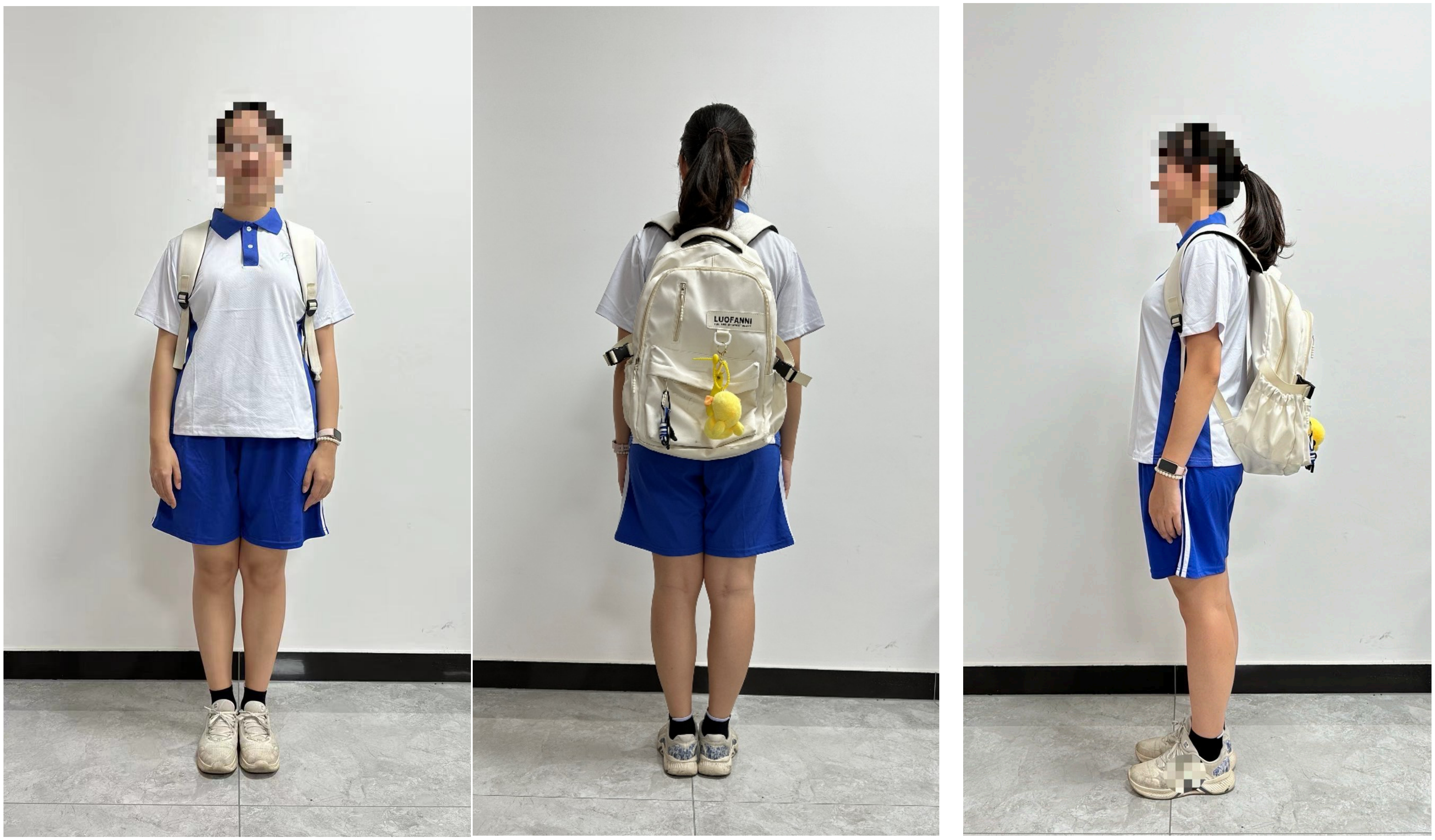

Casual attire, male

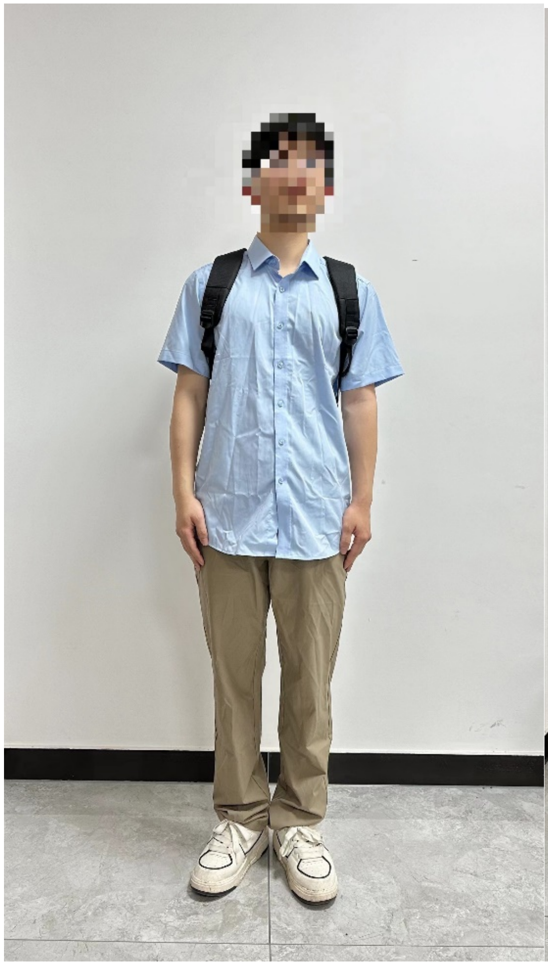

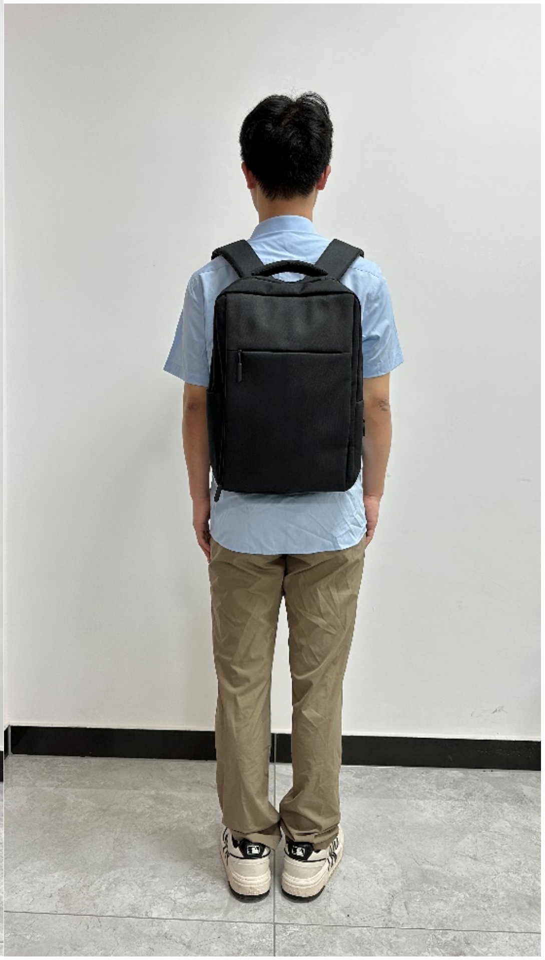

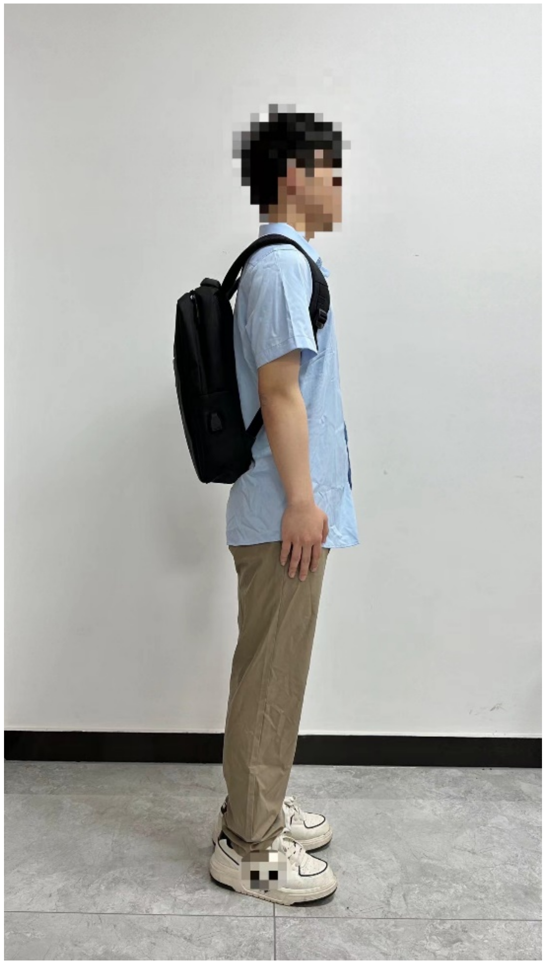

Casual attire, female

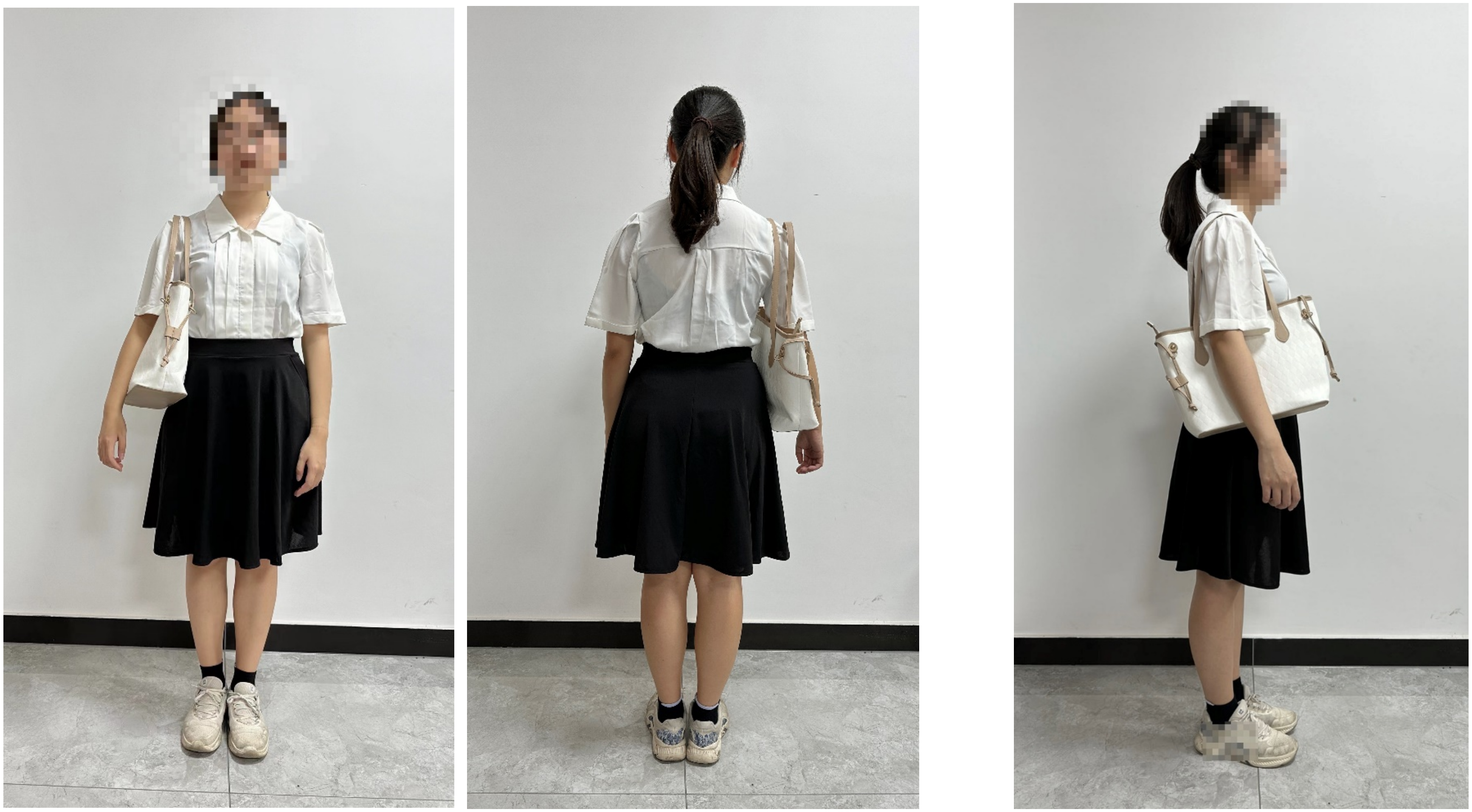

Appendix 4 Assessing tool

E-cigarette store survey questionnaire

Basic Information

1. Survey date [Fill in the blanks] *-------Click to confirm

____

2. Simulated buyer's name [Fill in the blank] *-------Fill in the name

____

3. City where you are located [Fill in the blanks] *------Please fill in "Beijing" in the format, do not fill in "Beijing City"

____

4. Store ID：___________________ [fill in the blank] *------- Each store corresponds to an ID. Note that there may be e-cigarette stores very close to each other in practice, and the store names might not be easily distinguishable. Please check carefully.

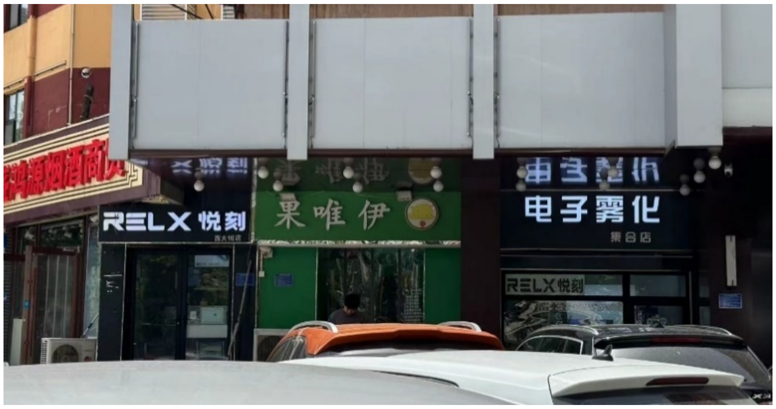

5. Location of store: (automatically fill the building location by the GIS function embedded in the Questionnaire Star) [Fill in the blank] *

____

6. The simulated buyer's attire today [single-choice question] *

○Student uniform

○Casual attire

Outside Store Observation

7. Is it possible to study this store? *[Single choice question]

○Yes;

○No, unable to locate the store based on the geographic information. You can follow the map navigation to the building and walk around the area to try to find it. If you still cannot find it, contact the study coordinator to add a new store;

○No, possible danger in surroundings was perceived, such as remote alleys with no pedestrians at all;

○No, store closed upon arrival, even though it was confirmed to be in business before. Note that some unopened stores can be observed, such as in pictures 1 and 2 below;

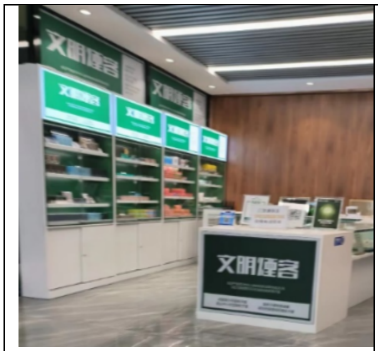

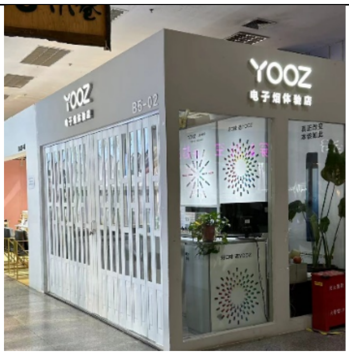

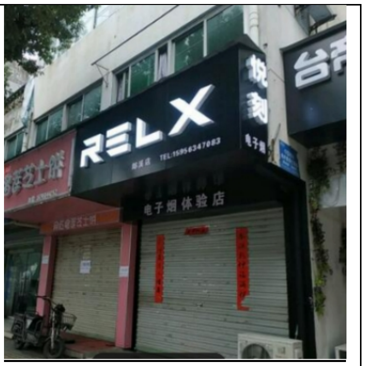

○No, other reasons ________________ * (Please skip to the end of the questionnaire and submit your answer sheet).

8. The type of stores is [single choice question] *

○Open stores in shopping malls ------based on whether the counter can be accessed from the pedestrian area;

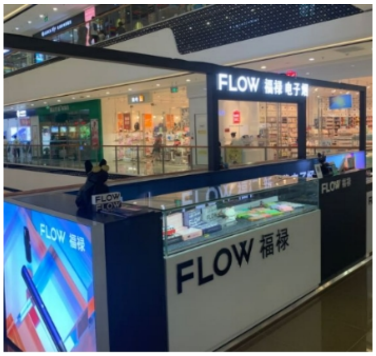

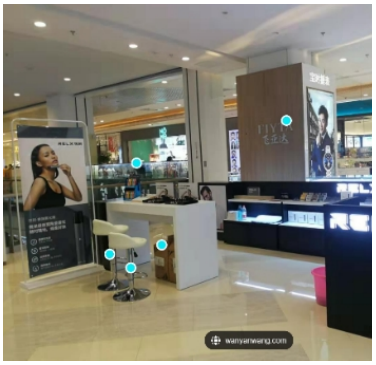

○Enclosed stores in shopping malls;

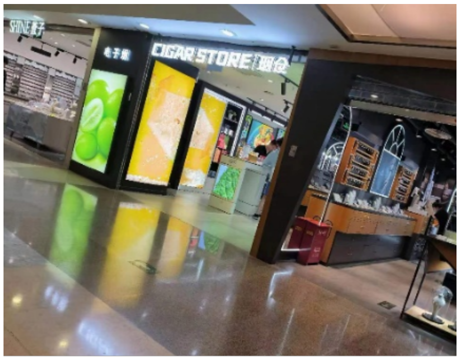

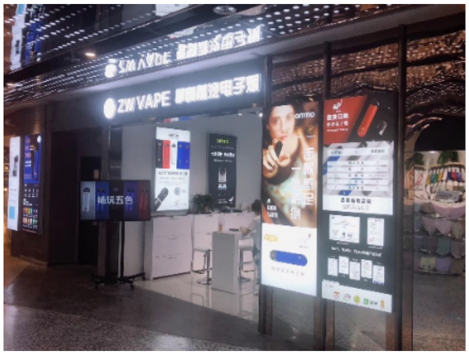

○Convenience store or small supermarket on the street — not primarily selling e-cigarettes;

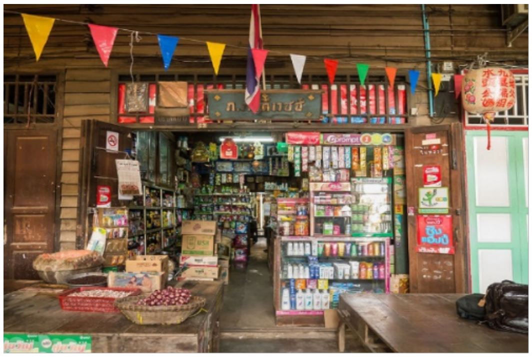
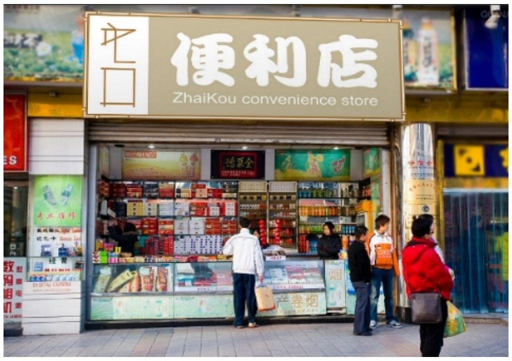

○E-cigarette store with independent stores on the street

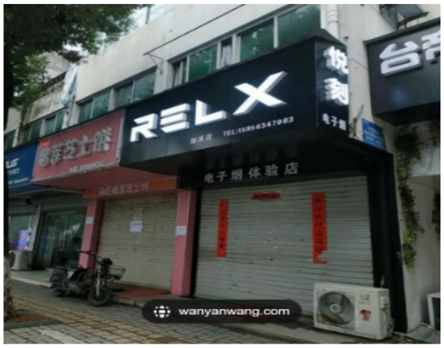

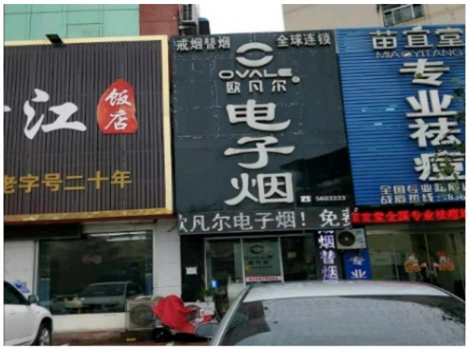

○E-cigarette store under an office building ------ [please note the distinction from an e-cigarette store that is an independent store on the street];

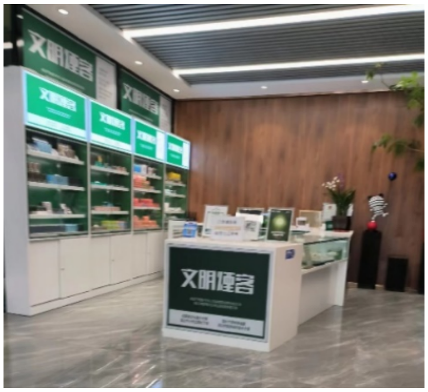

○Other type _________________ *

9. Are any of the following places near the e-cigarette store (look around and within sight)? [Multiple choice questions] *----- [Look around twice and mentally count 5 or more stores]

□School

□Shopping mall entrance, stairs-----This refers to the e-cigarette retail store in the mall

□Trendy toy store

□Beverage shop

□Female-only stores such as nail salons and cosmetics stores

□Internet cafe

□Bar

□Video arcade

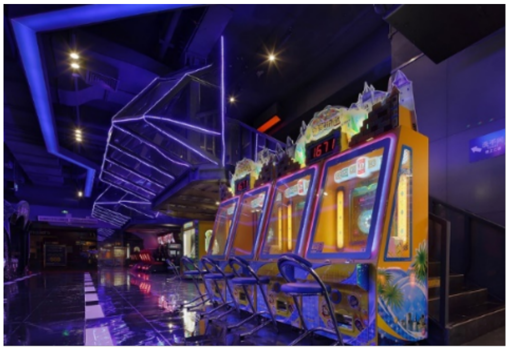

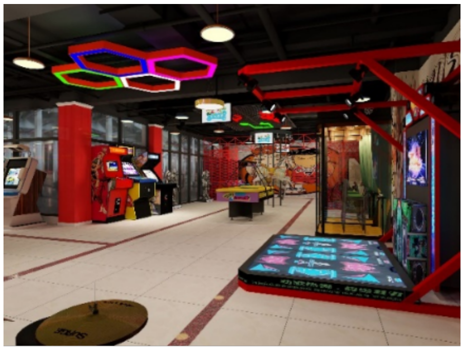

□Mobile phone store-----primarily sells mobile phone cases, data cables, and screen protectors.

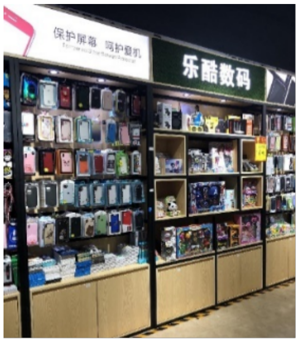

□Others (such as: large supermarkets, clothing stores) ___________----- （i.e. next to the entrance of large supermarkets）

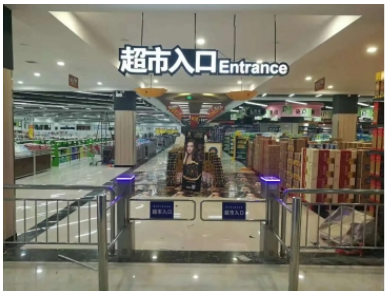

□None of the above

10. In which of the following ways does the exterior decoration of the store attract the attention of passers-by? [Multiple choice questions] *----Brand names or store names such as "RELX" do not count as advertising.

□General print advertising (posters, banners, flyers, etc.)

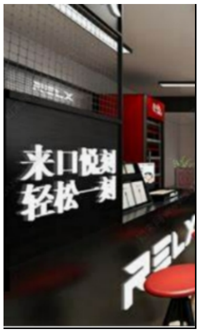

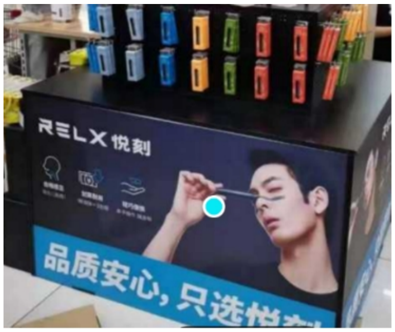

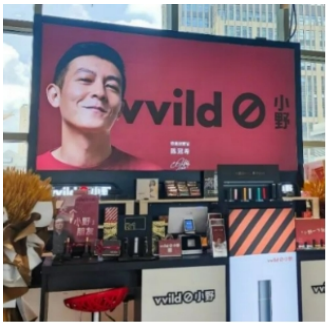

□Backlit or illuminated advertising-----refers to advertising that features LED lighting, video, and short clips accompanied by dynamic or static text display. [Note: When not illuminated, it is considered standard printed advertising.]

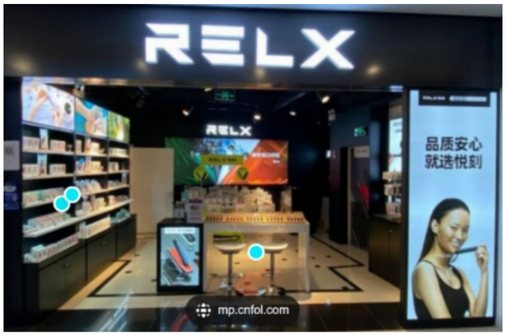

□Three-dimensional advertising, such as large fixtures — refers to large e-cigarette installations used in e-cigarette stores for product promotion or promotional activity displays, including large props, dolls, etc. (see the figure below).

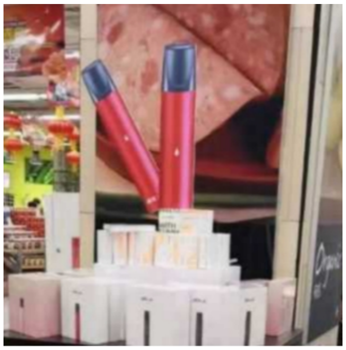

□Other_________________

□No advertising

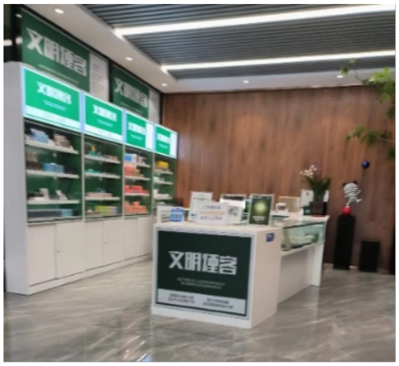

11. Area of large billboards: [Fill in the blanks__________] *---- Note: Take the prescribed poster as the first figure below. There may be more than just e-cigarettes in the advertisements in the second figure below. If there are too many advertisements in the third figure below, fill in 10. [For Question #10 above, if "Others" or "No advertising" is chosen, please fill in 0 for Question #11.]

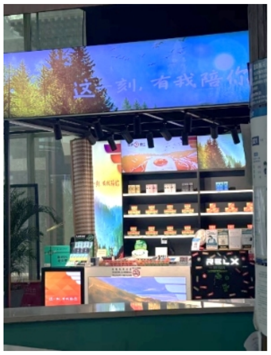

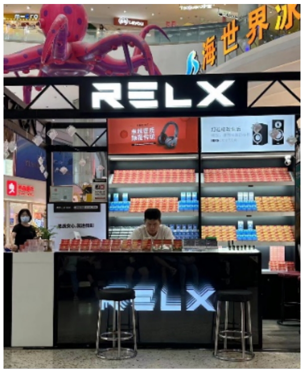

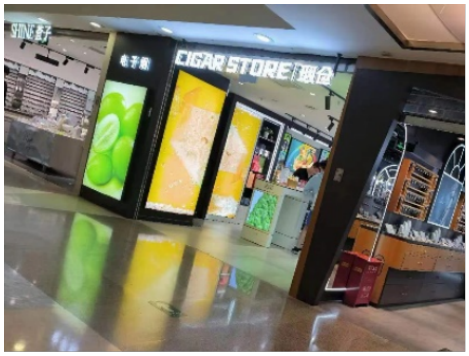

12. Are there any billboards with the following characteristics outside e-cigarette stores? [Multiple choice questions] *

□Use of celebrity endorsements

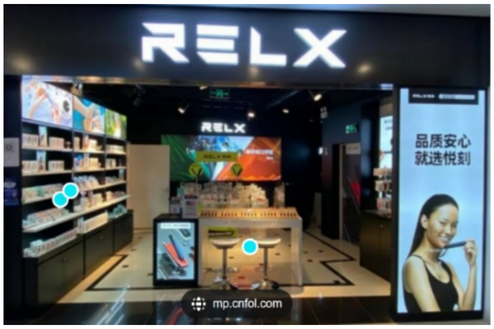

□Scenic photos, such as mountains, rivers, trees, and blue sky

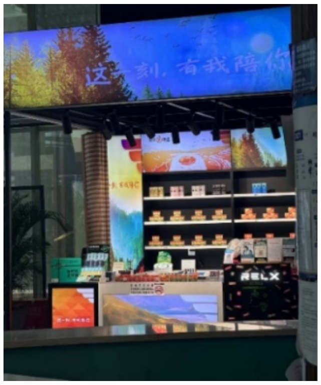

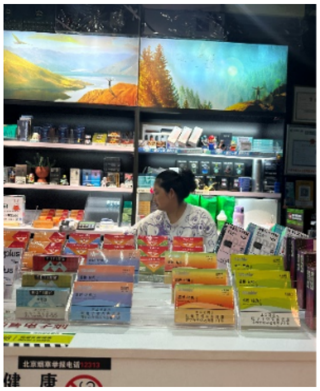

□Contains images of cartoon characters or animals ---- on billboards

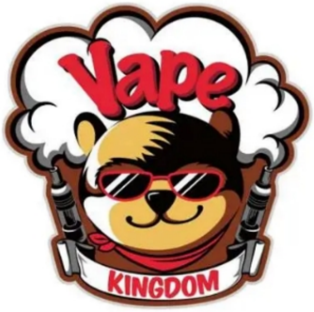

□Price discounts or promotional activities-----advertisements for price discounts or promotions often include phrases such as 'limited-time offer,' 'X% off,' 'price reduction,' 'buy and get xxx free,' 'grab a bargain,' or 'carnival season.' and so on.

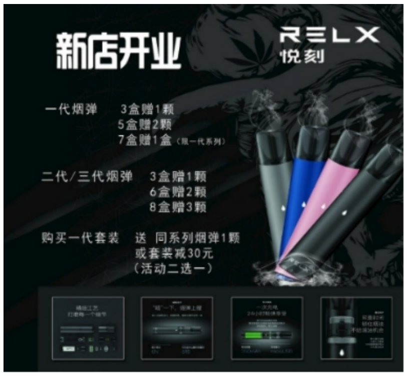

□Ads showing e-cigarette flavors, such as photos of fruit

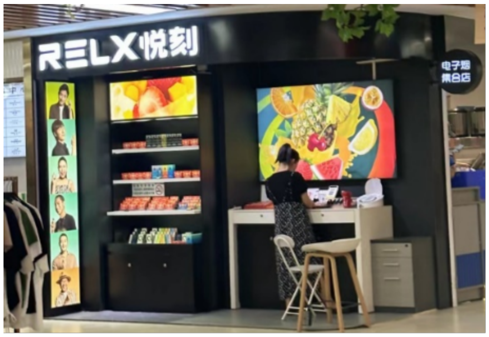

□Other__________________

Depends on options 1, 2, 3, or 4 of Question #10.

13. From the perspective of pedestrians, is there a recognizable "No Use by Minors" sign [Single Choice Question] *----The following large warning signs or clearly visible signs that can be seen from the outside

○Yes

○No

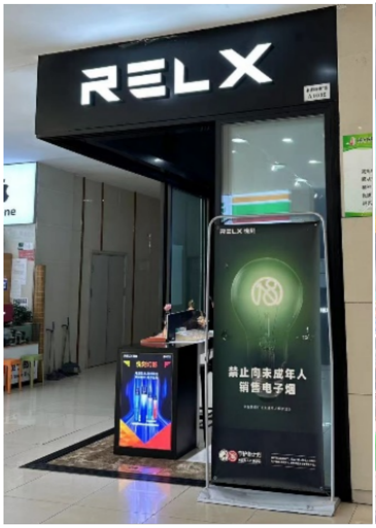

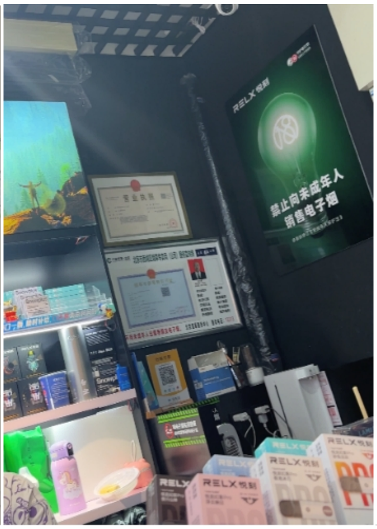

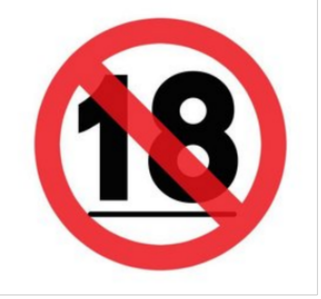

In-store communication and records 1

14. Can you enter the store to observe and record? Please select one of the following [Single Choice Question] *

○Yes

○No, the e-cigarette store is not open (please skip to the end of the questionnaire and submit your answer sheet)

○No, the seller is not in the store (please skip to the end of the questionnaire and submit your answer)

○No, other ________________ * (please skip to the end of the questionnaire and submit your answer sheet)

15. Is the tobacco retail license visible [Single choice question] *

○Yes

○No

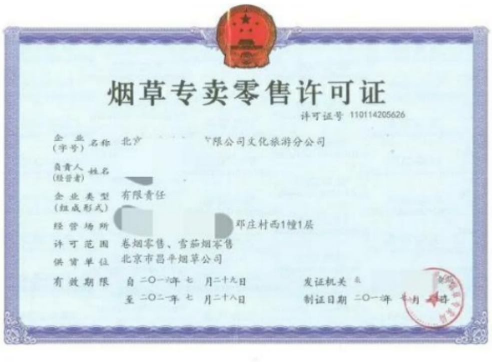
烟草专卖零售许可证
许可证号 110114205626
企业（字号）名称 北方[redacted]限公司文化旅游分公司
负责人（经营者）姓名 [redacted]
企业类型（组成形式） 有限责任
经营场所 [redacted]邓庄村西1幢1层
许可范围 卷烟零售、雪茄烟零售
供货单位 北京市昌平烟草公司
有效期限 自二〇一[illegible]年七月二十九日 至二〇二一年七月二十八日
发证机关 [redacted]
制证日期 二〇一[illegible]年[illegible]月[illegible]日

16. Is there a "No use by minors" slogan in the store (excluding slogans on product packaging) [Single-choice question] *

○Yes

○No

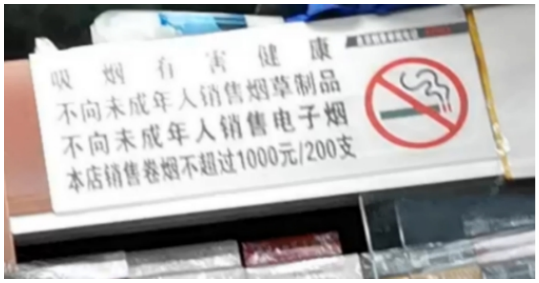

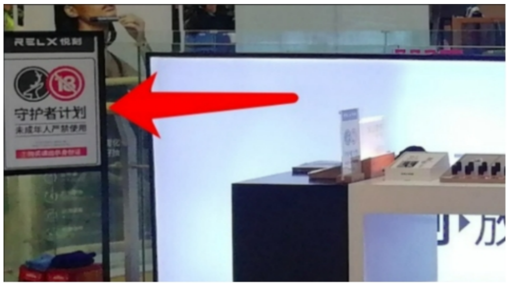

17. Are there any of the following health warning signs in the store (excluding slogans on product packaging) [Single choice question] *

○Yes, there is a "Smoking is harmful to health" sign

○Yes, there is a "E-cigarettes are harmful to health" sign

○Yes, both of the above exist

○No

18. Are there children's toys, car models, figurines, and/or other decorations placed on the shelves or counters? [Note that these decorations need to be targeted at teenagers.] [Single Choice Question] *

○Yes, specifically ________________ *

○No

19. Gender of the seller: (When there are multiple sellers, this question refers to the seller who communicates with the simulated buyer the most. The same applies to the questions below.)

○Male

○Female

20. Age of the seller (the simulated buyer needs to estimate) [Single-choice question] *

○18-24 years old

○25-29 years old

○30-39 years old

○>40 years old

21. Did the seller ask the simulated buyer if he or she was an adult? [Single Choice Question] * -----Questions such as "Are you an adult?" "Are you over 18?" "How old are you?" "Are you a student?" etc.

○Yes

○No

22. Did the seller ask to see the simulated buyer's ID? (If the seller requires you to fill in identity information on the system, please select Yes.) [Single Choice Question] *

○Yes

○No

23. Did the seller ask the simulated buyer to leave? (No sales communication) [Single Choice Question] * ----- If the seller does not check the age until the simulated buyer pays, the sales communication has been completed at this point. In this case, you should select "No" and continue to fill in the questionnaire.

○No

○Yes, the seller asked you (the simulated buyer) to leave without asking about their age. (Please skip to the end of the questionnaire and submit your answer.)

○Yes, the seller asked you (the simulated buyer) to leave after asking their age. (Please skip to the end of the questionnaire and submit your answer.)

○Yes, the seller asked you (the simulated buyer) to leave after knowing that he or she did not have an ID card. (Please skip to the end of the questionnaire and submit your answer.)

In-store communication and records 2

24. Does the e-cigarette store sell the following types of e-cigarettes and accessories [Multiple choice questions] **

□Disposable e-cigarettes ----- Disposable e-cigarettes refer to those e-cigarettes that cannot be disassembled, refilled, or changed by the user and are thrown away after use. They can also be rechargeable. For example, the seller might describe them to you as "rechargeable for 1,000 puffs."

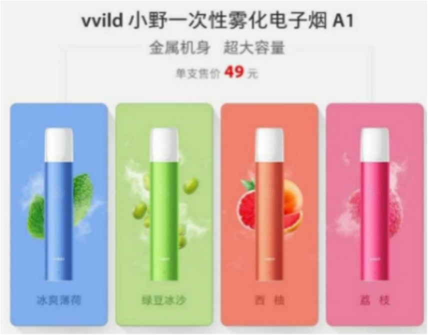

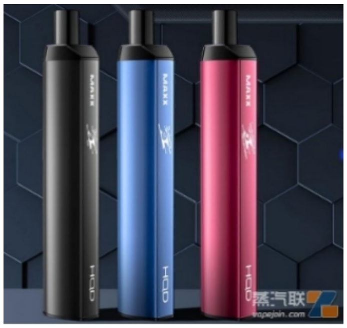

□Reloadable e-cigarettes and/or cartridges ----- Reloadable e-cigarettes usually consist of two parts: a cigarette rod and a cigarette cartridge. The cigarette rod can be recharged and reused, while the cigarette cartridge is thrown away after use.

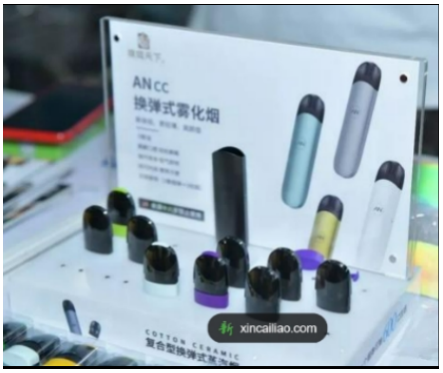

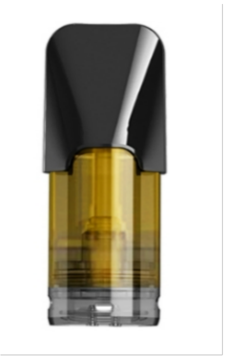
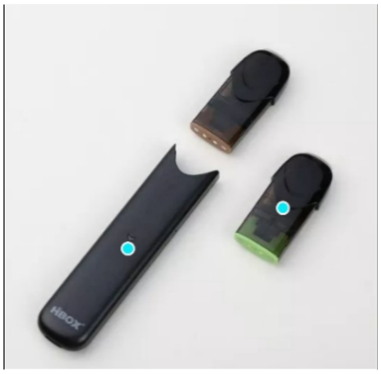

□Open e-cigarettes that the user refills with e-liquid himself or herself ----- The e-liquid tank of open e-cigarettes is open and refillable. It can be refilled 3-6 times for continued use. The atomizer has a long service life.

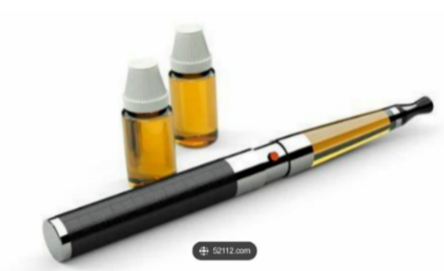
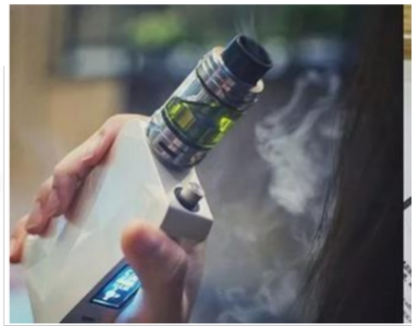

□Auxiliary accessories for e-cigarettes, such as protective cases, lanyards, etc.

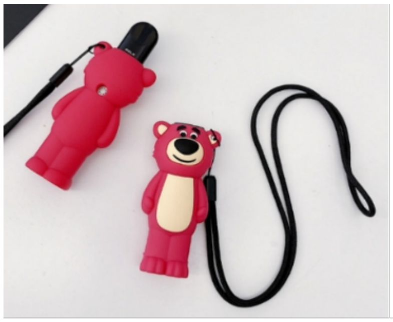
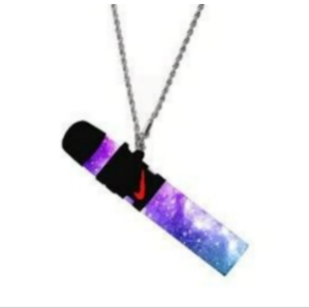
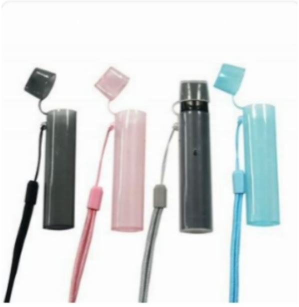

□Other, __________________

25. Other items sold in the e-cigarette stores: [Multiple choice questions] *

□No products other than e-cigarettes are sold

□Power bank rentals

□Addictive substances, such as tobacco, alcohol, and betel nut

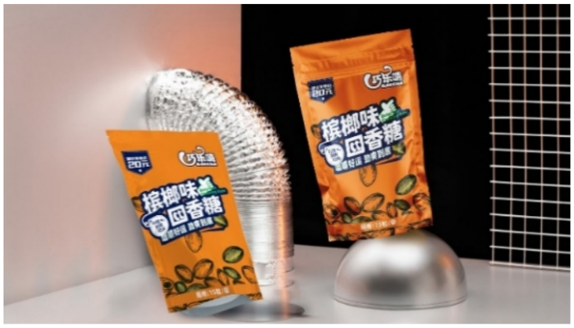

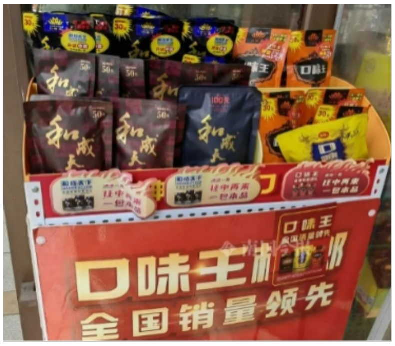

□Other ___________________

26. Did the seller proactively dissuade the simulated buyer from trying e-cigarettes? [Single Choice Question] * ----- The seller will tell you (the simulated buyer) that e-cigarettes are harmful, addictive, impossible to quit, don't try them, etc., in an attempt to discourage you from buying them.

○Yes

○No

27. The most popular flavor recommended to you (the simulated buyer) by the seller is [fill in the blank] * ----- Ask the seller, "With so many varieties, what are the differences?" and then, "Which one sells better?"

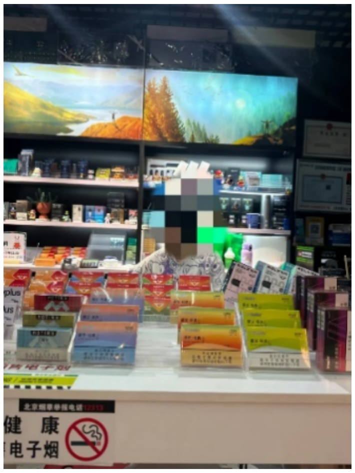

____

28. The second most popular flavor recommended to you (the simulated buyer) by the seller is [fill in the blank] * ----- If the seller only recommended one flavor in the previous question, you can fill in "none" for this question.

____

29. Is there a "Free Trial" sign or disposable filters in the store [Single-choice question] * -----Pay attention to whether there are disposable filters at the cashier]

○Yes

○No

30. Did the seller proactively mention the free trial? [Single-choice question] * ------The seller had a higher chance of letting you (the simulated buyer) try it, and might even assemble the e-cigarette and hand it over to you.

○Yes

○No

31. Are illegal flavored e-cigarettes sold? [Single Choice Question] * ----- Usually, illegal flavored e-cigarettes are not placed on display, lack obvious warning slogans on the packaging, and the product may be labeled in English.

○Yes

○No

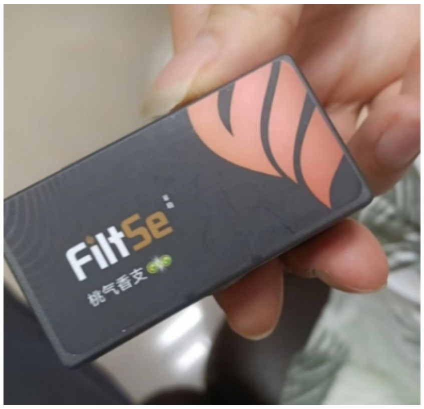

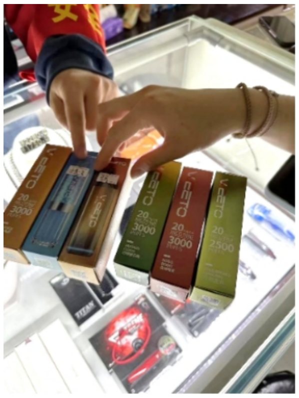

32. Does the e-cigarette store sell illegal e-cigarettes with the following flavors [Multiple choice questions] *

□Fruit flavor

□Candy flavor

□Food or drink flavors

□Mint flavor

□herbal vaporizer

□Other, ________________

□None of the above

Question #32 depends on option 1 of Question #31.

33. Did the seller proactively mention illegal e-cigarette products such as "milk tea cups" and "Coke cans" [Single-choice question] *

○Yes

○No

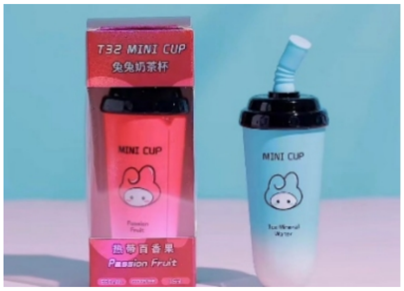

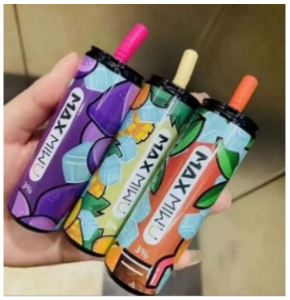

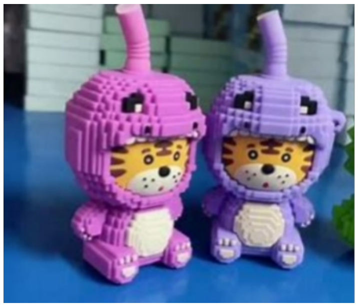

34. Does the information delivered to you (the simulated buyer) by the seller include the following? (Select in order) * ----- Use "E-cigarettes are very popular recently..." to potentially guide the seller to describe the reasons for the popularity of e-cigarettes.

[ ] None

[ ] E-cigarettes are less harmful

[ ] E-cigarettes can be used in more places

[ ] E-cigarettes are easily accepted by people around them

[ ] E-cigarettes have a variety of flavors

[ ] E-cigarettes do not produce unpleasant odors

[ ] Other, _________________

35. Are e-cigarettes promoted in the following ways? [Multiple choice questions] * ----- Ask "Are there any promotions?"

□ Price discounts (including coupons, limited-time activities, group purchase discounts, etc.)

□Free products

□Small gifts

□Membership activities, points rewards

□Others _______________

□None of the above

36. The lowest price of e-cigarette products is: [Fill in the blanks] * ----- If the cheapest one is a disposable e-cigarette, buy it and fill in the price. If there are only reloadable e-cigarettes in the store, you need to fill in the sum of the prices of the cartridges and the cigarette rods.

____

37. Did you (the simulated buyer) successfully buy e-cigarettes [single-choice question] *

○Yes

○No

38. Did the seller take the initiative to add your (the simulated buyer's) contact information?

[Single Choice Question] * ----- Pay attention to whether the seller took the initiative; you (the simulated buyer) should not take the initiative to ask. Please be sure not to agree to add WeChat.

○WeChat (a mobile instant messaging software)

○QQ mobile (a mobile instant messaging software)

○Phone number

○Other _________________

○No

39. Does the seller proactively provide online shopping services in an attempt to send e-cigarette products through social platforms? [Single Choice Question] * ----- If you were asked to add WeChat in the previous question, ask, "Why add WeChat?" If the seller says that he or she can send it to you online by adding WeChat, then select "Yes" for this question. Be sure to find a reason to refuse to add WeChat, such as replying, "No, I will buy it myself when I have time."

○Yes

○No

40. Is the contact information (including WeChat, phone number, etc.) publicly displayed in the store? [Single Choice Question] * ----- Usually, this is a small sign on the checkout counter, or it may be a brand WeChat group.

○Yes

○No

41. Do you (the simulated buyer) have any other notable observations that you would like to add? [Fill in the blanks] *

____

Appendix 5 Telephone protocol

For training the personnel making phone calls, you are attempting to gather information about e-cigarette stores over the phone. You can pretend to be a buyer looking to purchase e-cigarettes. Once you have collected all the information about the e-cigarette stores on the list, please make sure to keep all of it.

Soft Script: Speak cautiously, pretending that you are a buyer attempting to purchase e-cigarettes in the store. You would like to get more information about the e-cigarette stores, such as location, address, and opening hours. Do not ask questions proactively to avoid raising the seller's suspicions. Mention other issues as little as possible to minimize mistakes.

Once the call connects, ask: "**Hello, is your shop still open? I want to buy some e-cigarettes.**" If they answer it's closed, lament: "**Ah, why is it closed... where can I buy some now...**" Pay attention to whether they voluntarily mention:

- Having changed their type of business;
- Providing other contact methods (adding their WeChat, a mobile instant messaging software, or recommending another merchant's phone number);
- Offering online sales (rapid delivery, courier);
- Mentioning illegal flavored e-cigarettes (milk tea cup, bear cup, planet cup, etc.).

If the seller answers the store is closed, you can respond with, "Never mind, I'll look elsewhere."

—> Hang up the call to end it.

Answer the call	Under normal business	Change business	Provide additional contact information	Selling online	Selling illegal flavors	Key information records
1	0	1	1	1	1	"Branded products are not selling well, but

						milk tea cups have many flavors." "Add me on WeChat and I will send them to you by local courier."
1	0	0	0	1	0	"The store is closed, but if you need it, add me on WeChat and I will contact my friends to sell it to you."

(1) For the e-cigarette stores that are open —>

You said: "Okay, then I'll come over and see if I have time. Are you at [xxxxx] (check address)?"

—> Hang up to end the call.

(2) Record if no one answers your call:

After dialing, wait until the voice prompt says "Sorry, the number you dialed is temporarily unavailable." This is considered unavailable, and you should mark 0 in Excel.

—> Try to call again after 15 minutes.

—> If the call is connected, follow the soft script above for communication. If still not connected, hang up to end the call.

(3) Record shutdown:

—> If the call is connected, follow the soft script above for communication. If the phone is turned off or disconnected, hang up to end the call.

Note：

Stop calling once you have a list of 40 e-cigarette stores open in each metropolitan area. Submit the list of 40 e-cigarette stores to your supervisors.

For various problems and situations encountered, if the issue is special or difficult to resolve, you can contact your supervisors at any time. Do not force the task to be completed.

Appendix 6 Recruitment poster for simulated buyers

The China Center for Health Development Studies at Peking University will conduct a survey on the behaviors and marketing strategies of e-cigarette retail stores in several major metropolitan areas in China. The survey is expected to begin from July 22-25 and last for about three weeks. Offline training will be provided before the official start date. Scan the QR code to sign up, and you will gain valuable research experience and generous compensation. This is also a great opportunity to visit various places in China and explore local delicacies.

Recruitment Criteria:

University students aged 18 or 19 currently studying in Beijing.

Have never used cigarettes or e-cigarettes.

Possess good communication skills and a sense of responsibility, and can commit to the training and the entire survey period.

Benefits:

Accommodation and transportation fees covered, with a daily compensation of CNY 350.

Outstanding researchers have a chance to receive a surprise gift pack!

After the survey, the research team will verify the quality of the questionnaires and issue a certificate of practice.

QR Code for Registration:

We sincerely look forward to your participation! If you have any questions, feel free to contact us via phone or WeChat: 15010987573.

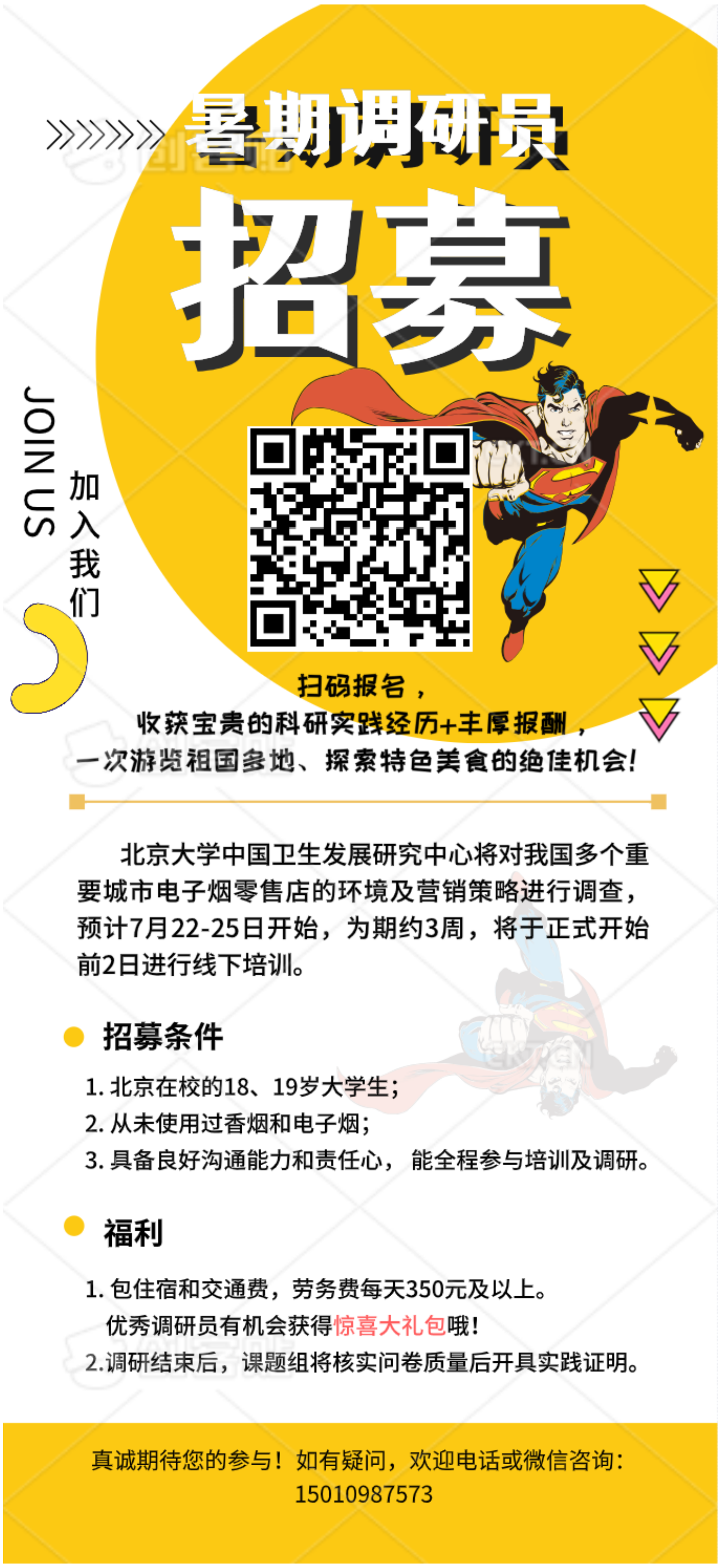
暑期调研员
招募
JOIN US
加入我们
扫码报名，
收获宝贵的科研实践经历+丰厚报酬，
一次游览祖国多地、探索特色美食的绝佳机会！
北京大学中国卫生发展研究中心将对我国多个重要城市电子烟零售店的环境及营销策略进行调查，预计7月22-25日开始，为期约3周，将于正式开始前2日进行线下培训。
招募条件
1. 北京在校的18、19岁大学生；
2. 从未使用过香烟和电子烟；
3. 具备良好沟通能力和责任心， 能全程参与培训及调研。
福利
1. 包住宿和交通费，劳务费每天350元及以上。
优秀调研员有机会获得惊喜大礼包哦！
2.调研结束后，课题组将核实问卷质量后开具实践证明。
真诚期待您的参与！如有疑问，欢迎电话或微信咨询：
15010987573

Appendix 7 Interview process for simulated buyers

The interview will be conducted online via Tencent Meeting. The interview time for each student (the simulated buyer) is about 10 minutes. The process is as follows:

1. Self-Introduction: Please give a brief self-introduction.
2. Recruitment Inquiry: How did you hear about our recruitment? Why do you want to participate in our research activities, and what do you hope to gain?
3. Research Overview: Introduce the key content of this research. Do you have any concerns or difficulties about participating fully? If there are difficulties that cannot be overcome, we suggest not participating.
4. Dress Code and Confidentiality: Additional information on dress code and confidentiality agreement (scientific rigor, data authenticity, innovation in research projects).
5. Safety Emphasis: Finally, we emphasize safety issues. We hope everyone will cooperate with us, return on time in the evening, and communicate with us if you have any arrangements or places you need to go.

Appendix 8 Survey sequence of the metropolitan areas for three teams

Team	Survey sequence
**Team A**	Harbin - Changchun - Shenyang - Dalian - Qingdao - Jinan - Shijiazhuang - Taiyuan - Beijing - Tianjin - Hohhot - Lhasa
**Team B**	Nanjing - Shanghai - Hangzhou - Ningbo - Fuzhou - Xiamen - Shenzhen - Guangzhou - Nanchang - Hefei - Urumqi - Xining - Yinchuan
**Team C**	Zhengzhou - Wuhan - Changsha - Nanning - Haikou - Guiyang - Kunming - Chongqing - Chengdu - Xi'an - Lanzhou

Appendix 9 Code for web crawling

```
#Rversion 4.04
setwd("E:/R")
library(xml2)
library(rvest)
library(dplyr)
library(stringr)
library(rjson)
library(jsonlite)
library(scales)
library(ResourceSelection)
library(openxlsx)#xlsx
library(sampling)
rm(list = ls())

# collect address and phone number using AutoNavi ----------------------------------------------------
key <- c("AutoNavi Key (got from AutoNavi API)")
storedf <- as.data.frame(matrix(nrow = 0,ncol = 8))
surveywords <- c("电子烟店","电子烟零售店","电子烟体验店","电子烟集合店","电子烟专卖店","电子烟直营店", "雾化","蒸汽烟","vape","电子烟旗舰店",  "悦刻","魔笛","雪加","小野","铂德","柚子电子烟","徕米","MR迷睿","VTV","福禄FLOW", "RELX","MOTI","YOOZ","snowplus","vvild","Boulder","LAMI")

gGetstore <- function(keywords,city,page){
  url <-  paste0("https://restapi.amap.com/v3/place/text?keywords=",keywords,"&city=",
                 city,"&citylimit=true&offset=20&page=",page,"&key=",
                 key,"&extensions=all")
  data <-  read_html(url, encoding='utf-8') %>% html_text()
  datalist<- fromJSON(data)
  address_islist <- 0 #check
  tel_more <- 0
  if (datalist$status=="1") {
    if (datalist$count!="0") {
      if(is.list(datalist$pois$address)){
        datalist$pois$address <- sapply(datalist$pois$address,"[",1)
        address_islist <- 1
      }
      df <- cbind(datalist$pois$id, datalist$pois$name, datalist$pois$address,
                  datalist$pois$location,datalist$pois$cityname,datalist$pois$adname,
                  datalist$pois$typecode)
      if(all(lengths(datalist$pois$tel)<2)){
        tel <- sapply(datalist$pois$tel,"[",1)
```

```
        }else{tel_more <- 1}

        df <- cbind(df, tel)
      }else{df <- 0}
    }else{df <- 1}
    storeout    <- list(df=df, two=address_islist, three=tel_more)
    return(storeout)
}

#run
cityname <- c("北京")    #try each
store_city <- data.frame()
address_list <- rep(0,length(surveywords))
searchnm <- rep(0,length(surveywords))
storedf_class <- rep(0,length(surveywords))
tel_list    <- rep(0,length(surveywords))
for (j in 1:length(surveywords)) {
    storedf <- data.frame()
    for (i in 1:100) {
      storelocation <- gGetstore(surveywords[j],cityname,i)
      storedf2 <- storelocation$df
      if(storelocation$two==1){address_list[j]    <- 1}
      if(storelocation$three==1){tel_list[j] <- 1}
      if (is.numeric(storedf2)){
        searchnm[j]    <- i
        break
      }else{
        storedf <- rbind(storedf,storedf2)
      }
    }
    storedf_class[j] <- class(storedf)
    store_city <- rbind(store_city,storedf)
    if(dim(storedf)[1] !=0){
      colnames(storedf) <- c("id","name","address","location","city","adname","typecode","tel")
write.xlsx(storedf,paste0("E:/e_cig/summer_survey/retailer_list/list_",surveywords[j],"_",cityname,".xl
sx"),            append=T,    overwrite=T)
    }
}
write.xlsx(store_city,paste0("E:/e_cig/summer_survey/retailer_list/city2/list_",cityname,".xlsx"),overwr
ite = T)
store_city <- unique(store_city)
write.xlsx(store_city,paste0("E:/e_cig/summer_survey/retailer_list/city/list_",cityname,".xlsx"),overwri
te = T)
```

Statistical Analysis Plan

A Large-Scale Randomized Control Trial to Determine the Effects of Dress by Adolescents on Electronic Cigarette Retailer Behaviors in Regulation Compliance in China

Registered ID: NCT05962411

Version Date: September 3rd, 2023

Statistician: Yang Wang, Hai Fang

APPROVAL

**Authors**	**Signature**	**Date**
Prof Yang Wang<br><br>Peking University	汪洋	Sept. 03. 2023
Prof Hai Fang<br><br>Peking University	方海	Sept. 03, 2023

ABBREVIATION

Abbreviation or Term	Definition/Explanation
E-cigarette	Electronic cigarette
RCT	Randomized controlled trial
ID	Identification card

Table of Contents

APPROVAL **2**

ABBREVIATION **3**

1. INTRODUCTION **5**

1.1. OVERVIEW 5
1.1.1. Study Design *5*
1.1.2. Randomization *6*
1.1.3. Blinding *7*
1.2. STUDY OBJECTIVES 7
1.2.1. Primary Objective *7*
1.2.2. Secondary Objectives *7*
1.2.3. Exploratory Objectives *8*
1.3. STUDY HYPOTHESES 8
1.4. SAMPLE SIZE 8

2. STATISTICAL AND ANALYTICAL PROCEDURE **10**

2.1. STUDY SUBJECTS 10
2.2. STUDY VARIABLES 10
2.2.1. Outcomes *10*
2.2.2. Covariates *11*
2.3. STATISTICAL METHODS 12
2.3.1. General Considerations *12*
2.3.2. Analysis of Primary Outcome *12*
2.3.3. Analysis of Secondary Outcome *12*
2.3.4. Analysis of Other Outcomes *13*
2.3.5. Subgroup Analysis *13*

3. SOFTWARE DOCUMENTATION **14**

4. REFERENCE **14**

1. Introduction

The Statistical Analysis Plan (SAP) aims to provide a comprehensive and detailed description of the methods and strategies to be adopted for analyzing data collected from e-cigarette stores in China. The SAP is finalized prior to database lock to ensure the credibility of the study's findings.

1.1. Overview

1.1.1. Study Design

This study is a two-armed, national-level, open-label cluster RCT conducted among e-cigarette stores located in 36 metropolitan areas across mainland China (including 4 municipalities directly under the Central Government, 27 provincial capitals, and 5 cities specially designated in the national plan). This RCT will adopt the mystery shopping approach to measure the effect of adolescent buyers' type of attire (school uniforms vs. casual attire) on the likelihood of sellers' compliance with the minimum age restriction for e-cigarette sales, defined by both age verification and sales. The e-cigarette stores will be randomly assigned to the intervention group or control group at a ratio of 1:1. Each store in the intervention group will receive a simulated buyer dressed in a school uniform, while each store in the control group will receive a simulated buyer dressed in casual attire. The simulated buyers in this study are adolescents aged 18-19 years old. The simulated buyers will attempt to purchase e-cigarette products and record the behaviors and marketing claims of the sellers, as well as store internal and external characteristics.

Eligible stores as study subjects will be identified from the AutoNavi Map through keyword searches. Stores are eligible for enrollment if they have reachable contact information and claim to be open for business on a regular basis when contacted. The expected number of e-cigarette stores sampled in each metropolitan area is approximately 32, with an additional 8 e-cigarette stores serving as backups, if the

number allows, for a minimum expected total enrollment of approximately 1,080 retailers. In addition, we plan to conduct the same trial for cigarette stores and perform a 1:1 match of e-cigarette stores and cigarette stores, allowing us to compare the business practices of e-cigarette stores and cigarette stores between the two attire groups.

1.1.2. Randomization

Generating clusters to which the stores belong: Considering traveling time and the efficiency of data collection, this trial will use a cluster randomization approach rather than simple randomization. Investigators will first map all the 40 selected stores within each metropolitan area and then divide them into clusters containing the same number of e-cigarette stores (approximately 5 stores per cluster), taking into account their locations and public transportation. Each cluster will then be digitally labeled, from area 1 to area 8. The labeling of the clusters will follow the principle of from top to bottom, and from left to right on the map.

Assigning clusters to treatment and control groups: Each cluster will be randomly assigned to one simulated buyer, and the type of attire (school uniforms or casual attire) they wear on a specific day will also be randomized. In this way, the e-cigarette stores will ultimately be allocated at a 1:1 ratio to either the intervention or control group.

- Specifically, the simulated buyers (data collectors) to be recruited are 18 to 19 years old, with a 1:1 ratio of males to females. To reduce bias associated with the personal characteristics of the simulated buyers, they will be randomly assigned a new digital number for each metropolitan area, which will be used for matching the clusters.

- The type of attire worn by each adolescent on a specific day will be pre-determined using Excel's random number generator within each sex, ensuring that each

adolescent has an equal probability of wearing school uniforms and casual attire during the entire study.

1.1.3. Blinding

It will be impossible to blind study subjects (e-cigarette stores) regarding the assignment to the intervention or control group, as letting sellers in the e-cigarette stores see the buyers' attire is part of the treatment. However, sellers will not be aware of their participation in this trial due to the mystery shopping approach adopted.

The trial team, consisting of both simulated buyers and coordinators, will also not be blinded to the e-cigarette stores in the intervention and control groups, as there is no way to prevent them from knowing which e-cigarette stores have simulated buyers wearing school uniforms or casual attire on each day.

The statistician conducting the analyses will have no direct contact with the e-cigarette stores but will not be blinded to the treatment allocation.

1.2. Study Objectives

1.2.1. Primary Objective

To measure the effect of dressing in school uniforms (compared to casual attire) by adolescents on regulation compliance-related behaviors of e-cigarette sellers defined by both age verification and underage-cigarette sales.

1.2.2. Secondary Objectives

- To determine the impact of type of attire by adolescents on in-store communication between sellers and customers, specifically dissuading use.
- To discern the differences in regulation compliance-related behaviors between e-cigarette and cigarette retailers.

1.2.3. Exploratory Objectives

- To describe the regulatory compliance of e-cigarette and cigarette retailers across mainland China.
- To document the marketing and communication strategies adopted by e-cigarette and cigarette retailers, e.g. store location, advertisements, decorations and online delivery service, across mainland China.

1.3. Study Hypotheses

We hypothesize that dressing in school uniforms by adolescents will significantly increase the likelihood of e-cigarette sellers verifying the age of buyers in stores and reduce the probability of their illegal sales to minors.

The corresponding null hypothesis that will be tested is that dressing by school uniforms by adolescents will have no effect upon e-cigarette selling behaviors in regulation compliance.

1.4. Sample Size

Successful purchase rates of e-cigarette products for the school uniform group and casual attire group will be derived from the pilot study. Other parameters required for cluster-RCT sample size calculation are as follows:

- Randomization in a 1:1 ratio for the school uniform (intervention) group and casual attire (control) group
- $\alpha = 0.05$ (two-sided)
- power = 0.85
- cluster size = 4 (both for intervention group and control group)

- inter-cluster correlation coefficient = 0.5 (default)

However, considering the primary goal of this study is to characterize e-cigarette seller behaviors at a national level, we decide to set the final sample size to be approximately 1,080. This may be far more than the minimum sample required (about three times) for testing the effect of buyer attire on e-cigarette sales to adolescents in China.

2. Statistical and Analytical Procedure

2.1. Study Subjects

This study will be conducted across 36 major metropolitan areas in mainland China (including 4 municipalities directly under the Central Government, 27 provincial capitals, and 5 cities specially designated in the national plan). The expected number of study subjects sampled from each metropolitan area is approximately 32 e-cigarette stores, if the number allows, for a minimum expected total enrollment of approximately 1,080. This study will include cigarette stores for comparisons with e-cigarette stores. For each e-cigarette store, one nearby cigarette shop will be conveniently selected for matching, as cigarettes are widely available in convenience stores, supermarkets, and tobacco and liquor shops, making it impossible to systematically pre-identify these stores.

2.2. Study Variables

2.2.1. Outcomes

Primary Outcome Measure

The primary outcome is the sales of e-cigarettes to adolescent buyers, measured by the successful purchase of e-cigarette products from the stores without showing any age proof.

Secondary Outcome Measures

- The seller verbally inquiring about the simulated buyer's age
- The seller requesting ID cards from the simulated buyer for age verification
- The seller dissuading the simulated buyers from using e-cigarettes

Other Outcome Measures

- Illegal flavored products being sold: The simulated buyer will report if the seller mentions that flavored (non-national-standard) e-cigarette products are available in the stores.
- Online contact: The simulated buyer will report if WeChat account information is publicly displayed for adding contacts.
- Delivery service offered: The simulated buyer will report if the seller mentions that products ordered online can be delivered.
- Marketing claims: The simulated buyer will record the marketing claims made by the seller to promote the products

2.2.2. Covariates

The assessment tool should be designed primarily based on the Vape Shops Standardized Tobacco Assessment for Retail Setting (V-STARS)[1] and the Tobacco Advertising, Promotion and Sponsorship (TAPS) bans compliance guide[2], as well as published articles[3]. It will be further adapted by investigators who are familiar with Chinese e-cigarette retailers and laws and have observed store characteristics, to the Chinese context to collect information mainly on store internal/external environmental features (e.g., location, advertising, warning signs, and licensing), product availability, and seller behaviors. Detailed information on covariates is listed as below:

- Buyer characteristics: Sex, Age
- Seller characteristics: Sex, Age perceived by the simulated buyers (18-29, 30-40, >40 years old)
- Store characteristics: Store location (In mall, on street), region, age-of-sale warning sign presented, license to sell e-cigarettes presented, health warning presented, and advertising.

2.3. Statistical Methods

2.3.1. General Considerations

Summary statistics will be presented for both the treatment and control groups. Continuous variables will be summarized by the number of observations, mean, standard deviation, median, minimum, and maximum. Categorical variables will be summarized using frequency counts and percentages. Descriptive analyses of the distributions of the e-cigarette seller behavior outcomes between two types of attire (treatment and control groups), including successful purchases, inquiring about buyer age, requesting ID cards for age verification, and dissuading buyers from use, as well as store environment features, and sellers' demographic characteristics, will be conducted with t-tests and chi-square tests detecting statistically significant differences.

The significance level for comparisons between the two groups is set at α=0.05 (two-sided). Differences between the study groups will be considered statistically significant if the p-value is less than 0.05. All analyses will be performed using Stata MP 17.

2.3.2. Analysis of Primary Outcome

For the primary outcome, successful purchase rates will be analyzed using Chi-square tests to determine if the differences between the treatment group and the control group are statistically significant. Multivariate logistic regression models will be further employed to analyze the treatment effect, adjusting for covariates.

A national map depicting the geographic variations in the outcomes of successful purchase rates will be created using the R package DrawChinaMap.

2.3.3. Analysis of Secondary Outcome

Secondary outcomes, including inquiring about the buyer's age, requesting ID

cards for age verification, and dissuading buyers from use, will be analyzed in a similar manner to the primary outcome. Chi-square tests will be used to detect the statistical significance of differences between the two study groups, and logistic regression models, adjusted for covariates and appropriate for the distribution of the outcomes, will be employed. Additionally, similar national maps describing geographic variations will be generated.

2.3.4. Analysis of Other Outcomes

Other outcomes, including illegal flavored products, online contact, and delivery service offered, will be analyzed in a similar way as the primary outcome. Chi-square tests will be used to detect statistical significance in differences between the two groups, and logistic regression models appropriate for the distribution of the binary outcomes with covariate adjustments will be employed. Similar national maps describing geographic variations will be generated as well. For marketing claims and strategies, we will further employ thematic framework analysis to qualitatively analyze and describe the marketing methods and tactics of e-cigarette stores.

2.3.5. Subgroup Analysis

For matched e-cigarette and cigarette stores, we will employ McNemar’s tests to assess the distinctions in the outcomes between e-cigarette stores and cigarette stores. Rates of successful e-cigarette purchases stratified by age checking behaviors and buyer attire will also be reported. Planned subgroup analyses are intended to explore potential effect modifications of gender, age, metropolitan area and store types.

3. Software Documentation

All the statistical analyses will be conducted using Stata MP 17 (StataCorp, College Station, TX, US). The maps depicting geographic variations will be generated using DrawChinaMap (https://rdrr.io/github/psychbruce/drawMap/src/R/drawChinaMap.R).

4. Reference

1. Kong AY, Eaddy JL, Morrison SL, Asbury D, Lindell KM, Ribisl KM. Using the Vape Shop Standardized Tobacco Assessment for Retail Settings (V-STARS) to Assess Product Availability, Price Promotions, and Messaging in New Hampshire Vape Shop Retailers. Tob Regul Sci 2017;3(2):174-182. (In eng)
2. Polanska K, Kaleta D. Tobacco and E-Cigarettes Point of Sale Advertising-Assessing Compliance with Tobacco Advertising, Promotion and Sponsorship Bans in Poland. Int J Environ Res Public Health 2021;18(4) (In eng)
3. Nian Q, Cohen JE, Cui Y, Zhang S. Tobacco retailers around schools in 10 cities across China. Tob Control 2022 (In eng)
