# Supplementary material for: E-Cigarette Sales to School-Uniformed Adolescents in China: A Randomized Clinical Trial
Source: JAMA Netw Open. 2025 Oct 10;8(10):e2535623. doi: 10.1001/jamanetworkopen.2025.35623 (PMC12514632; doi:10.1001/jamanetworkopen.2025.35623)
Supplement: Supplement 2. — eTable 1. Buyer, Store, and Seller Characteristics by Types of Attire for Cigarette Store Visits, China eTable 2. Multivariate Logistic Regressions for Successful Purchases, Inquiring About Age, Requesting ID Card, and Dissuading Use, Cigarette Store Visits, China eTable 3. Parallel Mediation Analysis for Requesting ID Card and Inquiring About Age eTable 4. Subgroup Analyses of Successful Purchases, Inquiring About Age, Requesting ID Card, and Dissuading Use Across Sex, Age, and Region eFigure 1. A Sampling Distribution Map of e-Cigarette Stores eFigure 2. Male and Female Models in School Uniforms and Casual Attire eFigure 3. Differences in Successful e-Cigarette Purchases, Stratified by Types of Attire and Age-Verification Behaviors eFigure 4. Differences in Cigarette Sales to Adolescents by Types of Attire, China eFigure 5. Maps of Likelihood of Successful Purchases, Inquiring About Age, Requesting ID Card, and Dissuading Use in e-Cigarette Stores, China [file jamanetwopen-e2535623-s002.pdf]

Supplementary Online Content

Wang Y, Lv X, Laestadius LI, et al. e-Cigarette sales to school-uniformed adolescents in China: a randomized clinical trial. *JAMA Netw Open*. 2025;8(10):e2535623. doi:10.1001/jamanetworkopen.2025.35623

eTable 1. Buyer, Store, and Seller Characteristics by Types of Attire for Cigarette Store Visits, China
eTable 2. Multivariate Logistic Regressions for Successful Purchases, Inquiring About Age, Requesting ID Card, and Dissuading Use, Cigarette Store Visits, China
eTable 3. Parallel Mediation Analysis for Requesting ID Card and Inquiring About Age
eTable 4. Subgroup Analyses of Successful Purchases, Inquiring About Age, Requesting ID Card, and Dissuading Use Across Sex, Age, and Region
eFigure 1. A Sampling Distribution Map of e-Cigarette Stores
eFigure 2. Male and Female Models in School Uniforms and Casual Attire
eFigure 3. Differences in Successful e-Cigarette Purchases, Stratified by Types of Attire and Age-Verification Behaviors
eFigure 4. Differences in Cigarette Sales to Adolescents by Types of Attire, China
eFigure 5. Maps of Likelihood of Successful Purchases, Inquiring About Age, Requesting ID Card, and Dissuading Use in e-Cigarette Stores, China

This supplementary material has been provided by the authors to give readers additional information about their work.

© 2025 Wang Y et al. *JAMA Network Open*.

eTable 1: Buyer, Store, and Seller Characteristics by Types of Attire for Cigarette Store Visits, China

Characteristics	Overall (n=1059) Percent (95%CI)	School uniforms (n=526) Percent (95%CI)	Casual attire (n=533) Percent (95%CI)
**Buyer**			
Age (years)[a]	19.2 (19.2,19.3)	19.2 (19.2,19.3)	19.2 (19.2,19.3)
Sex			
Female	51.5 (31.3,71.2)	50.4 (30.2,70.4)	52.5 (32.1,72.1)
Male	48.5 (28.8,68.7)	49.6 (29.6,69.8)	47.5 (27.9,67.9)
**Store**			
Location			
On street	36.4 (30.7,42.4)	35.4 (29.0,42.3)	37.3 (30.8,44.4)
In mall	63.6 (57.6,69.3)	64.6 (57.7,71.0)	62.7 (55.6,69.2)
Age-of-sale warning sign			
No	61.5 (53.1,69.2)	62.2 (52.5,71.0)	60.8 (52.6,68.4)
Yes	38.5 (30.8,46.9)	37.8 (29.1,47.5)	39.2 (31.6,47.4)
License			
No	22.2 (15.7,30.4)	20.7 (14.4,28.8)	23.6 (15.9,33.6)
Yes	77.8 (69.6,84.3)	79.3 (71.2,85.6)	76.4 (66.4,84.1)
E-cigarette health warning			
No	92.0 (89.5,93.9)	92.0 (88.6,94.5)	91.9 (87.8,94.8)
Yes	8.0 (6.1,10.5)	8.0 (5.5,11.4)	8.1 (5.2,12.2)
**Seller**			
Perceived age (years):			
18 to <30	19.2 (15.1,24.0)	18.8 (14.7,23.8)	19.5 (14.8,25.3)
30 to <40	31.2 (26.4,36.4)	29.9 (23.6,36.9)	32.5 (27.7,37.6)
≥40	49.7 (43.4,55.9)	51.3 (44.1,58.5)	48.0 (41.3,54.8)
Perceived sex			
Female	58.3 (55.0,61.4)	56.1 (51.4,60.7)	60.4 (56.0,64.7)
Male	41.7 (38.6,45.0)	43.9 (39.3,48.6)	39.6 (35.3,44.0)
**Other**			
Region			
Western	30.5 (20.3,43.1)	29.9 (19.4,43.0)	31.1 (20.5,44.2)
Central	22.9 (20.6,25.5)	23.2 (19.4,27.4)	22.7 (18.9,27.0)
Eastern	46.5 (34.3,59.3)	47.0 (34.2,60.1)	46.1 (33.7,59.1)
Day of visit			
Workday	65.4 (62.9,67.9)	65.8 (60.0,71.2)	65.1 (60.2,69.7)
Weekend	34.6 (32.1,37.1)	34.2 (28.9,40.0)	34.9 (30.3,39.8)

[a] The mean of buyers' ages is reported.

© 2025 Wang Y et al. *JAMA Network Open*.

eTable 2: Multivariate Logistic Regressions for Successful Purchases, Inquiring About Age, Requesting ID Card, and Dissuading Use, Cigarette Store Visits, China

	Successful Purchase (N=1059)		Inquiring about age (N=1059)		Requesting ID card (N=1059)		Dissuading E-cigarette use (N=1044)[a]	
**Characteristics**	Adjusted odds ratio (95% CI)	P-Value	Adjusted odds ratio (95% CI)	P-Value	Adjusted odds ratio (95% CI)	P-Value	Adjusted odds ratio (95% CI)	P-Value
**Buyer**								
Attire type								
Casual attire	Reference		Reference		Reference		Reference	
School uniforms	1.40 (0.65,2.98)	0.39	24.36 (7.34,80.84)	p<0.001	4.30 (0.99,18.74)	0.052	11.03 (1.54,78.77)	0.02
Age (years)	2.20 (0.59,8.21)	0.24	1.79 (0.98,3.28)	0.059	1.25 (0.31,5.05)	0.76	0.59 (0.13,2.65)	0.49
Sex								
Female	Reference		Reference		Reference		Reference	
Male	1.49 (0.56,3.97)	0.42	0.94 (0.56,1.58)	0.83	0.94 (0.29,3.10)	0.92	0.27 (0.07,1.08)	0.06
**Store**								
Location								
On street	Reference		Reference		Reference		Reference	
In mall	0.96 (0.59,1.59)	0.89	1.21 (0.72,2.04)	0.46	1.77 (0.34,9.10)	0.49	2.35 (0.49,11.27)	0.28
Age-of-sale warning sign	1.19 (0.75,1.91)	0.46	2.37 (1.45,3.89)	p<0.001	1.42 (0.35,5.85)	0.63	0.77 (0.19,3.12)	0.72
License	1.10 (0.61,1.99)	0.75	0.61 (0.27,1.39)	0.24	2.28 (0.21,25.33)	0.5	0.41 (0.10,1.61)	0.20
E-cigarette health warning	1.20 (0.46,3.09)	0.71	1.15 (0.43,3.09)	0.78	1.06 (0.11,10.22)	0.96	4.35 (0.74,25.42)	0.10
**Seller**								
Perceived age (years):								
18 to <30	Reference		Reference		Reference		Reference	
30 to <40	1.98 (1.18,3.32)	0.009	0.65 (0.31,1.35)	0.25	0.53 (0.11,2.43)	0.41	1.48 (0.31,7.00)	0.62
≥40	1.43 (0.71,2.89)	0.32	0.33 (0.18,0.59)	p<0.001	0.08 (0.01,0.62)	0.02	0.29 (0.03,2.41)	0.25
Perceived sex								
Female	Reference		Reference		Reference		Reference	
Male	0.73 (0.41,1.30)	0.29	1.31 (0.67,2.53)	0.43	1.07 (0.31,3.73)	0.92	2.45 (0.52,11.47)	0.26
**Other**								
Region								
Western	Reference		Reference		Reference		Reference	
Central	0.67 (0.29,1.56)	0.35	0.95 (0.34,2.67)	0.93	0.53 (0.10,2.68)	0.44	2.00 (0.35,11.44)	0.44
Eastern	0.50 (0.16,1.55)	0.23	0.99 (0.42,2.34)	0.98	0.58 (0.11,2.97)	0.51	0.68 (0.16,2.94)	0.61
Day of visit								
Workday	Reference		Reference		Reference		Reference	
Weekend	1.07 (0.64,1.79)	0.80	0.83 (0.45,1.51)	0.54	0.88 (0.16,4.92)	0.88	0.62 (0.19,2.02)	0.43
**Inquiring about age**	0.15 (0.06,0.39)	p<0.001						
**Requesting ID card**	0.16 (0.04,0.63)	0.009						

[a] Adolescent buyers who were asked to leave the store immediately upon entry were excluded from the analysis (n=15; all were in school uniforms), as no buyer-seller communication occurred.

© 2025 Wang Y et al. *JAMA Network Open*.

eTable 3: Parallel Mediation Analysis for Requesting ID Card and Inquiring About Age[a]

	Coefficient	Std. err.	z	P>z	95% CI
Total effect	-2.32	0.266	-8.69	p<0.001	(-2.84, -1.79)
Direct effect	-0.94	0.275	-3.41	0.001	(-1.48, -0.40)
Indirect effect	-1.38	0.175	-7.88	p<0.001	(-1.72, -1.04)
via Requesting ID card	-1.17	0.130		p<0.001	
via inquiring about age	-0.20	0.163		0.21	

[a] The independent variable is whether the adolescent buyer was wearing a school uniform, and the dependent variable is whether the purchase of e-cigarettes was successful.

© 2025 Wang Y et al. *JAMA Network Open*.

eTable 4: Subgroup Analyses of Successful Purchases, Inquiring About Age, Requesting ID Card, and Dissuading Use Across Sex, Age, and Region

	**Successful Purchase (N=1089)**		**Inquiring about age (N=1089)**		**Requesting ID card (N=1089)**		**Dissuading e-cigarette use (N=945)[a]**		
	Adjusted odds ratio (95% CI)	P-Value	Adjusted odds ratio (95% CI)	P-Value	Adjusted odds ratio (95% CI)	P-Value		Adjusted odds ratio (95% CI)	P-Value
**Subgroup**									
**Sex**									
Female (N=563)	0.40** (0.21,0.79)	0.008	8.88*** (5.65,13.95)	p<0.001	4.98*** (3.29,7.54)	p<0.001	N=454[b]	1.86** (1.22,2.83)	0.004
Male (N=526)	0.43 (0.16,1.20)	0.11	10.38*** (6.19,17.42)	p<0.001	10.40*** (5.40,20.04)	p<0.001	N=491[b]	1.83* (1.09,3.09)	0.02
**Age**									
18 (N=279)	0.50*** (0.41,0.60)	p<0.001	9.07*** (6.40,12.84)	p<0.001	10.21*** (5.87,17.76)	p<0.001	N=252[b]	1.25 (0.90,1.73)	0.19
19 (N=810)	0.32** (0.15,0.66)	0.002	9.47*** (5.96,15.03)	p<0.001	6.32*** (4.03,9.93)	p<0.001	N=693[b]	2.07** (1.32,3.25)	0.002
**Region**									
Western (N=332)	0.44* (0.20,0.98)	0.04	11.32*** (5.62,22.77)	p<0.001	7.01*** (3.82,12.85)	p<0.001	N=428[b]	1.59 (0.94,2.70)	0.08
Central (N=253)	0.25* (0.07,0.86)	0.03	9.92*** (5.27,18.69)	p<0.001	16.09*** (5.21,49.74)	p<0.001	N=228[b]	1.63 (0.85,3.15)	0.14
Eastern (N=504)	0.34* (0.14,0.82)	0.02	6.77*** (3.96,11.58)	p<0.001	4.36*** (2.16,8.77)	p<0.001	N=289[b]	2.30* (1.02,5.18)	0.04

[a] A few adolescent buyers who were asked to leave the store might still have been inquired about their age or asked to present identification. Those who were asked to leave without any further buyer–seller communication were excluded from the analysis.

[b] The sample sizes are reported specifically for the regression analyses related to dissuading e-cigarette use.

© 2025 Wang Y et al. *JAMA Network Open*.

eFigure 1: A Sampling Distribution Map of E-cigarette Stores

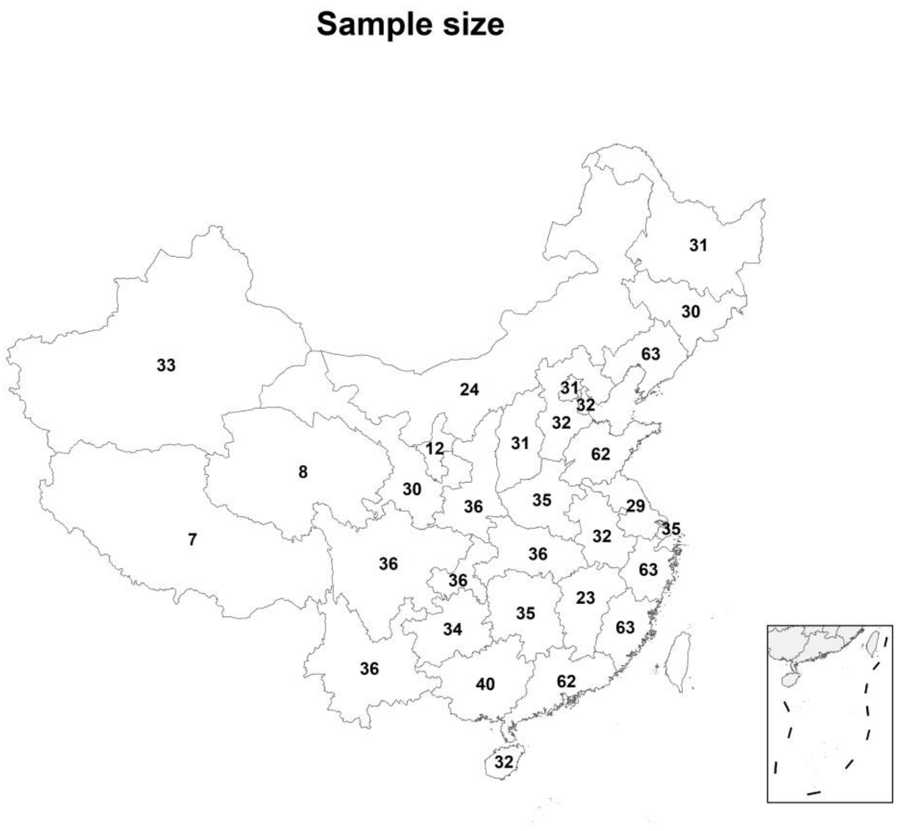

Legend: Each number on the map represents the number of e-cigarette stores visited in that province.

© 2025 Wang Y et al. *JAMA Network Open*.

eFigure 2: Male and Female Models in School Uniforms and Casual Attire

School uniform, male

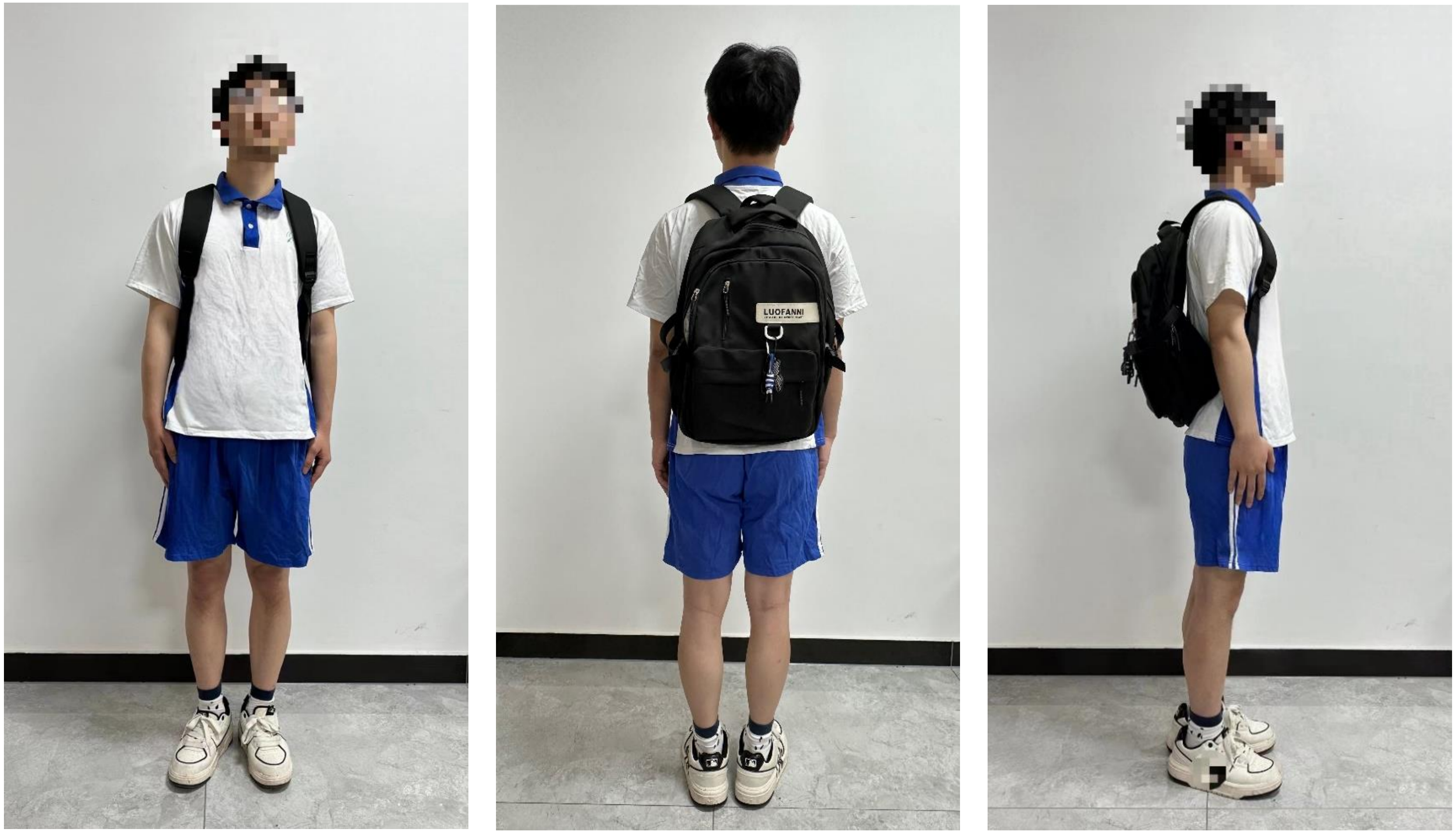

© 2025 Wang Y et al. *JAMA Network Open*.

School uniform, female

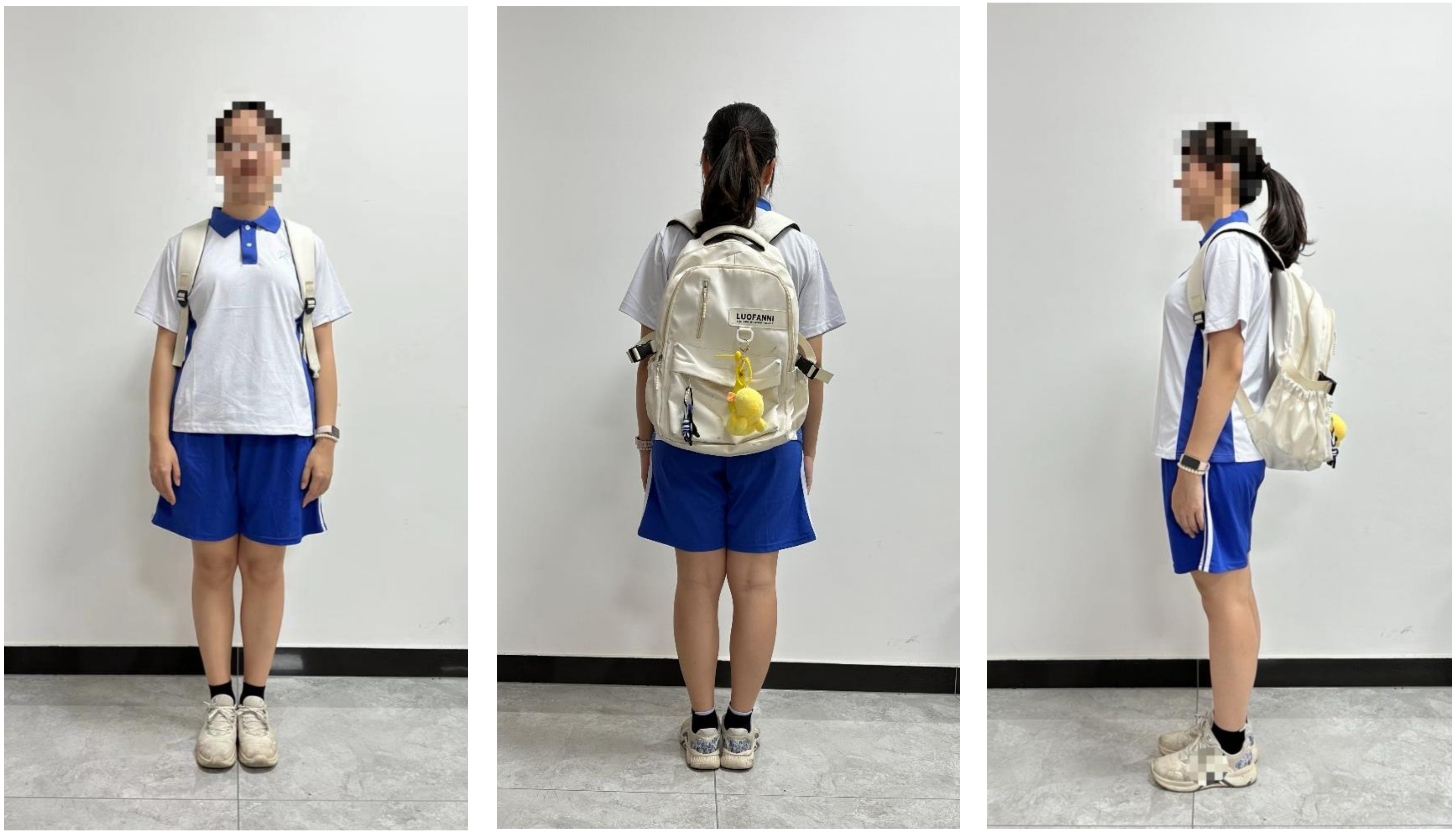

© 2025 Wang Y et al. *JAMA Network Open*.

Casual attire, male

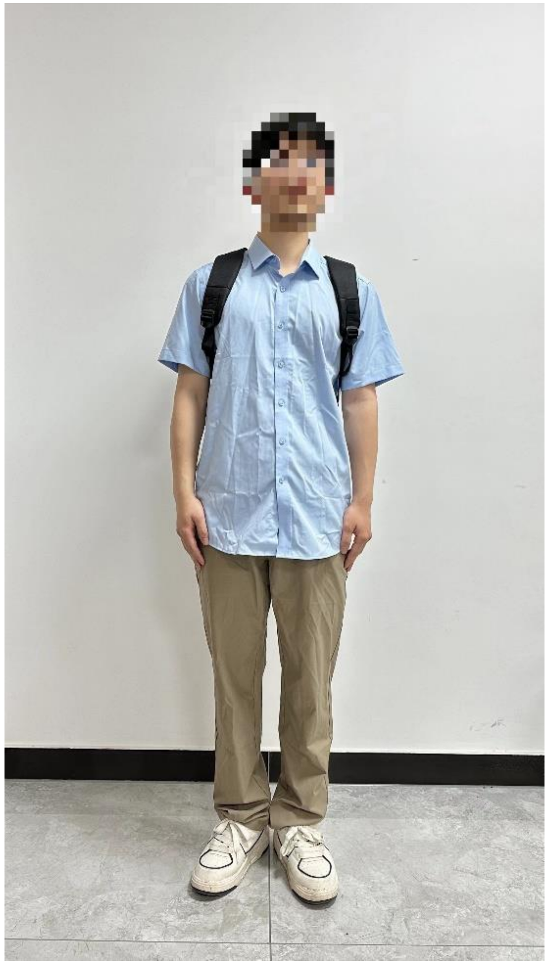

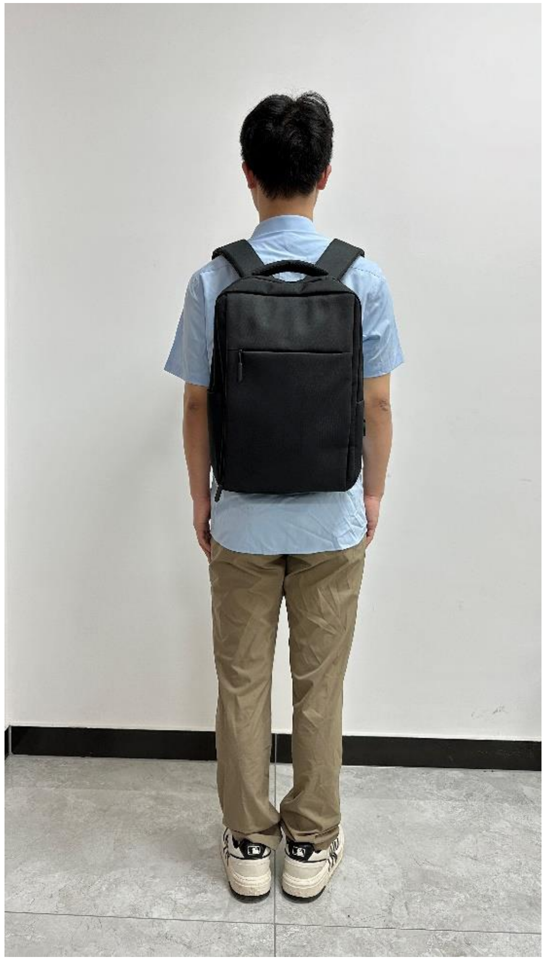

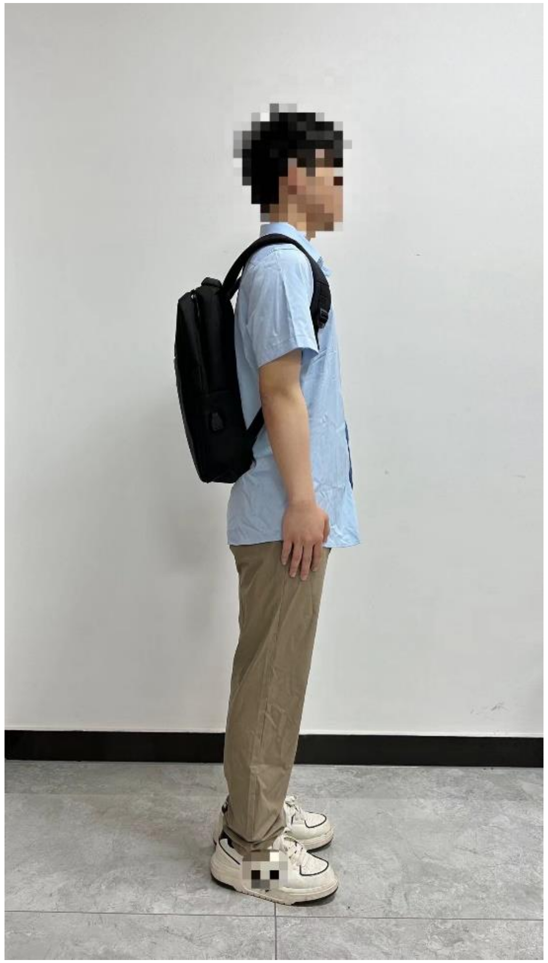

© 2025 Wang Y et al. *JAMA Network Open*.

Casual attire, female

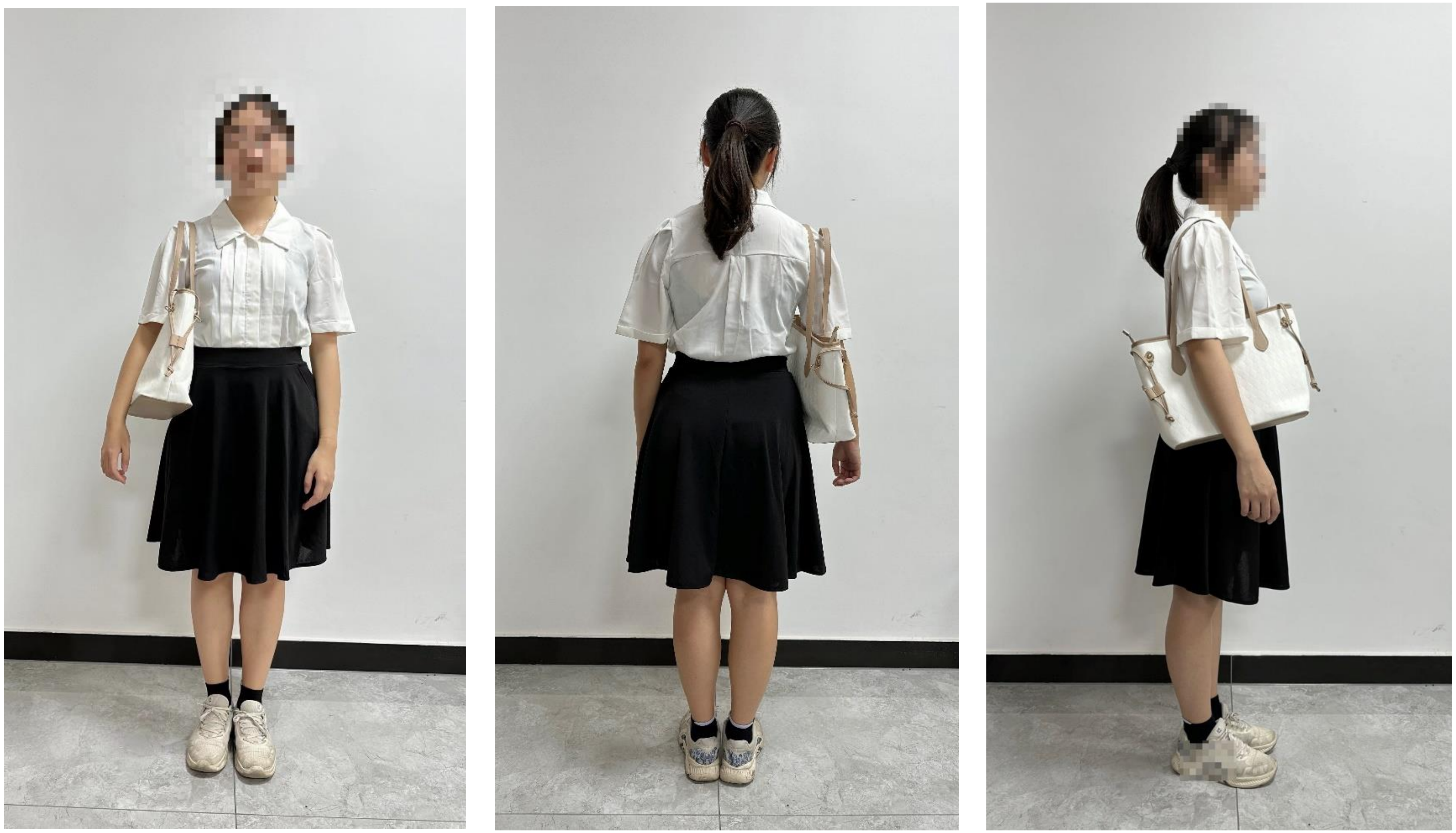

© 2025 Wang Y et al. *JAMA Network Open*.

eFigure 3: Differences in Successful E-cigarette Purchases, Stratified by Types of Attire and Age-Verification Behaviors

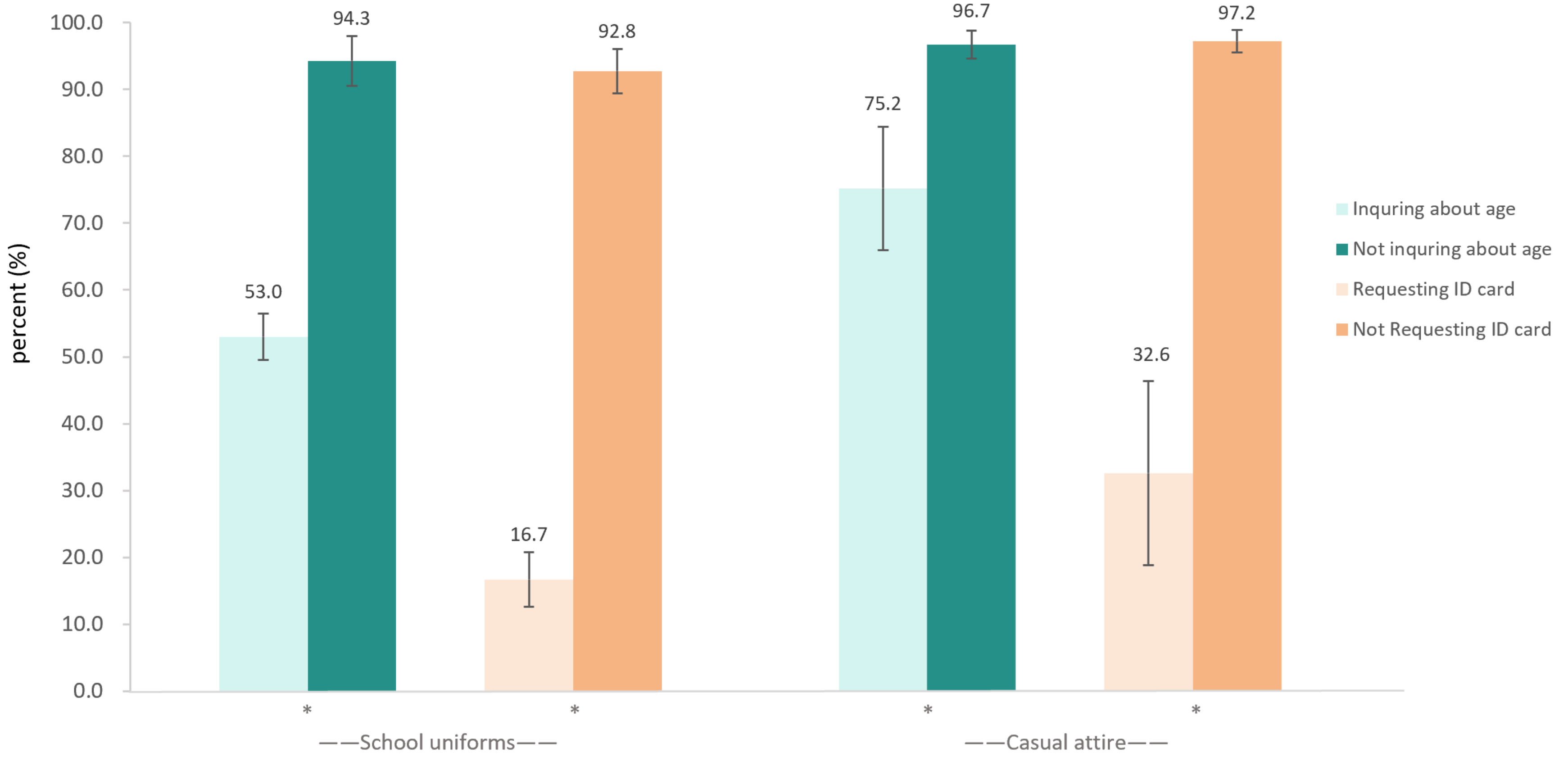

*P-value <0.001

Legend: Bars indicate the percentage of successful e-cigarette purchases, stratified by seller age-verification behaviors and the type of attire worn by adolescent buyers. The likelihood of e-cigarette sales to adolescents was significantly lower among sellers who inquired about the buyers' ages and/or requesting ID cards compared to those who did not.

© 2025 Wang Y et al. *JAMA Network Open*.

eFigure 4: Differences in Cigarette Sales to Adolescents by Types of Attire, China

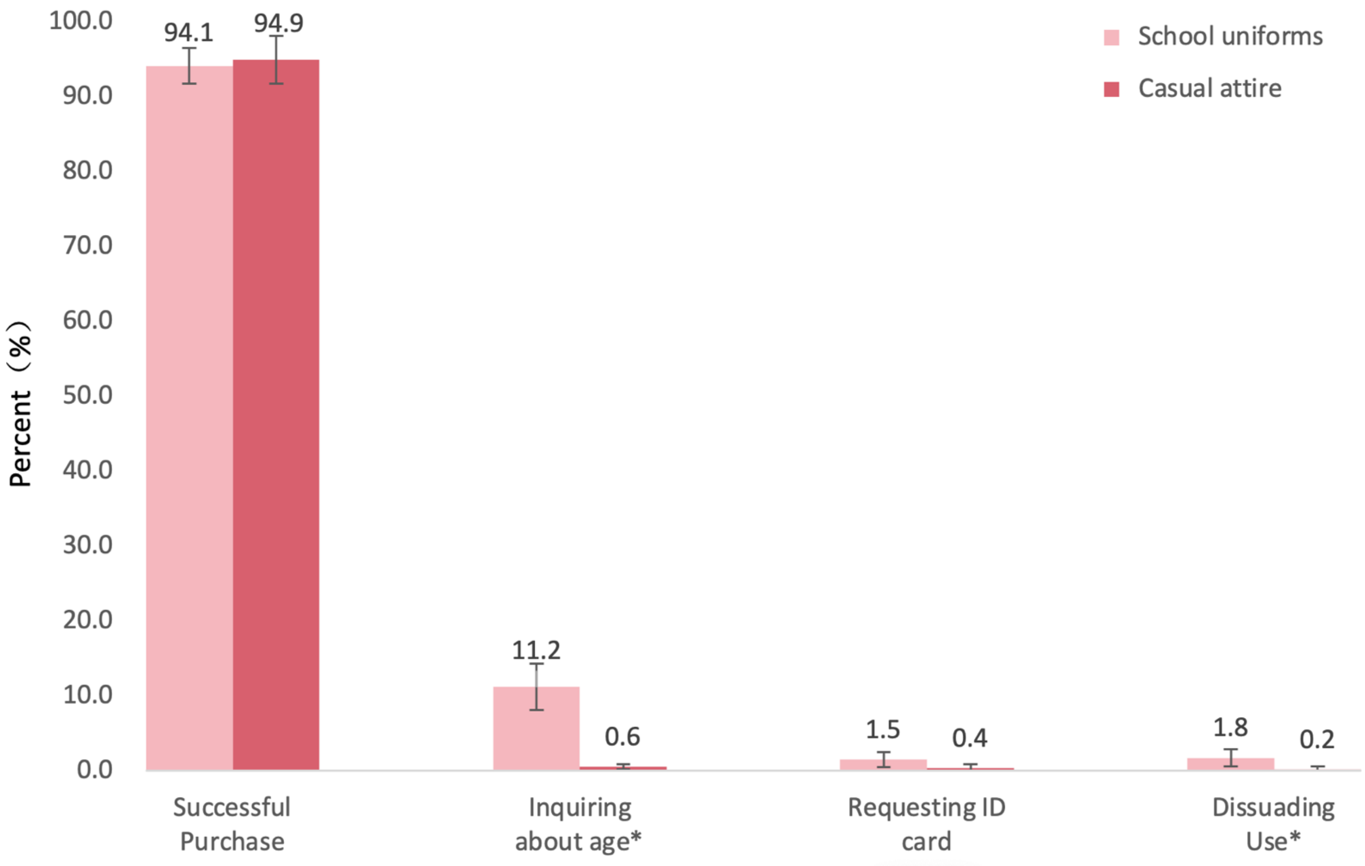

*P-value <0.05

Legend: Bars indicate the percentage of successful cigarette purchases and seller behaviors among adolescent buyers wearing school uniforms versus casual attire. Although wearing school uniform insignificantly reduced the likelihood of successful purchases and increased the likelihood of requesting ID card, it was significantly associated with an increasing probability of inquiring about age and dissuading e-cigarette use.

© 2025 Wang Y et al. *JAMA Network Open*.

eFigure 5: Maps of Likelihood of Successful Purchases, Inquiring About Age, Requesting ID Card, and Dissuading Use in E-cigarette Stores, China (N=1089)

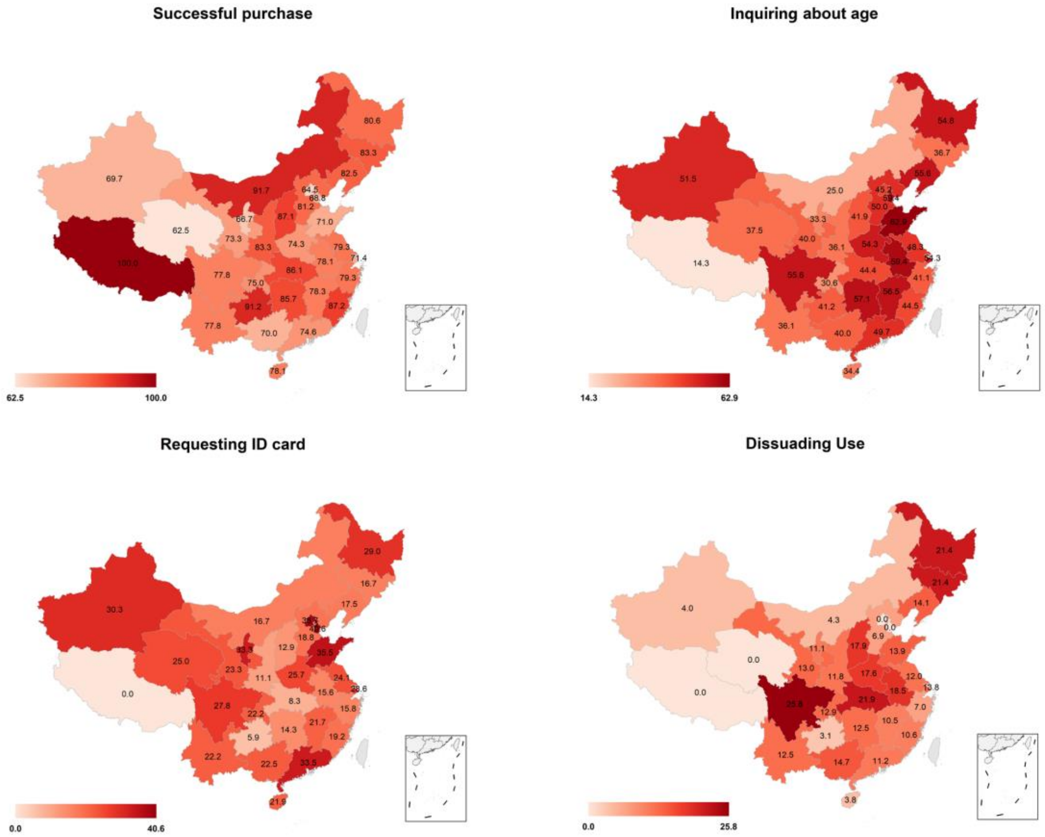

Legend: Each number represents the likelihood of the corresponding buyer and seller behavior in that province. The sample for the analysis of dissuading uses of e-cigarettes was 945. Adolescent buyers who were asked to leave the store immediately upon entry were excluded from this analysis, as no buyer-seller communication occurred.

© 2025 Wang Y et al. *JAMA Network Open*.
