# Supplementary material for: E-Cigarette Sales to School-Uniformed Adolescents in China: A Randomized Clinical Trial
Source: JAMA Netw Open. 2025 Oct 10;8(10):e2535623. doi: 10.1001/jamanetworkopen.2025.35623 (PMC12514632; doi:10.1001/jamanetworkopen.2025.35623)
Supplement: Supplement 3. — Data Sharing Statement [file jamanetwopen-e2535623-s003.pdf]

Data Sharing Statement

Wang. e-Cigarette Sales to School-Uniformed Adolescents in China. *JAMA Netw Open*. Published October 10, 2025. doi:10.1001/jamanetworkopen.2025.35623

Data
Additional Information: ClinicalTrials.gov Identifier: NCT05962411.
https://clinicaltrials.gov/study/NCT0596241162411
Data available: No

Additional Information
Explanation for why data not available: No data are available because the study involved undercover fieldwork using simulated clients to assess regulatory compliance among tobacco retailers.
